# Identification and Characterization of Novel DPP-IV Inhibitory Peptides from *Limnospira platensis* Hydrolysates: Stability and Intestinal Permeability Evaluation

**DOI:** 10.3390/foods15162838

**Published:** 2026-08-14

**Authors:** Kota Ebato, Haruka Kobayashi, Hiroaki Tsutsumi, Yoko Iijima, Kenjiro Sugiyama

**Affiliations:** 1 Applied Chemistry and Chemical Engineering Program, Graduate School of Engineering, Kogakuin University, Hachioji 192-0015, Tokyo, Japan; bm23013@g.kogakuin.jp (K.E.); yiijima@cc.kogakuin.ac.jp (Y.I.); 2 Department of Applied Chemistry, School of Advanced Engineering, Kogakuin University, Hachioji 192-0015, Tokyo, Japan; s221036@g.kogakuin.jp; 3 Holistic Beauty Research Center, Wamiles Cosmetics Inc., Yokohama 233-0003, Kanagawa, Japan; hiroaki.tsutsumi@wamiles.co.jp

**Keywords:** bioactive peptide, dipeptidyl peptidase-IV (DPP-IV), *Limnospira*, type 2 diabetes, protein hydrolysate

## Abstract

As the prevalence of type 2 diabetes increases rapidly, the demand for natural origin dipeptidyl peptidase-IV (DPP-IV) inhibitors with fewer side effects is increasing. In this study, protein-rich *Limnospira platensis* was investigated as a source of bioactive peptides to enhance its value as a functional food ingredient. Hydrolysates were prepared using three food-processing proteases, individually and in two-step combinations, followed by in silico analysis and peptide identification via liquid chromatography–tandem mass spectrometry. Subsequently, the thermal stability, gastrointestinal resistance, and intestinal permeability (using Caco-2 cells) of the identified peptides were evaluated. It was revealed that the Orientase 22BF digest exhibited high DPP-IV inhibitory activity. From the digest, three novel peptides—SPSPN (IC_50_ = 144.1 ± 2.2 μM), VPSV (IC_50_ = 93.6 ± 5.8 μM), and IPIGG (IC_50_ = 13.4 ± 2.3 μM)—were identified, exhibiting DPP-IV inhibitory potencies comparable to or higher than previously reported *Limnospira*-derived peptides. Although VPSV exhibited low epithelial permeability (P_app_ = 4.02 ± 0.69 × 10^−8^ cm/s), it remained stable under simulated gastrointestinal digestion conditions, suggesting potential local luminal inhibitory activity within the small intestine. Overall, these findings highlight *Limnospira*-derived VPSV as a promising functional ingredient candidate with high bioactivity and digestive stability.

## 1. Introduction

Diabetes represents a major public health concern, with its prevalence increasing rapidly worldwide. Data from the International Diabetes Federation indicates that the prevalence of diabetes is projected to reach 537 million adults by 2021, representing ~10.5% of the global adult population [1]. Moreover, this number is projected to increase to 783 million by the year 2045, with the majority of cases constituting type 2 diabetes mellitus. Type 2 diabetes is a complex disease of multifactorial origin, involving genetic influences such as impaired insulin secretion and insulin resistance, which are affected by diet and physical inactivity. This results in a relative insulin deficiency that contributes to disease progression [2].

Several therapeutic agents have been developed for the treatment of type 2 diabetes, including inhibitors of dipeptidyl peptidase IV (DPP-IV), a serine protease that exists in both membrane-bound and soluble forms. DPP-IV is widely expressed in many cell types, particularly in the epithelial tissues of the liver, gut, and kidneys [3]. It preferentially cleaves peptides containing proline (Pro) or alanine (Ala) at the penultimate N-terminal position [2]. Additionally, this enzyme helps break down incretins and hormones in the gastrointestinal tract, which stimulate insulin secretion in response to food. Consequently, blocking DPP-IV prolongs incretin activity, increases insulin secretion, and helps regulate blood sugar levels after meals [4].

In 2006, sitagliptin, a DPP-IV inhibitor, was the first synthetic drug to be approved by the United States Food and Drug Administration for diabetes treatment [5]. Since then, a number of synthetic gliptin drugs, including vildagliptin, saxagliptin, and linagliptin, have been developed and are currently used in clinical practice [6]. However, these synthetic DPP-IV inhibitors have been reported to cause adverse effects, such as gastrointestinal disorders, allergic reactions, skin-related problems, and musculoskeletal issues [2]. It is therefore necessary to develop safer, alternative treatments.

In recent years, numerous in vitro and in vivo studies have revealed that food-derived components, particularly peptides, can inhibit DPP-IV activity [7,8], with >200 DPP-IV inhibitory peptides identified in various food proteins [2]. Many of these peptides are derived from dairy products, including casein and whey protein [9], and they demonstrate notable potency. As a result, there is growing interest in food-derived peptide inhibitors as potential therapeutic agents with minimal side effects.

*Spirulina*, currently classified as *Limnospira platensis* and previously known as *Arthrospira platensis* or *Spirulina platensis*, is a filamentous cyanobacterium [10]. This microalga is widely cultivated for human consumption and as animal feed because of its rich nutritional profile, which includes a high protein content of 60–70% (dry weight basis). *Limnospira platensis* contains all essential amino acids, along with beneficial compounds such as unsaturated fatty acids (e.g., γ-linolenic acid), vitamins, minerals, and pigments such as carotenoids and phycocyanin [11]. From an industrial farming perspective, *Limnospira platensis* offers several benefits, including its preference for highly alkaline environments, which naturally reduces the risk of contamination. Additionally, its long spiral shape aids harvesting by filtration [12]. Previous research has shown that protein hydrolysates from *Limnospira* contain peptides that exhibit antioxidant, antihypertensive, anti-inflammatory, anti-obesity, and antibacterial effects [13,14,15,16,17]. Additionally, extracts and hydrolysates from *Limnospira* have been found to exhibit DPP-IV inhibitory activity [18,19,20]. Recent studies have successfully identified specific DPP-IV inhibitory peptides from *L. platensis*, such as GVPMPNK, LRSELAAWSR, and RNPFVFAPTLLTVAAR [18] and phycocyanin-derived GPNYASSER [20]. However, most previous efforts have focused on single-enzyme hydrolysis or specific proteins, leaving a gap in understanding how systematic multi-enzyme hydrolysis approaches affect peptide potency, structural features, gastrointestinal stability, and intestinal permeability.

The current study therefore aims to identify novel DPP-IV inhibitory peptides from *Limnospira* protein hydrolysates and evaluate their functional properties, including their thermal and digestive stabilities and intestinal permeability. Although many previous studies have focused on the hydrolysates produced by a single enzyme, this study systematically evaluates the effects of three food-processing proteases, both individually and in two-stage combinations. By assessing their stability against gastrointestinal enzymes and their ability to cross the intestinal barrier, the potential of these peptides as functional food ingredients is evaluated. Overall, this work aims to provide a deeper understanding of the role of raw material and processing method selection in the development of bioactive peptides for diabetes management.

## 2. Materials and Methods

### 2.1. Materials and Reagents

*Limnospira platensis* was obtained and cultivated as previously described [21]. Caco-2 cells (RGB0988 cells) were purchased from RIKEN BRC (Tsukuba, Japan). Orientase 22BF and Orientase OP were acquired from HBI Enzymes, Inc. (Kobe, Japan). Papain, pepsin, penicillin, streptomycin, and non-essential amino acids were purchased from FUJIFILM Wako Pure Chemical Corporation (Osaka, Japan). Trypsin, α-chymotrypsin, and low-glucose Dulbecco’s modified Eagle medium (DMEM) were obtained from Merck KGaA (Darmstadt, Germany). Fetal bovine serum was purchased from Serena Europe GmbH (Pessin, Germany). Seven peptides containing the sequences APAPQ, SPSPN, VPSV, VPVS, IPGAT, IPIN, and IPIGG were synthesized on demand by GenScript, Inc. (Nanjing, China) and purified to >95% purity.

### 2.2. Protein Extraction from Limnospira Cells

*Limnospira* cells were collected by filtration through a 75 µm sieve, washed with pure water, and lyophilized. Dried *Limnospira* powder was dissolved in pure water and vortexed to form a suspension (30 mg/mL). The suspension was subjected to three freeze–thaw cycles using liquid nitrogen at 25 °C. The samples were then stored on ice and subjected to ultrasonication (66 W, 6 s ON/9 s OFF) for 8 min. After ultrasonic treatment, the suspension was centrifuged at 4 °C and 10,000× *g* for 30 min; the supernatant was collected, and ammonium sulfate was added. The resulting mixture was stirred overnight under cooling, then centrifuged again at 4 °C and 15,000× *g* for 30 min. After dissolving the precipitate in pure water, the solution was dialyzed overnight against pure water using a dialysis membrane, then lyophilized and stored at −20 °C until required for use.

### 2.3. Preparation of Limnospira Protein Hydrolysates

*Limnospira* protein was hydrolyzed using three individual proteases (Orientase 22BF, Orientase OP, and Papain) and their sequential combinations (Orientase 22BF-then-Orientase OP, Orientase 22BF-then-Papain, and Orientase OP-then-Papain). The reaction conditions (pH 8.0 and 55 °C) were selected as the overlapping optimal range for all three commercial proteases. Specifically, *Limnospira* protein powder was dissolved in 0.2 M phosphate buffer (pH 8.0) to give a concentration of 10 mg/mL. The protease was added to the solution at the following enzyme-to-substrate ratios: 2.0% (*w*/*w*) Orientase 22BF, 0.5% (*w*/*w*) Orientase OP, and 6.4% (*w*/*w*) Papain. For the single-protease treatments, the reaction mixture was incubated at 55 °C for 4 h with shaking at 1000 rpm. The reaction time for each protease was determined based on the time-course hydrolysis profile (Figure S1). For sequential hydrolysis, the first protease was added, and the mixture was incubated at 55 °C for 4 h with shaking at 1000 rpm prior to heat-inactivation at 95 °C for 10 min. After cooling the solution to 55 °C, the second protease was added, and the mixture was incubated for an additional 4 h at 55 °C with shaking at 1000 rpm. All enzymatic reactions were terminated by heating at 95 °C for 10 min. The resulting hydrolysates were subjected to centrifugation at 4 °C and 15,000× *g* for 10 min, and the supernatant was lyophilized and stored at −20 °C for further analysis. As an undigested control, the protein solution was incubated under identical conditions in the presence of protease that had been pre-inactivated by heating at 95 °C for 20 min.

### 2.4. DPP-IV Inhibitory Assay

DPP-IV inhibition was assessed using a DPP-IV screening assay kit (Cayman Chemical, Ann Arbor, MI, USA), according to the manufacturer’s instructions. Reagent solutions were prepared in 1 × DPP-IV Assay Buffer: 5 × DPP-IV enzyme was diluted 1:4 (*v*/*v*), and DPP-IV Substrate was diluted 1:24 (*v*/*v*). Assays were conducted in 96-well black plates with a total volume of 50 µL/well. Briefly, each well contained 15 µL of assay buffer, 5 µL of sample solution (or purified water for 100% activity and background controls), 5 µL of enzyme solution (or buffer for background controls), and 25 µL of substrate solution. Background wells (F_B_) were included to account for buffer background and sample autofluorescence. After incubation at 37 °C for 30 min, fluorescence was measured using a Synergy H1 microplate reader (Agilent Technologies, Santa Clara, CA, USA) at excitation/emission wavelengths of 355/460 nm (gain: 50). Assays were performed in triplicate. The inhibition percentage was calculated as

(1) % Inhibition = (F_A_ − F_S_)/(F_A_ − F_B_) × 100

where F_A_, F_S_, and F_B_ represent the fluorescence intensities of the 100% initial activity well, the sample well, and the background well, respectively.

### 2.5. Separation of DPP-IV Inhibitory Peptides from Limnospira Protein Hydrolysates

#### 2.5.1. Ultrafiltration

*Limnospira* protein hydrolysate powder was dissolved in pure water and the resulting solution was separated by ultrafiltration (Amicon Ultra-15 ultrafiltration membrane, 3 kDa molecular weight cut-off (MWCO), Merck KGaA) to obtain the ultrafiltrate. The <3 kDa fraction was then lyophilized.

#### 2.5.2. Reversed-Phase High-Performance Liquid Chromatography

The <3 kDa fraction was dissolved in solvent A (water containing 0.1% trifluoroacetic acid) and separated by reversed-phase high-performance liquid chromatography (RP-HPLC, Shimadzu Corporation, Kyoto, Japan) on a C18 column (250 × 20 mm, 5 μm, Shimadzu Corporation). Gradient elution with solvent A (water containing 0.1% trifluoroacetic acid) and solvent B (acetonitrile containing 0.1% trifluoroacetic acid) was performed as follows: 0–120 min, 0.1–30% B; 120–150 min, 30% B; 150–151 min, 30–0.1% B; and 151–230 min, 0.1% B. The injection volume was 1000 μL, the flow rate was 5 mL/min, and the detection wavelength was 210 nm. The separated fractions were collected in test tubes at 3 min intervals between retention times of 33 and 114 min. The large early peak (10–17 min), corresponding to the column void volume containing unretained salts and highly polar impurities, was excluded from fraction collection. Due to the extremely low peptide recovery in individual fractions, which was below the limit for precise gravimetric measurement, the fractions were evaluated on a starting-material-equivalent basis. Each collected fraction was lyophilized and reconstituted in an identical volume (150 µL) of 0.2 M phosphate buffer (pH 8.0). This standardized preparation allowed a direct comparison of the relative DPP-IV inhibitory activity contributed by each fraction derived from a constant amount of the starting hydrolysate.

### 2.6. Identification of DPP-IV Inhibitory Peptides by Liquid Chromatography–Tandem Mass Spectrometry

The peptides present in the fractions exhibiting potent DPP-IV inhibitory activity were identified using a Vanquish ultra-high-performance liquid chromatography (UHPLC) system (Thermo Fisher Scientific, Waltham, MA, USA) coupled to an Orbitrap Exploris 120 mass spectrometer (Thermo Fisher Scientific) equipped with an electrospray ionization (ESI) source. An Acclaim^TM^ 120 C18 column (100 × 2.1 mm, 3 μm, Thermo Fisher Scientific) was equilibrated with solvent A (water containing 0.1% formic acid) and solvent B (acetonitrile containing 0.1% formic acid), and gradient elution was performed as follows: 0–5 min, 2% B; 5–25 min, 2–30% B; 25–55 min, 30–90% B; 55–60 min, 90% B; 60–60.1 min, 90–2% B; and 60.1–70 min, 2% B. The injection volume was 5 μL, the flow rate was 0.2 mL/min, and the column temperature was maintained at 40 °C. The ESI source was operated in positive ionization mode with a spray voltage of 3.5 kV, an ion transfer tube temperature of 320 °C, and a vaporizer temperature of 275 °C. Full-scan mass spectra (*m*/*z* 200–1500) were acquired using the Orbitrap instrument at a resolution of 60,000. The four most intense precursor ions were selected for fragmentation by high-energy collisional dissociation (HCD) with a collision energy of 30%, and the resulting tandem mass spectrometry (MS/MS) profiles were collected at a resolution of 15,000.

Peptide characterization was performed using Proteome Discoverer software (version 2.5, Thermo Fisher Scientific) with the Sequest HT search engine against the UniProtKB database for *Limnospira platensis* NIES-39 (Taxon ID: 696747, downloaded in July 2022). The search was conducted with no-enzyme specificity (unspecific cleavage). The precursor and fragment mass tolerances were set to <10 ppm and <0.02 Da, respectively. Fixed modifications: None was set as a fixed modification, whereas Variable modifications: Oxidation (M) were specified as variable modifications. Sequence identifications were validated using the Percolator node, accepting peptide identifications with High and Medium confidence filtered at a false discovery rate (FDR) threshold of <5.0%. A comprehensive list of all ~2118 identified peptide candidates, including their modifications, calculated *m*/*z*, observed *m*/*z*, mass error (ΔM in ppm), charge states, abundance, Xcorr, and confidence, is provided in Table S1.

### 2.7. Quantification of the Identified Peptides

Quantitative analysis of the three identified peptides (SPSPN, VPSV, and IPIGG) was performed in the selected ion monitoring mode on the LC-MS/MS system. The chromatographic and mass spectrometric conditions were consistent with those used for peptide identification, except for the analysis of VPSV in the stability and transport assay. To enhance analytical throughput for these assays, the flow rate was increased to 0.3 mL/min, and an accelerated 35 min gradient profile was employed: 0–2 min, 2% B; 2–12 min, 2–45% B; 12–18 min, 45–65% B; 18–20 min, 65–90% B; 20–25 min, 90% B; 25–30 min, 90–2% B; and 30–35 min, 2% B. Standard peptide solutions were prepared to construct calibration curves with high linearity (R^2^ > 0.99) for all peptides, ensuring that all sample quantification values fell within the validated linear range. Subsequently, three independently prepared *Limnospira* hydrolysate batches, heat-treated synthetic peptides, enzyme-digested synthetic peptides, and the basolateral solutions from the Caco-2 cell transport assay were analyzed by monitoring the specific target ion at *m*/*z* 501.2304 for SPSPN ([M+H]^+^), *m*/*z* 401.2395 for VPSV ([M+H]^+^), and *m*/*z* 456.2817 for IPIGG ([M+H]^+^). Data analysis was performed using the Quan Browser in the Xcalibur software (version 4.5, Thermo Fisher Scientific).

### 2.8. Stability of the Identified Inhibitory Peptides

#### 2.8.1. Effect of Heat Treatment

The stability of the DPP-IV inhibitory peptide (VPSV) was evaluated under various heat treatment conditions. For this purpose, a peptide solution (0.5 mg/mL) was heated at 100 °C for 10, 20, 40, and 60 min to mimic cooking conditions, and at 65 °C for 30 min, 75 °C for 15 min, and 121 °C for 15 min to emulate heat sterilization. After treatment, the samples were immediately cooled on ice for 5 min and stored at −20 °C. Undiluted samples were used for the DPP-IV inhibitory activity assay, whereas samples diluted 500-fold with an aqueous solution of 0.1% formic acid were employed for VPSV quantification.

#### 2.8.2. Effects of In Vitro Gastrointestinal Digestion

The VPSV peptide was subjected to simulated digestion using a previously described method with some modifications [22]. Specifically, the peptide (2 mg) was dissolved in pure water (1 mL). The gastric phase was simulated by adding an equal volume of simulated gastric fluid (SGF; 4000 U/mL pepsin, 6.9 mM KCl, 0.9 mM KH_2_PO_4_, 25 mM NaHCO_3_, 47.2 mM NaCl, 0.1 mM MgCl_2_·6H_2_O, 0.5 mM (NH_4_)_2_CO_3_, and 0.15 mM CaCl_2_·2H_2_O; pH 3.0). The resulting mixture was incubated at 37 °C for 2 h with shaking at 1000 rpm. Digestion was terminated by heating at 95 °C for 10 min. Subsequently, an equal volume of simulated intestinal fluid (SIF; 200 U/mL trypsin, 50 U/mL chymotrypsin, 6.8 mM KCl, 0.88 mM KH_2_PO_4_, 85 mM NaHCO_3_, 38.4 mM NaCl, 0.33 mM MgCl_2_·6H_2_O, and 0.6 mM CaCl_2_·2H_2_O; pH 7.0) was added to the mixture. The final pH of the reaction mixture was confirmed to be approximately 7.0. The resulting mixture was incubated at 37 °C for 2 h with shaking at 1000 rpm. Digestion was terminated by heating at 95 °C for 10 min. Subsequently, the reaction mixture was subjected to centrifugation at 4 °C and 15,000× *g* for 10 min. The supernatant was collected and stored at −20 °C. As a control, the same procedure was performed using SGF and SIF solutions in the absence of the respective proteases (pepsin, trypsin, and chymotrypsin). Undiluted samples were used for the DPP-IV inhibitory activity assay, whereas samples diluted 500-fold with an aqueous solution of 0.1% formic acid were used for quantification of VPSV.

### 2.9. Caco-2 Cell Culture

Caco-2 cells were cultured in low-glucose DMEM supplemented with 10% fetal bovine serum, 100 U/mL penicillin and streptomycin, and 1% nonessential amino acids. The medium was changed every 3 d. The cells were subcultured weekly after detachment with trypsin-EDTA and maintained at 37 °C under a 5% CO_2_ atmosphere.

### 2.10. Peptide Transport Assay

Caco-2 cells were seeded into the inserts of a 12-well Transwell plate (0.4 μm pore size, 1 × 10^8^ pores/cm^2^) (Corning, NY, USA) at 1.0 × 10^5^ cells per well. The culture medium was replaced every 2–3 d, and the cells were cultured for 20 d. Monolayer integrity was monitored by measuring the transepithelial electrical resistance (TEER) using a Millicell^®^ ERS-2 system (Merck KGaA). Only monolayers with TEER values exceeding 350 Ω·cm^2^ were used in the peptide transport studies. The cells were washed with HBSS buffer (5.4 mM KCl, 0.44 mM KH_2_PO_4_, 137 mM NaCl, 0.33 mM Na_2_HPO_4_, 1.3 mM CaCl_2_, 1.1 mM MgCl_2_, and 2.5 mM D-glucose) and equilibrated in this buffer for 20 min at 37 °C. Subsequently, an aliquot (0.5 mL) of the peptide solution (1 mM VPSV in HBSS buffer) was added to the apical (insert) side, and HBSS buffer (1.5 mL) was added to the basolateral (well) side. For the transport assay, a peptide concentration of 1 mM in HBSS buffer was used as a uniform test condition to allow direct comparison of permeability among the evaluated peptides within the optimal detection range of LC-MS/MS. The plates were incubated for 2 h at 37 °C under a 5% CO_2_ atmosphere. The basolateral solutions were collected, and the VPSV concentrations in these solutions were measured using the LC-MS/MS method described above. The apparent permeability coefficient (P_app_) of VPSV was calculated as follows:

(2) P_app_ (cm/s) = (ΔQ/Δt)/(A × C_0_)

where ΔQ/Δt is the transport rate of VPSV across the monolayer, A is the device surface area (1.12 cm^2^), and C_0_ is the initial VPSV concentration in the apical side (1 mM).

## 3. Results and Discussion

### 3.1. Selection of the Optimal Protease for Limnospira Hydrolysate Preparation

**Figure 1:**
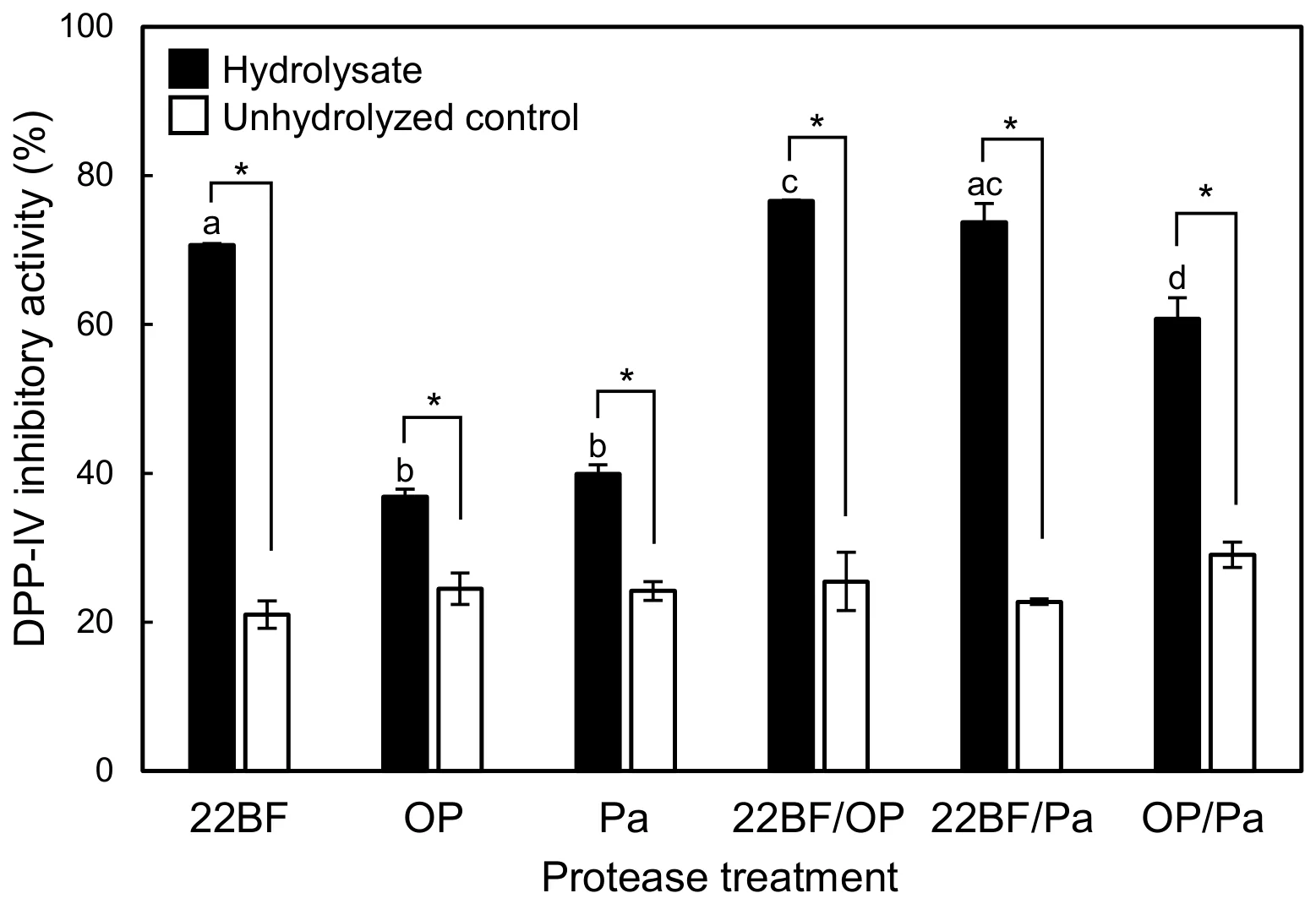
DPP-IV inhibitory activity of *Limnospira* protein hydrolysates and their respective unhydrolyzed controls. 22BF, Orientase 22BF; OP, Orientase OP; Pa, Papain. Filled and open bars represent the hydrolysates and unhydrolyzed controls, respectively. Each value represents the mean ± S.D. (*n* = 3). All reactions were carried out under the overlapping optimal conditions for the proteases (pH 8.0 and 55 °C), with unhydrolyzed controls prepared using prior heat-inactivated enzymes (95 °C for 10 min). Statistical differences were evaluated by two-way analysis of variance (ANOVA) followed by Tukey’s honestly significant difference (HSD) post hoc test. An asterisk (*) indicates a statistically significant increase in activity by hydrolysis compared to each corresponding unhydrolyzed control (*p* < 0.001). Different lowercase letters (a–d) above the bars of the hydrolysates indicate statistically significant differences among the six protease treatments (*p* < 0.05).

To identify the optimal conditions for the efficient production of DPP-IV-inhibitory peptides from *Limnospira* protein, both single-step hydrolysis using three distinct proteases and sequential hydrolysis processes based on two-enzyme combinations were evaluated. To ensure a fair comparison, both the intact *Limnospira* powder (undigested control) and its hydrolysates were prepared from a consistent initial concentration of 10 mg/mL and evaluated at a final reaction concentration of 1 mg/mL in the DPP-IV inhibition assay. The DPP-IV inhibition rates of the hydrolysates produced under each condition are shown in Figure 1. In the single-step process, the hydrolysate produced by Orientase 22BF exhibited the highest activity (71%), followed by papain (40%) and Orientase OP (37%). For the sequential processes, the Orientase 22BF-then-Orientase OP treatment resulted in the highest activity of 77%, followed by the Orientase 22BF-then-Papain (74%) and Orientase OP-then-Papain (61%) treatments. Notably, the activity enhancement in combinations involving Orientase 22BF was marginal, exceeding the activity of Orientase 22BF alone (71%) by only 3–6%. This limited improvement in activity during sequential hydrolysis may be due to “over-hydrolysis.” Specifically, it has been hypothesized that the high-activity peptides generated during the initial enzymatic reaction are further degraded by the second protease, resulting in a loss of activity that offsets the contribution of the newly formed peptides. From an industrial perspective, the thermal inactivation step required after the primary reaction, together with additional enzyme costs and an extended total reaction time, leads to increased manufacturing costs and process complexity. These results therefore suggest that the combined approach is not cost-effective. Thus, hydrolysis using Orientase 22BF alone, which simplifies the process while maintaining high inhibitory activity, was considered optimal and was adopted for subsequent experiments.

### 3.2. Fractionation of the Orientase 22BF Digestion Products and Evaluation of the DPP-IV Inhibitory Activity of Each Fraction

**Figure 2:**
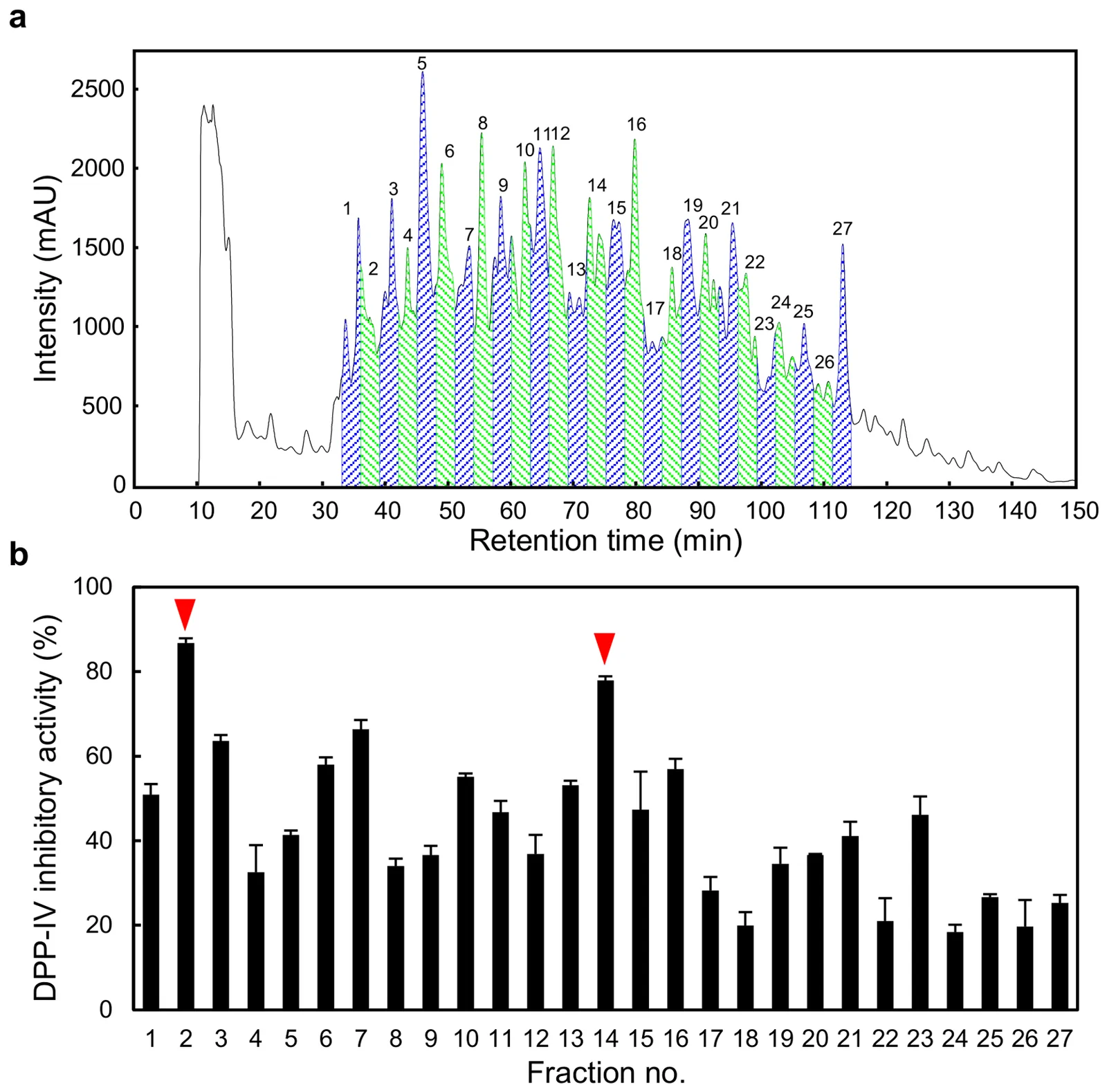
Purification of DPP-IV inhibitory peptides from the <3 kDa fraction of *Limnospira* protein hydrolysate. (**a**) Reverse-phase HPLC chromatogram of the <3 kDa fraction. The numbers correspond to the fraction numbers. Alternating blue and green patterns are used to visually distinguish adjacent fractions. (**b**) DPP-IV inhibitory activities of the 27 fractions collected by RP-HPLC. Each lyophilized fraction was reconstituted in a fixed volume of 0.2 M phosphate buffer (pH 8.0) to assess the total inhibitory potential of each fraction. Each value represents the mean ± S.D. (*n* = 3). Fractions 2 and 14 (marked by red arrows) exhibit the highest inhibitory activities among all fractions and were selected for further analysis.

The 22BF-digested product was fractionated to identify the potential functional peptides. Initially, the digest was separated using an ultrafiltration membrane (3 kDa MWCO) and the permeate (molecular weight < 3 kDa) was collected. The filtrate was further separated into 27 fractions by reversed-phase preparative HPLC (RP-HPLC, Figure 2a), and the DPP-IV inhibitory activity of each fraction was evaluated. As shown in Figure 2b, although several fractions exhibited moderate inhibitory activity, fractions 2 and 14 exhibited outstanding activity exceeding 80%. The high inhibitory activity observed for these fractions could be attributed to either the presence of highly potent peptides, a high abundance of moderately active peptides, or a combination of both. In either case, these fractions represent the most promising sources for the identification of key functional sequences. Therefore, fractions 2 and 14 were selected for further isolation of DPP-IV inhibitory peptides.

### 3.3. Identification of the DPP-IV Inhibitory Peptides

DPP-IV is a serine protease that preferentially cleaves peptides containing proline (Pro) or alanine (Ala) at the second amino acid position from the N-terminus (P1 position). Previous studies have shown that peptides containing Pro at the P1 position competitively bind to the active site of DPP-IV and exhibit strong inhibitory activity [8]. Additionally, since the active site of DPP-IV contains a hydrophobic S1 pocket that specifically accommodates the Pro ring at the P1 position, peptides with higher hydrophobicity indices tend to exhibit greater affinity and inhibitory potential [2]. Furthermore, peptide chain length has been reported to affect the binding capacity, with shorter chains exhibiting higher affinity due to reduced steric hindrance [8]. Candidate peptides were therefore selected based on the following criteria: (1) Peptides containing Pro at the second position from the N-terminus, which is associated with DPP-IV substrate specificity and competitive inhibition; (2) peptides consisting of five or fewer amino acid residues (≤5) to facilitate efficient access to the catalytic pocket; and (3) Peptides with high hydrophobicity, defined as a Wimley-White hydrophobicity score of <7.0 kcal/mol (calculated via PepDraw [https://www.pepdraw.com/] at pH 7.0 with free N-/C-termini, where lower values indicate higher hydrophobicity). Furthermore, to confirm the novelty of the identified sequence candidates, a comprehensive search was performed against the BIOPEP-UWM database (http://www.uwm.edu.pl/biochemia/index.php/en/biopep, accessed on 3 August 2026) to verify that no identical peptide sequences with DPP-IV inhibitory activity had been previously reported.

**Figure 3:**
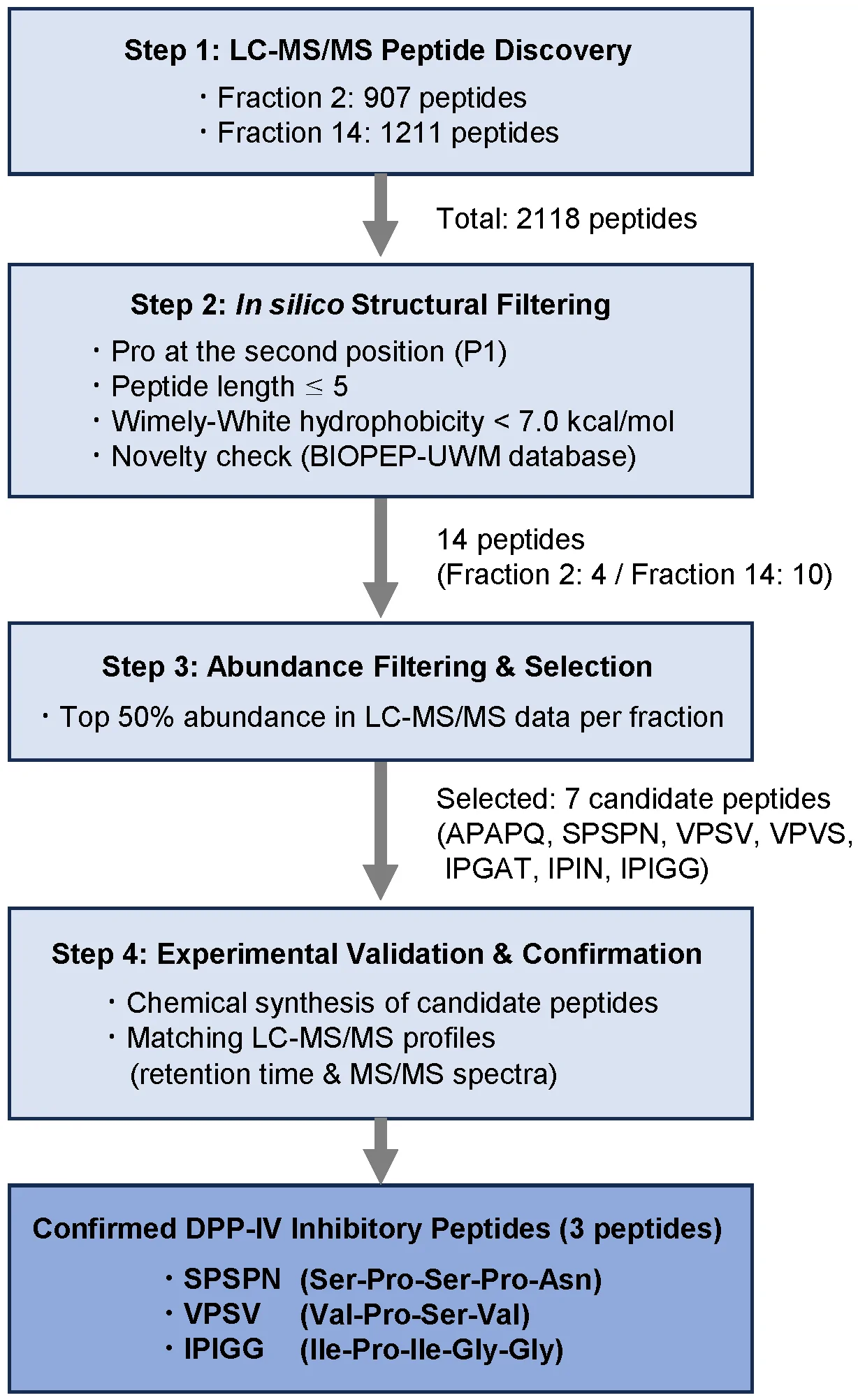
Systematic workflow for screening and identifying DPP-IV inhibitory peptides. The identification process involved four sequential steps: (1) discovery of 2118 unique peptide sequences by LC-MS/MS; (2) in silico filtering based on key structural features (i.e., Pro at the P1 position, peptide length ≤5 residues, and a Wimley-White hydrophobicity score <7.0), yielding 14 candidates (4 from fraction 2 and 10 from fraction 14); (3) selection of the top 50% most abundant candidates from each fraction (2 from fraction 2 and 5 from fraction 14, yielding 7 candidates in total) for chemical synthesis; and (4) experimental validation by comparison of the LC-MS/MS profiles (retention times and fragmentation patterns) of the synthetic standards with those of the original *Limnospira* hydrolysate.

**Figure 4:**
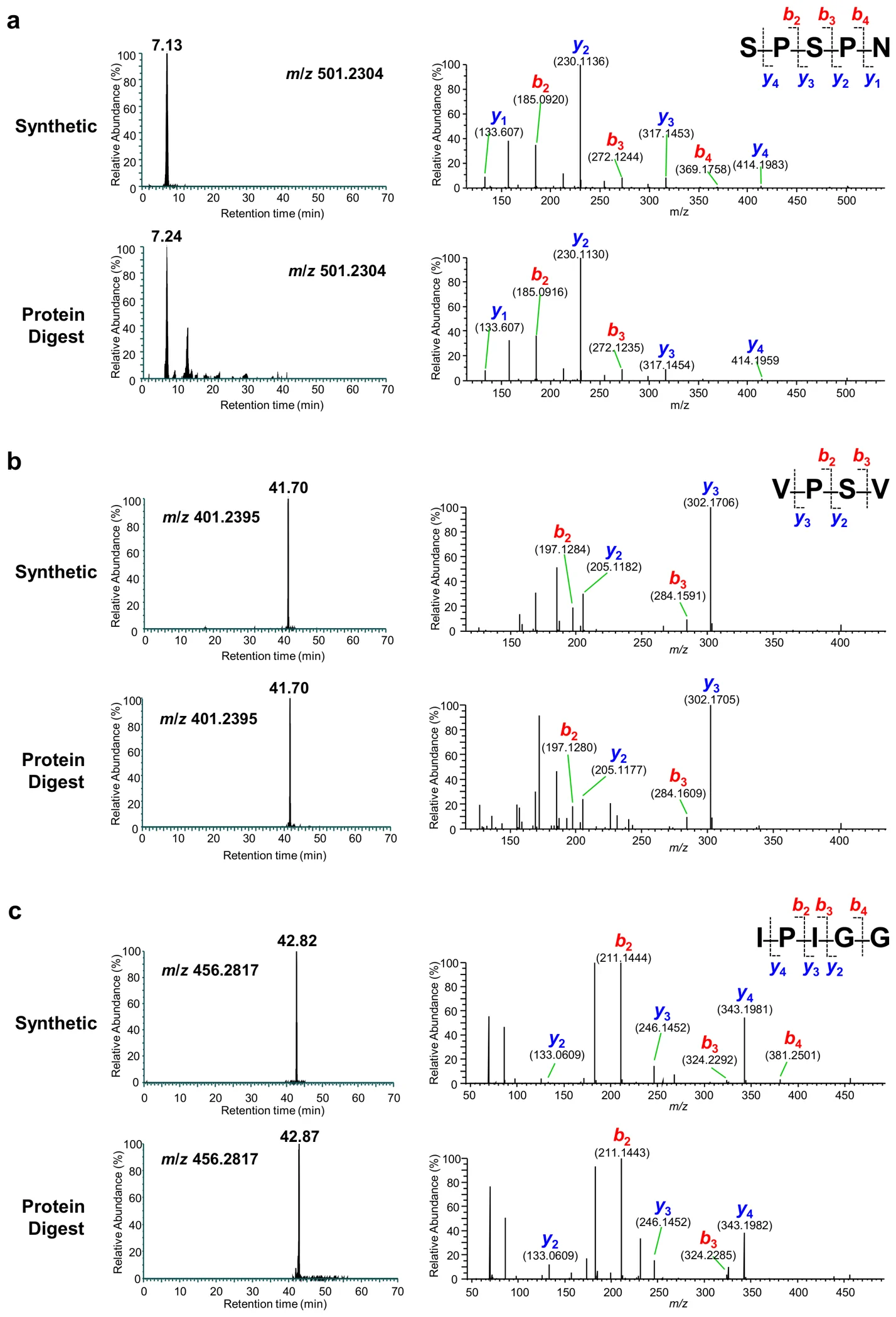
Identification of DPP-IV inhibitory peptides by LC-MS/MS. Extracted ion chromatograms (extracted at the indicated *m*/*z* values; left) and MS/MS spectra (right) of the synthetic peptide standards (upper) and the protein digest (lower) are shown for the three identified peptides: (**a**) SPSPN, (**b**) VPSV, and (**c**) IPIGG. Identification was confirmed by comparing their retention times and fragmentation patterns. In the MS/MS spectra, the corresponding amino acid sequences along with the predicted *b*- and *y*-series ions are illustrated in the upper-right insets. *b*-ions and *y*-ions are highlighted in red and blue, respectively.

Thus, LC-MS/MS was employed to identify the DPP-IV inhibitory peptides present in fractions 2 and 14. A total of 907 peptides were predicted for fraction 2, and 1211 for fraction 14 (a comprehensive list of all 2118 identified sequence candidates is provided in Table S1). Systematic in silico screening of the inhibitor peptide candidates was then performed on all 2118 peptides. This process yielded four peptides from fraction 2 and ten from fraction 14. To focus on the most promising candidates for subsequent validation, the candidates were further filtered based on their abundance in the LC-MS/MS data, selecting the top 50% most abundant peptides from each fraction (i.e., top two from fraction 2 and top five from fraction 14). Consequently, upon combination of the in silico screening and LC-MS/MS results, seven candidate peptides (i.e., APAPQ, SPSPN, VPSV, VPVS, IPGAT, IPIN, and IPIGG) were identified from fractions 2 and 14 (Figure 3). This systematic approach enabled the efficient identification of highly active peptides among many candidates. Subsequently, the candidate peptides were synthesized, and their retention times and mass spectra were compared with those of the digested samples using LC-MS/MS analysis. The results confirmed that only three peptides, namely SPSPN (Ser-Pro-Ser-Pro-Asn), VPSV (Val-Pro-Ser-Val), and IPIGG (Ile-Pro-Ile-Gly-Gly), were present in the digested samples, based on the matching profiles of their major *b*- and *y*-series ions (Figure 4). No distinct peaks corresponding to the other four candidates (APAPQ, VPVS, IPGAT, and IPIN) were observed in the LC-MS/MS data. The detailed mass spectroscopic parameters of the three confirmed peptides, including observed and calculated m/z, mass errors (ppm), sequence coverage, and key matched fragment ions, are summarized in Table 1.

**Figure 5:**
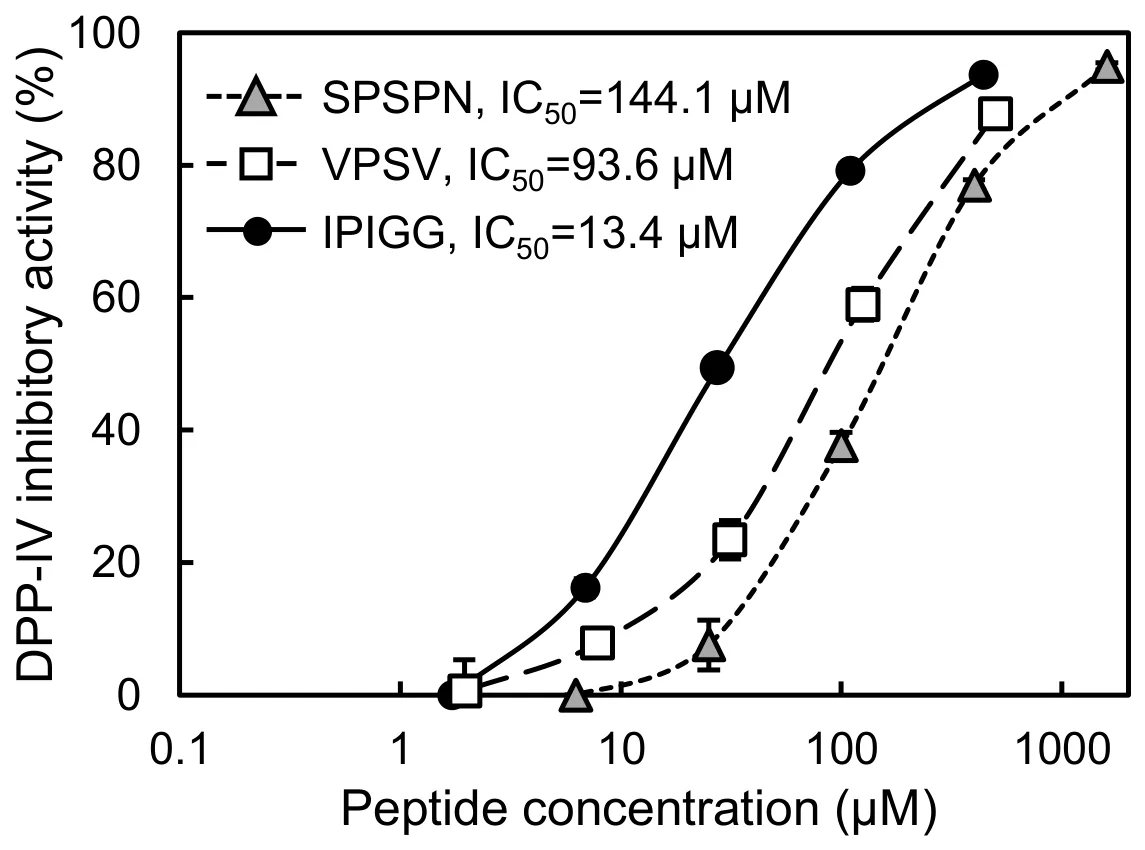
DPP-IV inhibitory activities of the three identified synthetic peptides. Concentration-dependent DPP-IV inhibitory curves for SPSPN, VPSV, and IPIGG. Each value represents the mean ± S.D. (*n* = 3). IC_50_ values for each peptide are shown in the plot.

The DPP-IV inhibitory activities of the three identified peptides were subsequently evaluated. All peptides exhibited concentration-dependent inhibition, with IC_50_ values of 144.1 ± 2.2 μM for SPSPN, 93.6 ± 5.8 μM for VPSV, and 13.4 ± 2.3 μM for IPIGG (Figure 5). According to previous comprehensive reviews [2,8], food-derived DPP-IV inhibitory peptides span a wide IC_50_ range between ~5 μM and >8000 μM. Within this broad spectrum, the sequences identified in the present study demonstrated highly favorable inhibitory potential, considering that a large number of reported sequences exhibit IC_50_ values in the mid-to-high micromolar or even millimolar range. Notably, IPIGG ranks among the highly active sequences, showing potency comparable to well-known milk-derived peptides such as IPI (3.9 μM) and VPL (15.8 μM) [8]. Furthermore, these activities were superior or comparable to those reported in earlier studies on *Limnospira* (*Spirulina*) species. For instance, all three peptides displayed markedly higher DPP-IV inhibitory activity than GPNYASSER (IC_50_ = 358 μM) from *Limnospira* phycocyanins [20], and VPSV and IPIGG exceeded or matched the potency of the most active sequence reported by Hua et al. [18] (RNPFVFAPTLLTVAAR, IC_50_ = 101.4 μM).

To understand the structural basis for these substantial potencies, the sequence features of VPSV and IPIGG were analyzed in relation to the active site of DPP-IV. For VPSV, the N-terminal Val-Pro sequence (occupying the P2 and P1 positions, respectively) effectively accommodates the hydrophobic S2 and S1 pockets of the active site [2], a structural feature shared with potent inhibitors like IPI and VPL [8]. In addition, its overall neutral charge and hydrophobic nature prevent unfavorable electrostatic repulsion within the catalytic cleft. The even higher potency of IPIGG can be attributed to the presence of hydrophobic isoleucine residues at both the N-terminus and the third position (Ile-Pro-Ile-...). Although the substitution of isoleucine with a polar serine residue at the third position in VPSV slightly reduces its binding affinity, the conserved N-terminal Val-Pro sequence and hydrophobic characteristics still provide a strong structural rationale for its high inhibitory activity.

These results confirm that Orientase 22BF effectively releases novel, high-potency DPP-IV inhibitory peptides from *Limnospira* proteins. Although IPIGG exhibited the highest specific activity (quality), the practical application and standardization of the hydrolysate depend not only on peptide potency but also on peptide abundance (quantity). Therefore, quantitative analysis was subsequently performed to determine their yield and assess their practical relevance in the digest.

### 3.4. Quantification of DPP-IV Inhibitory Peptides in Orientase 22BF Digests

Among the three identified sequences, VPSV (IC_50_ = 93.6 ± 5.8 μM) was the sole peptide quantified above the limit of quantification (LOQ), with a content of 64.1 ± 13.0 μg/g dry weight based on *Limnospira* powder. IPIGG (IC_50_ = 13.4 ± 2.3 μM), despite exhibiting higher potent activity, showed a distinct chromatographic peak in the crude digest, but its response fell below the LOQ based on our validated linear calibration range. In contrast, SPSPN (IC_50_ = 144.1 ± 2.2 μM) remained below the limit of detection (LOD) with no detectable signal in the crude digest, being identified only after multi-step ultrafiltration and RP-HPLC enrichment. These low-abundance or non-detection statuses are likely attributable to their extremely low natural occurrence in the hydrolysate as well as matrix-induced ion suppression. While SPSPN and IPIGG do not contribute substantially to the crude extract’s quantitative activity profile, their identification validates our multi-step enrichment approach.

Based on mass-balance considerations, the assay concentration of VPSV in the reaction mixture is calculated to be 0.064 μg/mL (0.16 μM, MW = 400.48), which is less than 0.2% of its individual IC_50_ value (93.6 ± 5.8 μM). This quantitative discrepancy indicates that VPSV alone cannot account for the potent 71% inhibition observed in the crude digest, suggesting that the overall activity relies on synergistic interactions among multiple components within the complex hydrolysate. Instead, VPSV represents a candidate active marker sequence present within the functional hydrolysate fraction. Given its highest abundance among the identified active sequences and consistent batch-to-batch yield across independent preparations, VPSV represents a practical candidate marker sequence for extract quality control, although comprehensive analytical validation will be required for future commercial standardization.

Notably, for food-derived peptides to exert physiological effects, they must remain stable during processing and gastrointestinal transit, while also effectively crossing the intestinal epithelial barrier. Thus, to further evaluate its suitability as a practical marker, VPSV was subjected to thermal and gastrointestinal stability tests, in addition to membrane permeability assays using synthetic peptides.

### 3.5. Stability Evaluation of DPP-IV Inhibitory Peptides During Heating and Gastrointestinal Digestion

**Figure 6:**
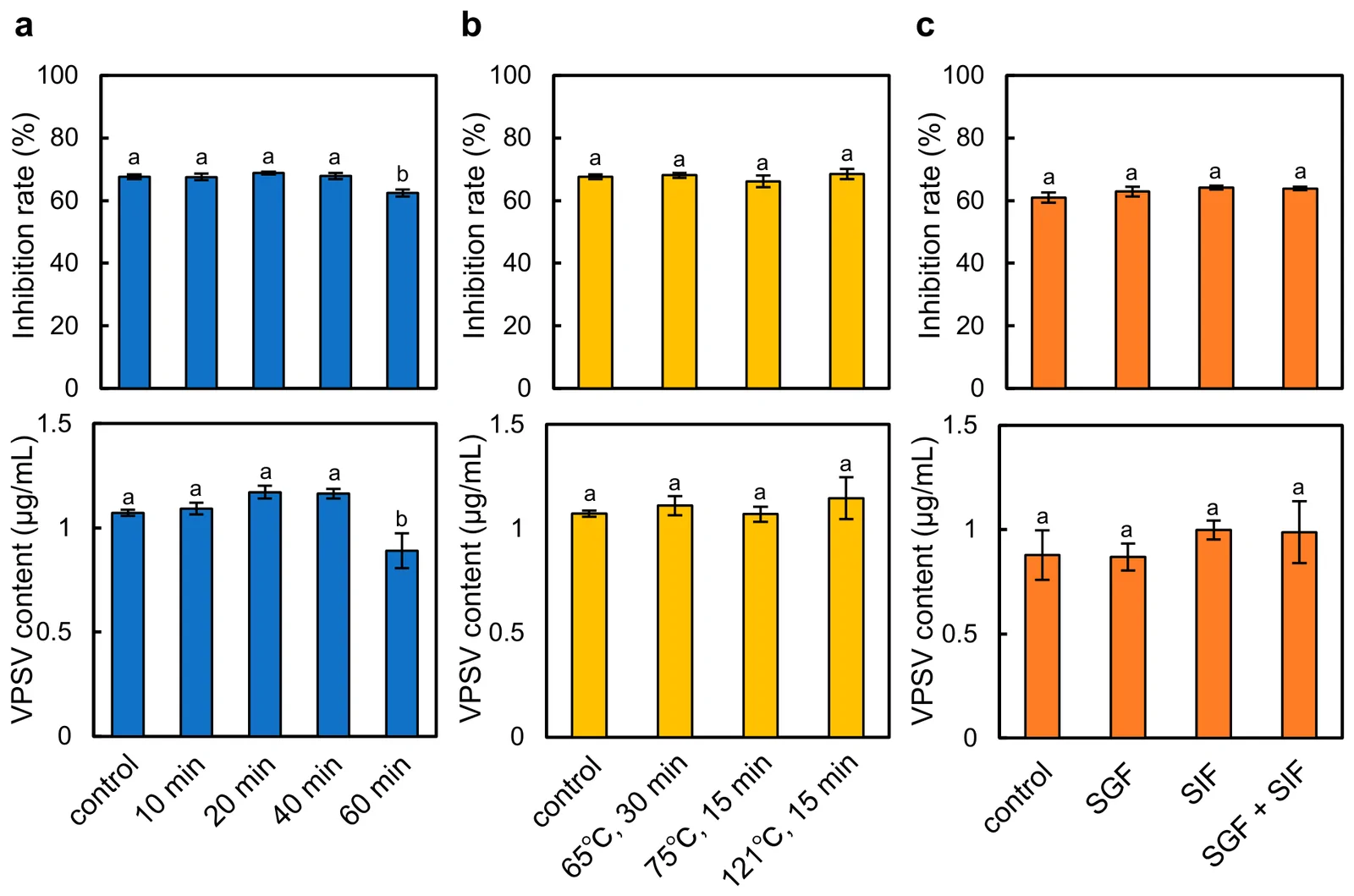
Effects of cooking simulation, heat sterilization, and in vitro gastrointestinal digestion on the DPP-IV inhibitory activity (**upper panels**) and residual content of VPSV (**lower panels**). The peptide was subjected to: (**a**) heating at 100 °C for 60 min to mimic cooking conditions; (**b**) short-term, high-temperature conditions to emulate heat sterilization; and (**c**) SGF and SIF treatments. Each value represents the mean ± S.D. (*n* = 3). Statistical analysis was evaluated by one-way ANOVA followed by Tukey’s honestly significant difference (HSD) post hoc test. Different lowercase letters (a, b) indicate significant differences between treatment groups (*p* < 0.05).

Thermal stability is a key determinant of peptide suitability for food processing applications. To evaluate the thermal stability of VPSV, its DPP-IV inhibitory activity and residual content were compared before and after heat treatment. Treatment at 100 °C to simulate cooking conditions showed no change in the DPP-IV inhibition rate or VPSV content over 40 min. However, after 60 min, the DPP-IV inhibition rate decreased by ~8% and the VPSV content decreased by ~17% (Figure 6a). This parallel decline confirms that the molecular integrity of VPSV directly dictates its bioactivity. Consequently, this suggests that, for products subjected to prolonged heating, process optimization—such as adjusting dosage or shortening the heating time—is necessary to preserve functionality. Conversely, during short-term high-temperature processing to mimic heat sterilization, both the DPP-IV inhibition rate and VPSV content remained similar to those of the unheated sample under all conditions (Figure 6b), suggesting that short-term high-temperature processing successfully maintains functionality.

To ensure effective peptide functionality in vivo, they must resist digestion by gastric and small intestinal proteases. Thus, synthetic VPSV was exposed to simulated gastrointestinal fluids and both the DPP-IV inhibitory activity and remaining peptide content were compared before and after treatment relative to the enzyme-free control. As shown in Figure 6c, VPSV showed no significant decrease in DPP-IV inhibitory activity or content relative to the undigested control, regardless of the digestive fluid employed. These findings demonstrate that VPSV is highly resistant to gastrointestinal digestion under the tested in vitro conditions. In this study, a static digestion model was specifically employed as a target-oriented screening to evaluate the resistance of synthetic VPSV against primary proteolytic cleavage (pepsin, trypsin, and chymotrypsin). Although further studies within complex whole food matrices incorporating updated consensus protocols (e.g., INFOGEST 2.0 with gastric lipase) are required to fully simulate physiological digestion dynamics, these results support the intrinsic structural stability of VPSV as a potential marker for quality control and functional ingredient development.

### 3.6. Evaluation of the Small Intestinal Permeability of DPP-IV Inhibitory Peptides

To exert systemic biological effects after oral administration, peptides must withstand gastrointestinal degradation and permeate the intestinal epithelium into the bloodstream. In the present study, the epithelial transport efficiency of VPSV was evaluated using a differentiated Caco-2 cell monolayer model. Based on the molar amount of intact peptide transported to the basolateral side, the apparent permeability coefficient (P_app_) of VPSV was determined to be 4.02 ± 0.69 × 10^−8^ cm/s. According to established intestinal permeability classification standards [23], a P_app_ value below 1.0 × 10^−6^ cm/s categorizes VPSV as a low-permeability peptide, with less than 1% of the initial donor amount crossing the epithelial barrier under the tested conditions. This low permeability indicates that systemic absorption of the intact peptide is minimal. Consequently, VPSV is more likely to exert its functional DPP-IV inhibitory effects locally within the luminal surface of the small intestine, where membrane-bound DPP-IV is abundantly expressed on the brush-border membrane.

Several methodological limitations of this study should be noted. First, static cell-free enzymatic tests and in vitro digestion models used prior to the transport assay do not fully replicate the dynamic physiological environment of the human gastrointestinal tract; future studies should employ standardized protocols, such as the INFOGEST 2.0 consensus model [24], using complex food matrices. Second, although initial monolayer integrity was confirmed prior to transport by TEER values (>350 Ω·cm^2^), post-transport TEER was not recorded, necessitating more rigorous evaluation of barrier integrity during prolonged transport. Third, specific transport pathways (e.g., paracellular passive diffusion vs. PepT1-mediated active transport) were not delineated using specific inhibitors, nor was the residual intact peptide on the apical side quantified to rule out brush-border enzymatic degradation during transport.

To date, research on the intestinal permeability of *Limnospira*-derived peptides remains extremely limited, particularly for DPP-IV inhibitory peptides, highlighting the novelty of the present findings. In the broader context of peptide absorption, permeability is known to depend heavily on chain length and amino acid sequence. He et al. (2018) reported that two *Limnospira platensis*-derived ACE-inhibitory tripeptides (IQP and VEP) were efficiently transported intact across Caco-2 monolayers (P_app_ = 7.48 ± 0.58 × 10^−6^ and 5.05 ± 0.74 × 10^−6^ cm/s, respectively) via paracellular transport [25]. In contrast, the low P_app_ value observed for VPSV in this study suggests that direct transepithelial flux of the intact tetrapeptide is limited—potentially due to structural constraints or luminal/membrane peptidase degradation—further supporting our hypothesis that VPSV primarily acts locally within the intestinal lumen.

In summary, while VPSV demonstrates functional potential, its direct transepithelial availability as an intact peptide is low. These findings suggest that its biological efficacy after oral ingestion predominantly relies on local luminal action rather than systemic circulation. Further in vivo animal studies and pharmacokinetic evaluations are required to validate its physiological efficacy and therapeutic relevance.

## 4. Conclusions

This study identified novel DPP-IV inhibitory peptides—SPSPN, VPSV, and IPIGG—from a protein hydrolysate derived from the edible microalga *Limnospira* and thoroughly evaluated their functional profiles. Notably, VPSV and IPIGG exhibited high DPP-IV inhibitory activity, comparable or superior to the *Limnospira*-derived peptides reported in earlier studies. In particular, VPSV exhibited robust activity, excellent stability under heat treatment, and resilience in the presence of digestive enzymes under in vitro conditions. Although its transepithelial transport efficiency was limited (P_app_ = 4.02 ± 0.69 × 10^−8^ cm/s), its high enzymatic and digestive stability supports its potential utility, primarily via local action in the intestinal lumen. These results reveal the potential of protease-digested *Limnospira* as a value-added functional food ingredient. Furthermore, combining the systematic screening of multiple proteases with integrated in silico analysis represents an effective strategy for discovering high-functionality peptides and identifying practical marker compounds for quality control. To translate these findings toward clinical and commercial applications, future studies utilizing standardized digestion protocols (such as INFOGEST 2.0) and in vivo animal models will be necessary to clarify functional efficacy and systemic vs. local pharmacokinetic behavior.

## Figures and Tables

**Table 1:** Mass spectrometric identification parameters for the three DPP-IV inhibitory peptides detected in the active hydrolysate fractions. Mass parameters and fragment ions were obtained from the LC-MS/MS analysis of Fractions 2 and 14. Sequence identifications and matched *b*/*y* series ions were further confirmed by comparing retention times and MS/MS spectra with synthetic reference standards, as shown in Figure 4.

Peptide Sequence	Fraction No.	Observed *m*/*z*	Calculated *m*/*z*	Charge (*z*)	Mass Error (ppm)	Matched *b*/*y* ions	Sequence Coverage (%)
SPSPN	Fr. 2	501.2290	501.2304	1	−2.69	*b*_2_, *b*_3_, *y*_1_, *y*_2_, *y*_3_, *y*_4_	100
VPSV	Fr. 14	401.2384	401.2395	1	−2.71	*b*_2_, *b*_3_, *y*_2_, *y*_3_	100
IPIGG	Fr. 14	456.2804	456.2817	1	−2.77	*b*_2_, *b*_3_, *y*_2_, *y*_3_, *y*_4_	100

## Data Availability

The original contributions presented in this study are included in the article/Supplementary Material. Further inquiries can be directed to the corresponding author.

## Supplementary Materials

The following supporting information can be downloaded at: https://www.mdpi.com/article/10.3390/foods15162838/s1, Figure S1: Time-course of food protein hydrolysis by three proteases for determining optimal reaction time; Table S1: List of all candidate peptides identified in high-activity fractions (Fractions 2 and 14) by LC-MS/MS database searching.

## References

[1] International Diabetes Federation (2021). IDF Diabetes Atlas.

[2] Liu R., Cheng J., Wu H. (2019). Discovery of Food-Derived Dipeptidyl Peptidase IV Inhibitory Peptides: A Review. Int. J. Mol. Sci..

[3] Yu D.M.T., Yao T.W., Chowdhury S., Nadvi N.A., Osborne B., Church W.B., McCaughan G.W., Gorrell M.D. (2010). The dipeptidyl peptidase IV family in cancer and cell biology. FEBS J..

[4] Drucker D.J. (2007). Dipeptidyl peptidase-4 inhibition and the treatment of type 2 diabetes: Preclinical biology and mechanisms of action. Diabetes Care.

[5] Gallwitz B. (2007). Review of sitagliptin phosphate: A novel treatment for type 2 diabetes. Vasc. Health Risk Manag..

[6] Deacon C.F., Lebovitz H.E. (2016). Comparative review of dipeptidyl peptidase-4 inhibitors and sulphonylureas. Diabetes Obes. Metab..

[7] Lacroix I.M., Li-Chan E.C. (2014). Overview of food products and dietary constituents with antidiabetic properties and their putative mechanisms of action: A natural approach to complement pharmacotherapy in the management of diabetes. Mol. Nutr. Food Res..

[8] Power O., Nongonierma A.B., Jakeman P., FitzGerald R.J. (2013). Food protein hydrolysates as a source of dipeptidyl peptidase IV inhibitory peptides for the management of type 2 diabetes. Proc. Nutr. Soc..

[9] Nongonierma A.B., FitzGerald R.J. (2013). Dipeptidyl peptidase IV inhibitory and antioxidative properties of milk protein-derived dipeptides and hydrolysates. Peptides.

[10] Nowicka-Krawczyk P., Muhlsteinova R., Hauer T. (2019). Detailed characterization of the *Arthrospira* type species separating commercially grown taxa into the new genus *Limnospira* (Cyanobacteria). Sci. Rep..

[11] Spolaore P., Joannis-Cassan C., Duran E., Isambert A. (2006). Commercial applications of microalgae. J. Biosci. Bioeng..

[12] Ciferri O. (1983). *Spirulina*, the edible microorganism. Microbiol. Rev..

[13] Vo T.-S., Ryu B., Kim S.-K. (2013). Purification of novel anti-inflammatory peptides from enzymatic hydrolysate of the edible microalgal *Spirulina maxima*. J. Funct. Foods.

[14] Sun Y., Chang R., Li Q., Li B. (2015). Isolation and characterization of an antibacterial peptide from protein hydrolysates of *Spirulina platensis*. Eur. Food Res. Technol..

[15] Lin M., Zeng Q., Yang J., Huang J., Zhou M., Lin Z., Zhang Z., Xu F., Wang J.J. (2025). *Spirulina* derived peptides: Inhibition mechanism on pancreatic lipase and impact on lipid-lowering effects of *Caenorhabditis elegans*. Int. J. Biol. Macromol..

[16] Masoumifeshani B., Abedian Kenari A., Sottorff I., Crusemann M., Amiri Moghaddam J. (2025). Identification and Evaluation of Antioxidant and Anti-Aging Peptide Fractions from Enzymatically Hydrolyzed Proteins of *Spirulina platensis* and *Chlorella vulgaris*. Mar. Drugs.

[17] Safitri N.M., Hsu J.L. (2025). Screening of Angiotensin-I Converting Enzyme (ACE) Inhibitory Peptides from Thermolytic Hydrolysate of *Arthrospira platensis*. Mar. Biotechnol..

[18] Hu S., Fan X., Qi P., Zhang X. (2019). Identification of anti-diabetes peptides from *Spirulina platensis*. J. Funct. Foods.

[19] Hannan J.M.A., Ansari P., Azam S., Flatt P.R., Abdel Wahab Y.H.A. (2020). Effects of *Spirulina platensis* on insulin secretion, dipeptidyl peptidase IV activity and both carbohydrate digestion and absorption indicate potential as an adjunctive therapy for diabetes. Br. J. Nutr..

[20] Thongcumsuk B., Woraprayote W., Janyaphisan T., Cheunkar S., Oaew S. (2023). Microencapsulation and Peptide identification of purified bioactive fraction from spirulina protein hydrolysates with dipeptidyl peptidase IV (DPP-IV) inhibitory activity. Food Biosci..

[21] Sugiyama K., Ebisawa M., Yamada M., Nagashima Y., Suzuki H., Maoka T., Takaichi S. (2017). Functional Lycopene Cyclase (CruA) in Cyanobacterium, *Arthrospira platensis* NIES-39, and its Role in Carotenoid Synthesis. Plant Cell Physiol..

[22] Minekus M., Alminger M., Alvito P., Ballance S., Bohn T., Bourlieu C., Carriere F., Boutrou R., Corredig M., Dupont D. (2014). A standardised static in vitro digestion method suitable for food—An international consensus. Food Funct..

[23] Artursson P., Karlsson J. (1991). Correlation between oral drug absorption in humans and apparent drug permeability coefficients in human intestinal epithelial (Caco-2) cells. Biochem. Biophys. Res. Commun..

[24] Brodkorb A., Egger L., Alminger M., Alvito P., Assunção R., Ballance S., Bohn T., Bourlieu-Lacanal C., Boutrou R., Carrière F. (2019). INFOGEST static in vitro simulation of gastrointestinal food digestion. Nat. Protoc..

[25] He Y.Y., Li T.T., Chen J.X., She X.X., Ren D.F., Lu J. (2018). Transport of ACE Inhibitory Peptides Ile-Gln-Pro and Val-Glu-Pro Derived from *Spirulina platensis* Across Caco-2 Monolayers. J. Food Sci..

