# Supplementary material for: Identification and Characterization of Novel DPP-IV Inhibitory Peptides from Limnospira platensis Hydrolysates: Stability and Intestinal Permeability Evaluation
Source: Foods. 2026 Aug 14;15(16):2838. doi: 10.3390/foods15162838 (PMC13512118; doi:10.3390/foods15162838)
Supplement: Supplementary file 1 [file foods-15-02838-s001.zip › Supplementary Information_Figure S1.pdf]

Supplementary Information

”Identification and Characterization of Novel DPP-IV Inhibitory Peptides from Microalgae A Hydrolysates: Stability and Intestinal Permeability Evaluation”

Kota Ebato, Haruka Kobayashi, Hiroaki Tsutsumi, Yoko Iijima, and Kenjiro Sugiyama

Supplementary Methods

Time-Course Protein Hydrolysis and Ninhydrin Assay

Hydrolysis of Limnospira platensis (10 mg/mL) was performed using three distinct proteases at 55 °C and pH 8.0: (a) Orientase 22BF, (b) Orientase OP, and (c) Papain. To determine the time required for complete hydrolysis, aliquots were sampled at designated time points (0, 1, 2, 4, 6, and 8 h), and the reactions were immediately terminated by heating at 95 °C for 10 min. The supernatant was diluted appropriately with distilled water.

Free amino group content was determined using the ninhydrin assay. Briefly, add 4 g of ninhydrin to 150 mL of ethylene glycol and stir thoroughly. After stirring, add 50 mL of 4 N sodium acetate buffer and stir again (Solution A). Add 5 mL of ethylene glycol to 500 mg of stannous chloride and stir thoroughly (Solution B). To 200 µL of each protease digest, 1 mL of ninhydrin solution (Solution A: Solution B = 40:11) was added; the mixture was vortexed and then heated at 80°C for 30 minutes. The mixture was rapidly cooled in ice water for 5 minutes, left to stand at room temperature for 5 minutes, and then the absorbance at 570 nm was measured using a spectrophotometer (BioSpectrometer, Eppendorf).

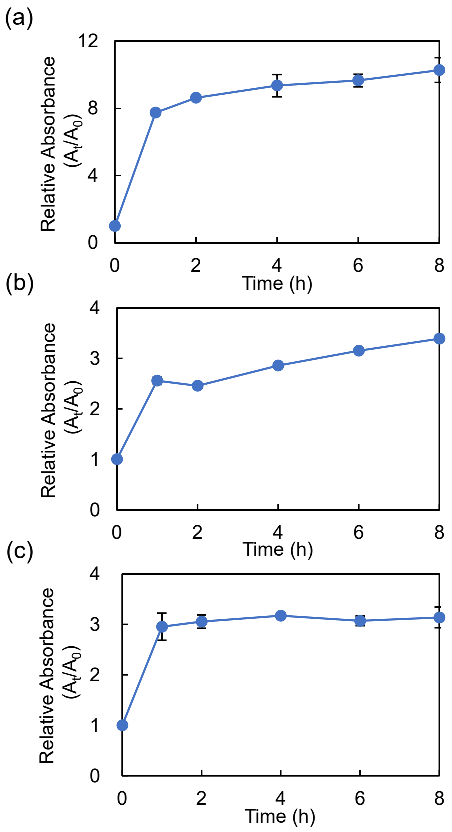

Figure S1. Time-course of food protein hydrolysis by three proteases for determining optimal reaction time.

Hydrolysis of *Limnospira platensis* (10 mg/mL) was performed using three distinct proteases: (a) Orientase 22BF, (b) Orientase OP, and (c) Papain. Data are presented as relative absorbance (At / A0), normalized to the initial absorbance at time 0 (A0 = 1.0). Each point represents the mean ± S.D. (n = 3).

Although the hydrolysis reaction reached a plateau within 1 h for all proteases, a reaction time of 4 h was selected for subsequent experiments to ensure complete and highly reproducible hydrolysis.
