# Supplementary material for: Identification and Characterization of Novel DPP-IV Inhibitory Peptides from Limnospira platensis Hydrolysates: Stability and Intestinal Permeability Evaluation
Source: Foods. 2026 Aug 14;15(16):2838. doi: 10.3390/foods15162838 (PMC13512118; doi:10.3390/foods15162838)
Supplement: Supplementary file 1 [file foods-15-02838-s001.zip › Supplementary Information_Table S1.pdf]

## Supplementary Information

"Identification and Characterization of Novel DPP-IV Inhibitory Peptides from Microalgae A Hydrolysates: Stability and Intestinal Permeability Evaluation"
Kota Ebato, Haruka Kobayashi, Hiroaki Tsutsumi, Yoko Iijima, and Kenjiro Sugiyama

**Table S1. List of all candidate peptides identified in high-activity fractions (Fractions 2 and 14) by LC-MS/MS database searching.**

| Sequence | Sequence Length | Modifications | calculated MH+ [Da] | observed *m/z* [Da] | ΔM [ppm] | Charge | Abundance | Xcorr | Confidence |
|---|---|---|---|---|---|---|---|---|---|
| **Fraction no. 2** | | | | | | | | | |
| SGDAAVE | 7 | | 648.28351 | 648.28186 | -2.55 | 1 | 643294.5625 | 1.65 | High |
| SPSPGG | 6 | | 501.23035 | 501.229 | -2.69 | 1 | 357442.0625 | 1.64 | High |
| SPSPN | 5 | | 501.23035 | 501.229 | -2.69 | 1 | 357442.0625 | 1.64 | High |
| TNPTTGG | 7 | | 647.2995 | 647.29767 | -2.82 | 1 | 1541432 | 1.58 | High |
| TNPTTN | 6 | | 647.2995 | 647.29767 | -2.82 | 1 | 1541432 | 1.58 | High |
| VVGEE | 5 | | 532.26132 | 532.26007 | -2.34 | 1 | 673475.4375 | 1.56 | High |
| SVDDADSH | 8 | | 845.32717 | 423.16702 | -0.47 | 2 | 287535.375 | 1.54 | Medium |
| DAGKEM | 6 | 1xOxidation [M6] | 666.27632 | 666.27405 | -3.41 | 1 | 546685.4375 | 1.51 | High |
| TDEGPSKE | 8 | | 862.37887 | 431.69193 | -2.66 | 2 | 361099.2813 | 1.45 | Medium |
| GDAAVE | 6 | | 561.25148 | 561.25031 | -2.1 | 1 | 719213.3125 | 1.42 | High |
| EESERVE | 7 | | 877.38977 | 439.19745 | -2.45 | 2 | 304062.9375 | 1.42 | Medium |
| EEVSREE | 7 | | 877.38977 | 439.19745 | -2.45 | 2 | 304062.9375 | 1.4 | Medium |
| EGSAVT | 6 | | 563.26713 | 563.26563 | -2.68 | 1 | 948410.8125 | 1.4 | High |
| AGGAAPG | 7 | | 500.24634 | 500.24509 | -2.5 | 1 | 279057.3125 | 1.38 | High |
| AAPGAE | 6 | | 515.246 | 515.24493 | -2.07 | 1 | 795734 | 1.34 | High |
| ETIQ | 4 | | 490.25075 | 490.24951 | -2.53 | 1 | 2756744.5 | 1.33 | High |
| ETIAG | 5 | | 490.25075 | 490.24951 | -2.53 | 1 | 2756744.5 | 1.33 | High |
| ETIGA | 5 | | 490.25075 | 490.24951 | -2.53 | 1 | 2756744.5 | 1.33 | High |
| APATGGA | 7 | | 544.27255 | 544.27216 | -0.73 | 1 | 172350.5938 | 1.33 | High |
| TEAGVE | 6 | | 605.2777 | 605.27606 | -2.7 | 1 | 82160.39063 | 1.32 | High |
| GEVAE | 5 | | 504.23002 | 504.22879 | -2.44 | 1 | 206772.5469 | 1.32 | High |
| DAVAE | 5 | | 504.23002 | 504.22879 | -2.44 | 1 | 206772.5469 | 1.32 | High |
| ADVAE | 5 | | 504.23002 | 504.22879 | -2.44 | 1 | 206772.5469 | 1.32 | High |
| EGVAE | 5 | | 504.23002 | 504.22879 | -2.44 | 1 | 206772.5469 | 1.32 | High |
| DADTM | 5 | | 552.197 | 552.19592 | -1.96 | 1 | 419811.5 | 1.29 | High |
| SANPDQA | 7 | | 702.30531 | 702.30389 | -2.01 | 1 | 391221.9961 | 1.29 | High |
| EGIQ | 4 | | 446.22454 | 446.22345 | -2.44 | 1 | 2449420 | 1.29 | High |
| EGIGA | 5 | | 446.22454 | 446.22345 | -2.44 | 1 | 2449420 | 1.29 | High |
| KGEM | 4 | | 464.21735 | 464.21628 | -2.3 | 1 | 1875808.625 | 1.28 | High |
| SENPTGS | 7 | | 691.28932 | 691.28815 | -1.7 | 1 | 248527.9063 | 1.25 | High |
| DTNPSTG | 7 | | 691.28932 | 691.28815 | -1.7 | 1 | 248527.9063 | 1.25 | High |
| EGGTM | 5 | | 494.19152 | 494.1907 | -1.66 | 1 | 319530.4063 | 1.24 | High |
| GTPGDAQ | 7 | | 645.28385 | 645.28204 | -2.79 | 1 | 237688.2813 | 1.24 | High |
| DSGTIS | 6 | | 579.26205 | 579.26086 | -2.04 | 1 | 1002559.25 | 1.23 | High |
| SDGTIS | 6 | | 579.26205 | 579.26086 | -2.04 | 1 | 1002559.25 | 1.23 | High |
| REQQNPE | 7 | | 900.41698 | 450.71075 | -3.06 | 2 | 266575.4063 | 1.23 | Medium |
| KQVTVRILK | 9 | | 1084.72009 | 362.24692 | 5.63 | 3 | | 1.22 | Medium |
| KGEGME | 6 | | 650.2814 | 650.27966 | -2.67 | 1 | 17215648.88 | 1.22 | High |
| DIGDSD | 6 | | 621.23623 | 621.23444 | -2.88 | 1 | 308777.8438 | 1.21 | High |
| DAGKEM | 6 | | 650.2814 | 650.27948 | -2.96 | 1 | 10008949 | 1.21 | High |
| AEVGGT | 6 | | 533.25657 | 533.25562 | -1.79 | 1 | 878292.5 | 1.21 | High |
| AEVGAS | 6 | | 533.25657 | 533.25562 | -1.79 | 1 | 878292.5 | 1.21 | High |
| KGGQML | 6 | 1xOxidation [M5] | 649.33377 | 649.33838 | 7.09 | 1 | 7610981 | 1.21 | High |
| NEGENL | 6 | | 675.29441 | 675.29254 | -2.77 | 1 | 1545673.75 | 1.2 | High |
| AVSGD | 5 | | 448.2038 | 448.20276 | -2.33 | 1 | 1094893.875 | 1.2 | High |
| VASGD | 5 | | 448.2038 | 448.20276 | -2.33 | 1 | 1094893.875 | 1.2 | High |
| GISGD | 5 | | 448.2038 | 448.20276 | -2.33 | 1 | 1153584.309 | 1.2 | High |
| IGSGD | 5 | | 448.2038 | 448.20276 | -2.33 | 1 | 1153584.309 | 1.2 | High |
| DGETIN | 6 | | 648.28351 | 648.28186 | -2.55 | 1 | | 1.2 | High |
| EGVAN | 5 | | 489.23035 | 489.22937 | -2.01 | 1 | 210076.625 | 1.19 | Medium |
| EAIE | 4 | | 461.2242 | 461.22311 | -2.37 | 1 | 2039038.133 | 1.19 | Medium |
| QSVDQQ | 6 | | 704.32096 | 704.31946 | -2.13 | 1 | 168377.1875 | 1.19 | Medium |
| VNDPA | 5 | | 515.246 | 515.24457 | -2.79 | 1 | 8363194.5 | 1.19 | Medium |
| VNDAP | 5 | | 515.246 | 515.24457 | -2.79 | 1 | 8363194.5 | 1.19 | Medium |
| NVDPA | 5 | | 515.246 | 515.24457 | -2.79 | 1 | 8363194.5 | 1.19 | Medium |
| DAGIGSM | 7 | | 650.2814 | 325.64343 | -2.79 | 2 | 7131770 | 1.19 | Medium |
| AAPPQ | 5 | | 483.25617 | 483.25488 | -2.67 | 1 | 1744509.5 | 1.18 | Medium |
| APAPAG | 6 | | 483.25617 | 483.25488 | -2.67 | 1 | 1744509.5 | 1.18 | Medium |
| APAPQ | 5 | | 483.25617 | 483.25488 | -2.67 | 1 | 1744509.5 | 1.18 | Medium |
| EGGLN | 5 | | 489.23035 | 489.22882 | -3.13 | 1 | 4487008.5 | 1.18 | Medium |
| SETDV | 5 | | 550.2355 | 550.23413 | -2.48 | 1 | 597630.75 | 1.17 | Medium |
| DTTDV | 5 | | 550.2355 | 550.23413 | -2.48 | 1 | 597630.75 | 1.17 | Medium |
| SPDPA | 5 | | 486.21945 | 486.21854 | -1.89 | 1 | 461636.1875 | 1.17 | Medium |
| MAGE | 4 | | 407.1595 | 407.1586 | -2.2 | 1 | 149350.5938 | 1.17 | Medium |
| SPDAP | 5 | | 486.21945 | 486.21854 | -1.89 | 1 | 461636.1875 | 1.17 | Medium |
| SPVE | 4 | | 431.21364 | 431.21274 | -2.09 | 1 | 279064.8438 | 1.17 | Medium |
| EGGVGA | 6 | | 489.23035 | 489.22922 | -2.32 | 1 | 4487008.5 | 1.17 | Medium |
| SQMVAE | 6 | 1xOxidation [M3] | 680.29197 | 680.29047 | -2.21 | 1 | 416105.1055 | 1.17 | Medium |
| EGVGAG | 6 | | 489.23035 | 489.22937 | -2.01 | 1 | 210076.625 | 1.16 | Medium |
| EGVGQ | 5 | | 489.23035 | 489.22937 | -2.01 | 1 | 210076.625 | 1.16 | Medium |
| QSIE | 4 | | 476.2351 | 476.23413 | -2.04 | 1 | 1009510.652 | 1.16 | Medium |
| AGSLE | 5 | | 476.2351 | 476.23413 | -2.04 | 1 | 973590.125 | 1.16 | Medium |
| GASLE | 5 | | 476.2351 | 476.23413 | -2.04 | 1 | 1009510.652 | 1.16 | Medium |
| EIITDGNLGKQIESVCRA | 18 | | 1946.00115 | 649.3385 | -0.1 | 3 | | 1.15 | Medium |
| GSSPSGA | 7 | | 562.24673 | 562.24524 | -2.65 | 1 | 1075398.75 | 1.14 | Medium |
| GTPTDQ | 6 | | 618.27295 | 618.2713 | -2.66 | 1 | 4150965.25 | 1.14 | Medium |

| | | | | | | | | | |
|---|---|---|---|---|---|---|---|---|---|
| EDNLN | 5 | | 604.2573 | 604.25604 | -2.07 | 1 | 2129810.113 | 1.14 | Medium |
| DENIN | 5 | | 604.2573 | 604.25604 | -2.07 | 1 | 2129810.113 | 1.14 | Medium |
| GEEMT | 5 | | 566.21265 | 566.21118 | -2.6 | 1 | 370844.7813 | 1.14 | Medium |
| EGEMT | 5 | | 566.21265 | 566.21118 | -2.6 | 1 | 370844.7813 | 1.14 | Medium |
| DAEMT | 5 | | 566.21265 | 566.21118 | -2.6 | 1 | 370844.7813 | 1.14 | Medium |
| YAAD | 4 | | 439.18234 | 439.18124 | -2.5 | 1 | 2737860.25 | 1.14 | Medium |
| AEAI | 4 | | 403.21873 | 403.21756 | -2.89 | 1 | 9513513.078 | 1.14 | Medium |
| EIGE | 4 | | 447.20855 | 447.20816 | -0.88 | 1 | 546781.5391 | 1.14 | Medium |
| EAAI | 4 | | 403.21873 | 403.21756 | -2.89 | 1 | 9513513.078 | 1.13 | Medium |
| ADQID | 5 | | 561.25148 | 561.24994 | -2.75 | 1 | 558846.5625 | 1.13 | Medium |
| DAQID | 5 | | 561.25148 | 561.24994 | -2.75 | 1 | 558846.5625 | 1.13 | Medium |
| EGQID | 5 | | 561.25148 | 561.24994 | -2.75 | 1 | 558846.5625 | 1.13 | Medium |
| CHGTR | 5 | | 573.25619 | 573.26123 | 8.79 | 1 | 980554 | 1.12 | Medium |
| NTGPM | 5 | 1xOxidation [M5] | 535.21807 | 535.2168 | -2.39 | 1 | 33852163 | 1.12 | Medium |
| GGTGPM | 6 | 1xOxidation [M6] | 535.21807 | 535.2168 | -2.39 | 1 | 33852163 | 1.12 | Medium |
| KDDENIN | 7 | | 847.3792 | 424.19241 | -1.95 | 2 | 384523.9063 | 1.12 | Medium |
| TTDNTVS | 7 | | 737.33119 | 737.32983 | -1.84 | 1 | 4502794.594 | 1.12 | Medium |
| DANIN | 5 | | 546.25182 | 546.25031 | -2.77 | 1 | 345457.5938 | 1.11 | Medium |
| GENIN | 5 | | 546.25182 | 546.25031 | -2.77 | 1 | 345457.5938 | 1.11 | Medium |
| ADNLN | 5 | | 546.25182 | 546.25031 | -2.77 | 1 | 345457.5938 | 1.11 | Medium |
| PAGAT | 5 | | 416.21397 | 416.2128 | -2.82 | 1 | 149665.9844 | 1.11 | Medium |
| APGAT | 5 | | 416.21397 | 416.2128 | -2.82 | 1 | 149665.9844 | 1.11 | Medium |
| DAGGLGG | 7 | | 546.25182 | 546.25031 | -2.77 | 1 | 345457.5938 | 1.11 | Medium |
| PGTGDAQ | 7 | | 645.28385 | 645.28137 | -3.83 | 1 | | 1.11 | Medium |
| AGSGDME | 7 | | 666.23993 | 666.23859 | -2.02 | 1 | 138918.4219 | 1.11 | Medium |
| TCAGNP | 6 | | 562.22897 | 562.22736 | -2.88 | 1 | 36210684 | 1.11 | Medium |
| MQGPN | 5 | 1xOxidation [M1] | 562.22897 | 562.22736 | -2.88 | 1 | 36210684 | 1.11 | Medium |
| QMGNP | 5 | 1xOxidation [M2] | 562.22897 | 562.22736 | -2.88 | 1 | 36210684 | 1.11 | Medium |
| GAAID | 5 | | 446.22454 | 446.22357 | -2.17 | 1 | 11295279 | 1.1 | Medium |
| QAID | 4 | | 446.22454 | 446.22357 | -2.17 | 1 | 11295279 | 1.1 | Medium |
| AGAID | 5 | | 446.22454 | 446.22357 | -2.17 | 1 | 11295279 | 1.1 | Medium |
| AAGID | 5 | | 446.22454 | 446.22357 | -2.17 | 1 | 11295279 | 1.1 | Medium |
| AQID | 4 | | 446.22454 | 446.22357 | -2.17 | 1 | 11295279 | 1.1 | Medium |
| GPAAT | 5 | | 416.21397 | 416.2128 | -2.82 | 1 | 149665.9844 | 1.09 | Medium |
| PGAAT | 5 | | 416.21397 | 416.2128 | -2.82 | 1 | 149665.9844 | 1.09 | Medium |
| VPEAG | 5 | | 472.24019 | 472.23895 | -2.62 | 1 | 128770.5469 | 1.09 | Medium |
| VPEQ | 4 | | 472.24019 | 472.23895 | -2.62 | 1 | 128770.5469 | 1.09 | Medium |
| EGITS | 5 | | 506.24567 | 506.24438 | -2.54 | 1 | 387881.2813 | 1.09 | Medium |
| ADITS | 5 | | 506.24567 | 506.24438 | -2.54 | 1 | 387881.2813 | 1.09 | Medium |
| GEITS | 5 | | 506.24567 | 506.24438 | -2.54 | 1 | 387881.2813 | 1.09 | Medium |
| DAITS | 5 | | 506.24567 | 506.24438 | -2.54 | 1 | 387881.2813 | 1.09 | Medium |
| VVSAG | 5 | | 432.24527 | 432.24414 | -2.62 | 1 | 3878152.5 | 1.09 | Medium |
| VVSQ | 4 | | 432.24527 | 432.24414 | -2.62 | 1 | 3878152.5 | 1.09 | Medium |
| VVSGA | 5 | | 432.24527 | 432.24414 | -2.62 | 1 | 3878152.5 | 1.09 | Medium |
| EQPEAR | 6 | | 729.35259 | 365.17905 | -2.44 | 2 | 103654.9063 | 1.09 | Medium |
| AEVAE | 5 | | 518.24567 | 518.24432 | -2.6 | 1 | 79343.45313 | 1.08 | Medium |
| EAIGA | 5 | | 460.24019 | 460.23932 | -1.89 | 1 | 853665.6875 | 1.08 | Medium |
| EAIQ | 4 | | 460.24019 | 460.23932 | -1.89 | 1 | 853665.6875 | 1.08 | Medium |
| EAIAG | 5 | | 460.24019 | 460.23932 | -1.89 | 1 | 834349.5625 | 1.08 | Medium |
| EAVAE | 5 | | 518.24567 | 518.24432 | -2.6 | 1 | 79343.45313 | 1.08 | Medium |
| AIDSQ | 5 | | 533.25657 | 533.25531 | -2.36 | 1 | 267870.6563 | 1.08 | Medium |
| AIDSAG | 6 | | 533.25657 | 533.25531 | -2.36 | 1 | 267870.6563 | 1.08 | Medium |
| AIDSGA | 6 | | 533.25657 | 533.25531 | -2.36 | 1 | 267870.6563 | 1.08 | Medium |
| LADSAG | 6 | | 533.25657 | 533.25531 | -2.36 | 1 | 267870.6563 | 1.08 | Medium |
| IADSQ | 5 | | 533.25657 | 533.25531 | -2.36 | 1 | 267870.6563 | 1.08 | Medium |
| GEGTM | 5 | | 494.19152 | 494.19052 | -2.03 | 1 | | 1.08 | Medium |
| GEGIE | 5 | | 504.23002 | 504.22888 | -2.25 | 1 | 1056864.176 | 1.08 | Medium |
| EGGLE | 5 | | 504.23002 | 504.22888 | -2.25 | 1 | 913488.1406 | 1.08 | Medium |
| DAGIE | 5 | | 504.23002 | 504.22888 | -2.25 | 1 | 913488.1406 | 1.08 | Medium |
| AEAPA | 5 | | 458.22454 | 458.2236 | -2.04 | 1 | 1433701.125 | 1.08 | Medium |
| EAAPA | 5 | | 458.22454 | 458.2236 | -2.04 | 1 | 1433701.125 | 1.08 | Medium |
| AEAAP | 5 | | 458.22454 | 458.2236 | -2.04 | 1 | 1433701.125 | 1.08 | Medium |
| EAAAP | 5 | | 458.22454 | 458.2236 | -2.04 | 1 | 1433701.125 | 1.08 | Medium |
| EAIN | 4 | | 446.22454 | 446.22321 | -2.99 | 1 | 2772867.609 | 1.08 | Medium |
| EAIGG | 5 | | 446.22454 | 446.22321 | -2.99 | 1 | 2772867.609 | 1.08 | Medium |
| AEIN | 4 | | 446.22454 | 446.22321 | -2.99 | 1 | 2678526.75 | 1.08 | Medium |
| ESGGAI | 6 | | 533.25657 | 533.25531 | -2.36 | 1 | 267870.6563 | 1.07 | Medium |
| VAAAE | 5 | | 460.24019 | 460.23862 | -3.42 | 1 | 126475.9727 | 1.07 | Medium |
| LGAAE | 5 | | 460.24019 | 460.23862 | -3.42 | 1 | 126475.9727 | 1.07 | Medium |
| GIAAE | 5 | | 460.24019 | 460.23862 | -3.42 | 1 | 126475.9727 | 1.07 | Medium |
| AVAAE | 5 | | 460.24019 | 460.23862 | -3.42 | 1 | 126475.9727 | 1.07 | Medium |
| KDQIE | 5 | | 632.32498 | 632.32227 | -4.3 | 1 | 276875.9688 | 1.07 | Medium |
| EDNVGA | 6 | | 604.2573 | 604.25641 | -1.47 | 1 | 2080173.828 | 1.06 | Medium |
| DNAAPA | 6 | | 558.25182 | 558.25079 | -1.83 | 1 | 618764.1875 | 1.06 | Medium |
| ADDAL | 5 | | 504.23002 | 504.22891 | -2.19 | 1 | 531424.3125 | 1.06 | Medium |
| GEDAL | 5 | | 504.23002 | 504.22891 | -2.19 | 1 | 531424.3125 | 1.06 | Medium |
| EGDAI | 5 | | 504.23002 | 504.22891 | -2.19 | 1 | 531424.3125 | 1.06 | Medium |
| GESI | 4 | | 405.19799 | 405.1969 | -2.69 | 1 | 5989520.266 | 1.06 | Medium |
| DASI | 4 | | 405.19799 | 405.1969 | -2.69 | 1 | 5473024.248 | 1.06 | Medium |
| ADSI | 4 | | 405.19799 | 405.1969 | -2.69 | 1 | 5284104.482 | 1.06 | Medium |
| SGEDAAEA | 8 | | 749.2948 | 749.2934 | -1.88 | 1 | 224219.7813 | 1.06 | Medium |
| EGSI | 4 | | 405.19799 | 405.1969 | -2.69 | 1 | 5989520.266 | 1.06 | Medium |
| MAAP | 4 | 1xOxidation [M1] | 405.18023 | 405.17944 | -1.95 | 1 | 174562.5313 | 1.05 | Medium |

| | | | | | | | | | |
|---|---|---|---|---|---|---|---|---|---|
| MAPA | 4 | 1xOxidation [M1] | 405.18023 | 405.17944 | -1.95 | 1 | 174562.5313 | 1.05 | Medium |
| GSGDME | 6 | | 595.20282 | 595.20215 | -1.12 | 1 | 89577.40625 | 1.05 | Medium |
| GDSVVE | 6 | | 605.2777 | 605.27655 | -1.89 | 1 | 216521.0781 | 1.05 | Medium |
| NEGNVG | 6 | | 589.25763 | 589.25647 | -1.97 | 1 | 222098.6094 | 1.05 | Medium |
| DAGGNVG | 7 | | 589.25763 | 589.25647 | -1.97 | 1 | 222098.6094 | 1.05 | Medium |
| IDSGN | 5 | | 505.22527 | 505.22403 | -2.45 | 1 | 478301.75 | 1.05 | Medium |
| DISGN | 5 | | 505.22527 | 505.22403 | -2.45 | 1 | 478301.75 | 1.05 | Medium |
| DEGAI | 5 | | 504.23002 | 504.22891 | -2.19 | 1 | 531424.3125 | 1.05 | Medium |
| EDGAI | 5 | | 504.23002 | 504.22891 | -2.19 | 1 | 608476.9609 | 1.05 | Medium |
| ESLGA | 5 | | 476.2351 | 476.23444 | -1.4 | 1 | 1792781.094 | 1.04 | Medium |
| ESIAG | 5 | | 476.2351 | 476.23444 | -1.4 | 1 | 1792781.094 | 1.04 | Medium |
| ESIQ | 4 | | 476.2351 | 476.23444 | -1.4 | 1 | 1792781.094 | 1.04 | Medium |
| ADIT | 4 | | 419.21364 | 419.21277 | -2.08 | 1 | 3164471 | 1.04 | Medium |
| GEIT | 4 | | 419.21364 | 419.21277 | -2.08 | 1 | 3164471 | 1.04 | Medium |
| DAIT | 4 | | 419.21364 | 419.21277 | -2.08 | 1 | 3164471 | 1.04 | Medium |
| SDIE | 4 | | 463.20347 | 463.20258 | -1.93 | 1 | 383435.7188 | 1.04 | Medium |
| DSIE | 4 | | 463.20347 | 463.20258 | -1.93 | 1 | 383435.7188 | 1.04 | Medium |
| EGIT | 4 | | 419.21364 | 419.21277 | -2.08 | 1 | 3164471 | 1.04 | Medium |
| KGEMKG | 6 | | 649.33377 | 649.33826 | 6.91 | 1 | | 1.04 | Medium |
| AETI | 4 | | 433.22929 | 433.22827 | -2.35 | 1 | 403576.2969 | 1.04 | Medium |
| QRIVLMMNEYN | 11 | 1xOxidation [M] | 1426.68173 | 476.23343 | 2.8 | 3 | 308740.2813 | 1.04 | Medium |
| EATI | 4 | | 433.22929 | 433.22827 | -2.35 | 1 | 403576.2969 | 1.04 | Medium |
| EGGIG | 5 | | 432.20889 | 432.20767 | -2.82 | 1 | 708440.0938 | 1.04 | Medium |
| EGGAV | 5 | | 432.20889 | 432.20767 | -2.82 | 1 | 2112586.656 | 1.04 | Medium |
| EGGGL | 5 | | 432.20889 | 432.20767 | -2.82 | 1 | 859066.5313 | 1.04 | Medium |
| ENKIE | 5 | | 632.32498 | 632.32227 | -4.3 | 1 | 276875.9688 | 1.04 | Medium |
| QPAAE | 5 | | 515.246 | 515.245 | -1.96 | 1 | | 1.03 | Medium |
| EGSLGA | 6 | | 533.25657 | 533.25555 | -1.9 | 1 | 76086.60156 | 1.03 | Medium |
| EGSIAG | 6 | | 533.25657 | 533.25555 | -1.9 | 1 | 76086.60156 | 1.03 | Medium |
| EGSIQ | 5 | | 533.25657 | 533.25555 | -1.9 | 1 | 76086.60156 | 1.03 | Medium |
| GATVE | 5 | | 476.2351 | 476.23404 | -2.24 | 1 | 673987.7969 | 1.03 | Medium |
| QTVE | 4 | | 476.2351 | 476.23404 | -2.24 | 1 | 673987.7969 | 1.03 | Medium |
| AGTVE | 5 | | 476.2351 | 476.23404 | -2.24 | 1 | 673987.7969 | 1.03 | Medium |
| GTGASPQ | 7 | | 617.28893 | 617.28735 | -2.55 | 1 | 215856.2656 | 1.03 | Medium |
| DGANIE | 6 | | 618.27295 | 618.26874 | -6.81 | 1 | 118289.0469 | 1.02 | Medium |
| AAVAE | 5 | | 460.24019 | 460.23862 | -3.42 | 1 | 58983.78516 | 1.02 | Medium |
| DVTGT | 5 | | 492.23002 | 492.22913 | -1.81 | 1 | 625662.1875 | 1.02 | Medium |
| DNIE | 4 | | 490.21437 | 490.2135 | -1.77 | 1 | 159368.5469 | 1.02 | Medium |
| NDIE | 4 | | 490.21437 | 490.2135 | -1.77 | 1 | 159368.5469 | 1.02 | Medium |
| GGDIE | 5 | | 490.21437 | 490.2135 | -1.77 | 1 | 159368.5469 | 1.02 | Medium |
| EAMRRLQ | 7 | 1xOxidation [M3] | 919.47781 | 460.24112 | -3.1 | 2 | 336591.3359 | 1.02 | Medium |
| DGGLE | 5 | | 490.21437 | 490.2135 | -1.77 | 1 | 159368.5469 | 1.02 | Medium |
| GDGIE | 5 | | 490.21437 | 490.2135 | -1.77 | 1 | 159368.5469 | 1.02 | Medium |
| SEGGVEN | 7 | | 691.28932 | 691.28827 | -1.53 | 1 | | 1.02 | Medium |
| VGDPN | 5 | | 501.23035 | 501.22919 | -2.33 | 1 | | 1.01 | Medium |
| GVDPN | 5 | | 501.23035 | 501.22919 | -2.33 | 1 | | 1.01 | Medium |
| VAESN | 5 | | 519.24092 | 519.23944 | -2.84 | 1 | 874010.125 | 1.01 | Medium |
| ESGGDNL | 7 | | 691.28932 | 691.28827 | -1.53 | 1 | | 1.01 | Medium |
| AEAIE | 5 | | 532.26132 | 532.26007 | -2.34 | 1 | 312612.4063 | 1.01 | Medium |
| EAAIE | 5 | | 532.26132 | 532.26007 | -2.34 | 1 | 312612.4063 | 1.01 | Medium |
| ESWNDSEYNYE | 11 | | 1435.52844 | 479.17944 | -3.25 | 3 | 287152.5938 | 1.01 | Medium |
| GTGPM | 5 | 1xOxidation [M5] | 478.19661 | 478.19525 | -2.84 | 1 | 3063435.125 | 1.01 | Medium |
| VGADAG | 6 | | 489.23035 | 489.22906 | -2.63 | 1 | 508349.3438 | 1.01 | Medium |
| ANPDQA | 6 | | 615.27328 | 615.27185 | -2.32 | 1 | 764746.875 | 1.01 | Medium |
| ANPDAQ | 6 | | 615.27328 | 615.27185 | -2.32 | 1 | 764746.875 | 1.01 | Medium |
| ESGLQ | 5 | | 533.25657 | 533.25555 | -1.9 | 1 | 76086.60156 | 1.01 | Medium |
| VAPE | 4 | | 415.21873 | 415.21756 | -2.81 | 1 | 1389837.25 | 1.01 | Medium |
| EGIGG | 5 | | 432.20889 | 432.20767 | -2.82 | 1 | 2907583.594 | 1 | Medium |
| EGIN | 4 | | 432.20889 | 432.20767 | -2.82 | 1 | 3372071.719 | 1 | Medium |
| GAETV | 5 | | 476.2351 | 476.23416 | -1.98 | 1 | 824966.0625 | 1 | Medium |
| QETV | 4 | | 476.2351 | 476.23416 | -1.98 | 1 | 824966.0625 | 1 | Medium |
| AGETV | 5 | | 476.2351 | 476.23416 | -1.98 | 1 | 824966.0625 | 1 | Medium |
| PSSPTA | 6 | | 559.27222 | 559.27081 | -2.51 | 1 | 645040.625 | 0.99 | Medium |
| ELTAG | 5 | | 490.25075 | 490.25 | -1.54 | 1 | 139118.8438 | 0.99 | Medium |
| EITGA | 5 | | 490.25075 | 490.25 | -1.54 | 1 | 139118.8438 | 0.99 | Medium |
| EITQ | 4 | | 490.25075 | 490.25 | -1.54 | 1 | 139118.8438 | 0.99 | Medium |
| PQAAE | 5 | | 515.246 | 515.24493 | -2.07 | 1 | | 0.99 | Medium |
| EGDASLSSVLDS | 12 | | 1179.53755 | 590.27753 | 8.67 | 2 | | 0.99 | Medium |
| EAVAG | 5 | | 446.22454 | 446.22342 | -2.51 | 1 | 2678526.75 | 0.99 | Medium |
| EAVQ | 4 | | 446.22454 | 446.22342 | -2.51 | 1 | 2678526.75 | 0.99 | Medium |
| EAVGA | 5 | | 446.22454 | 446.22342 | -2.51 | 1 | 2678526.75 | 0.99 | Medium |
| AEGINE | 6 | | 632.2886 | 632.28723 | -2.16 | 1 | 155631.5469 | 0.98 | Medium |
| PSQPE | 5 | | 557.25657 | 557.25488 | -3.02 | 1 | 157339.8594 | 0.98 | Medium |
| SPQPE | 5 | | 557.25657 | 557.25488 | -3.02 | 1 | 157339.8594 | 0.98 | Medium |
| DSGTTV | 6 | | 579.26205 | 579.26111 | -1.62 | 1 | | 0.98 | Medium |
| DSGIQ | 5 | | 519.24092 | 519.24005 | -1.67 | 1 | 119091.7891 | 0.98 | Medium |
| DSGLGA | 6 | | 519.24092 | 519.24005 | -1.67 | 1 | 119091.7891 | 0.98 | Medium |
| SDGIQ | 5 | | 519.24092 | 519.24005 | -1.67 | 1 | 119091.7891 | 0.98 | Medium |
| ENIE | 4 | | 504.23002 | 504.22867 | -2.68 | 1 | 171416.0195 | 0.98 | Medium |
| KGEM | 4 | 1xOxidation [M4] | 480.21226 | 480.21094 | -2.76 | 1 | 912137.5938 | 0.98 | Medium |
| YGGEE | 5 | | 554.20928 | 554.20789 | -2.52 | 1 | 1152987.875 | 0.97 | Medium |
| YNEE | 4 | | 554.20928 | 554.20789 | -2.52 | 1 | 1152987.875 | 0.97 | Medium |
| EGME | 4 | | 465.16498 | 465.16388 | -2.36 | 1 | 2933245.656 | 0.97 | Medium |

| | | | | | | | | | | |
|---|---|---|---|---|---|---|---|---|---|---|
| EGDIQ | 5 | | 561.25148 | 561.24982 | -2.97 | 1 | 364911.2813 | 0.97 | Medium |
| EITE | 4 | | 491.23477 | 491.2337 | -2.17 | 1 | 652662.2969 | 0.97 | Medium |
| EAGIQ | 5 | | 517.26165 | 517.26044 | -2.35 | 1 | 153158.9219 | 0.97 | Medium |
| EQLAG | 5 | | 517.26165 | 517.26044 | -2.35 | 1 | 153158.9219 | 0.97 | Medium |
| EQLGA | 5 | | 517.26165 | 517.26044 | -2.35 | 1 | 153158.9219 | 0.97 | Medium |
| EQIQ | 4 | | 517.26165 | 517.26044 | -2.35 | 1 | 153158.9219 | 0.97 | Medium |
| QEIN | 4 | | 503.246 | 503.2449 | -2.18 | 1 | 206554.0781 | 0.97 | Medium |
| GAEIN | 5 | | 503.246 | 503.2449 | -2.18 | 1 | 206554.0781 | 0.97 | Medium |
| QEIGG | 5 | | 503.246 | 503.2449 | -2.18 | 1 | 206554.0781 | 0.97 | Medium |
| DAGVAG | 6 | | 489.23035 | 489.22906 | -2.63 | 1 | 508349.3438 | 0.96 | Medium |
| EGAIQ | 5 | | 517.26165 | 517.26044 | -2.35 | 1 | 153158.9219 | 0.96 | Medium |
| ESRVE | 5 | | 619.30458 | 310.15521 | -2.31 | 2 | 120431.4609 | 0.96 | Medium |
| TGGTEQA | 7 | | 663.29441 | 663.29285 | -2.36 | 1 | 406054.0313 | 0.96 | Medium |
| VEASN | 5 | | 519.24092 | 519.23944 | -2.84 | 1 | 874010.125 | 0.96 | Medium |
| GVADQ | 5 | | 489.23035 | 489.22906 | -2.63 | 1 | 508349.3438 | 0.96 | Medium |
| DGEIE | 5 | | 562.2355 | 562.23334 | -3.84 | 1 | 63378.11328 | 0.96 | Medium |
| GDEIE | 5 | | 562.2355 | 562.23334 | -3.84 | 1 | 63378.11328 | 0.96 | Medium |
| EDIE | 4 | | 505.21403 | 505.21249 | -3.05 | 1 | 255417.6641 | 0.96 | Medium |
| ETIEE | 5 | | 620.27736 | 620.27539 | -3.18 | 1 | 548161.0625 | 0.96 | Medium |
| EQMGA | 5 | | 535.21807 | 535.21625 | -3.41 | 1 | 137246.8125 | 0.96 | Medium |
| EQMQ | 4 | | 535.21807 | 535.21625 | -3.41 | 1 | 137246.8125 | 0.96 | Medium |
| EGAMQ | 5 | | 535.21807 | 535.21625 | -3.41 | 1 | 137246.8125 | 0.96 | Medium |
| EAGMQ | 5 | | 535.21807 | 535.21625 | -3.41 | 1 | 137246.8125 | 0.96 | Medium |
| EIAQ | 4 | | 460.24019 | 460.23929 | -1.96 | 1 | 621564 | 0.95 | Medium |
| EIAAG | 5 | | 460.24019 | 460.23929 | -1.96 | 1 | 621564 | 0.95 | Medium |
| DGSLAG | 6 | | 519.24092 | 519.23999 | -1.79 | 1 | 150459.9531 | 0.95 | Medium |
| DGSIQ | 5 | | 519.24092 | 519.23999 | -1.79 | 1 | 150459.9531 | 0.95 | Medium |
| GDSLQ | 5 | | 519.24092 | 519.23999 | -1.79 | 1 | 150459.9531 | 0.95 | Medium |
| KVDGETGE | 8 | | 834.38395 | 417.69464 | -2.33 | 2 | 136075.4688 | 0.95 | Medium |
| EIAGA | 5 | | 460.24019 | 460.23929 | -1.96 | 1 | 621564 | 0.95 | Medium |
| SSGPTQ | 6 | | 576.26238 | 576.2608 | -2.74 | 1 | 2579463 | 0.95 | Medium |
| EVTN | 4 | | 462.21945 | 462.21854 | -1.99 | 1 | 165315.3594 | 0.94 | Medium |
| EVTGG | 5 | | 462.21945 | 462.21854 | -1.99 | 1 | 165315.3594 | 0.94 | Medium |
| GEGGI | 5 | | 432.20889 | 432.20776 | -2.6 | 1 | 464488.125 | 0.94 | Medium |
| ADGGI | 5 | | 432.20889 | 432.20776 | -2.6 | 1 | 464488.125 | 0.94 | Medium |
| DAGGI | 5 | | 432.20889 | 432.20776 | -2.6 | 1 | 464488.125 | 0.94 | Medium |
| EIGSGG | 6 | | 519.24092 | 519.23956 | -2.61 | 1 | 874010.125 | 0.94 | Medium |
| EIGSN | 5 | | 519.24092 | 519.23956 | -2.61 | 1 | 874010.125 | 0.94 | Medium |
| SEGI | 4 | | 405.19799 | 405.19702 | -2.39 | 1 | 157961.6406 | 0.94 | Medium |
| TDGI | 4 | | 405.19799 | 405.19702 | -2.39 | 1 | 190431.123 | 0.94 | Medium |
| VVDTE | 5 | | 562.27188 | 562.27136 | -0.93 | 1 | 65076.6875 | 0.94 | Medium |
| DTGI | 4 | | 405.19799 | 405.19702 | -2.39 | 1 | 190431.123 | 0.94 | Medium |
| ESGI | 4 | | 405.19799 | 405.19702 | -2.39 | 1 | 157961.6406 | 0.94 | Medium |
| VDSDPTE | 7 | | 762.3152 | 762.31305 | -2.83 | 1 | 123603.8516 | 0.94 | Medium |
| QAVEE | 5 | | 575.26713 | 575.26581 | -2.3 | 1 | 364776.2813 | 0.93 | Medium |
| AGAVEE | 6 | | 575.26713 | 575.26581 | -2.3 | 1 | 364776.2813 | 0.93 | Medium |
| AQVEE | 5 | | 575.26713 | 575.26581 | -2.3 | 1 | 364776.2813 | 0.93 | Medium |
| EGEM | 4 | 1xOxidation [M4] | 481.15989 | 481.15906 | -1.73 | 1 | 1126079.926 | 0.93 | Medium |
| GEEM | 4 | 1xOxidation [M4] | 481.15989 | 481.15906 | -1.73 | 1 | 1126079.926 | 0.93 | Medium |
| DAEM | 4 | 1xOxidation [M4] | 481.15989 | 481.15906 | -1.73 | 1 | 1126079.926 | 0.93 | Medium |
| ADEM | 4 | 1xOxidation [M4] | 481.15989 | 481.15906 | -1.73 | 1 | 1126079.926 | 0.93 | Medium |
| GGSIE | 5 | | 462.21945 | 462.21838 | -2.32 | 1 | 158415.2031 | 0.93 | Medium |
| NSIE | 4 | | 462.21945 | 462.21838 | -2.32 | 1 | 158415.2031 | 0.93 | Medium |
| SNIE | 4 | | 462.21945 | 462.21838 | -2.32 | 1 | 158415.2031 | 0.93 | Medium |
| NTMADI | 6 | 1xOxidation [M3] | 680.29197 | 680.29041 | -2.3 | 1 | 648450.8438 | 0.93 | Medium |
| SGGIE | 5 | | 462.21945 | 462.21838 | -2.32 | 1 | 158415.2031 | 0.93 | Medium |
| GSGIE | 5 | | 462.21945 | 462.21838 | -2.32 | 1 | 158415.2031 | 0.93 | Medium |
| WRFEPE | 6 | | 863.40463 | 432.20868 | 6.32 | 2 | | 0.92 | Medium |
| ADDID | 5 | | 548.21985 | 548.21857 | -2.34 | 1 | 509011.9375 | 0.92 | Medium |
| GEDID | 5 | | 548.21985 | 548.21857 | -2.34 | 1 | 509011.9375 | 0.92 | Medium |
| DADID | 5 | | 548.21985 | 548.21857 | -2.34 | 1 | 509011.9375 | 0.92 | Medium |
| EGDID | 5 | | 548.21985 | 548.21857 | -2.34 | 1 | 509011.9375 | 0.92 | Medium |
| GEIAN | 5 | | 503.246 | 503.24512 | -1.76 | 1 | 96256.36719 | 0.92 | Medium |
| DAIAGG | 6 | | 503.246 | 503.24512 | -1.76 | 1 | 96256.36719 | 0.92 | Medium |
| ADIAGG | 6 | | 503.246 | 503.24512 | -1.76 | 1 | 96256.36719 | 0.92 | Medium |
| DAIAN | 5 | | 503.246 | 503.24512 | -1.76 | 1 | 96256.36719 | 0.92 | Medium |
| GELAGG | 6 | | 503.246 | 503.24512 | -1.76 | 1 | 96256.36719 | 0.92 | Medium |
| ADIAN | 5 | | 503.246 | 503.24512 | -1.76 | 1 | 96256.36719 | 0.92 | Medium |
| DAIE | 4 | | 447.20855 | 447.20767 | -1.97 | 1 | 1024736.398 | 0.92 | Medium |
| ADIE | 4 | | 447.20855 | 447.20767 | -1.97 | 1 | 1046277.246 | 0.92 | Medium |
| GEIE | 4 | | 447.20855 | 447.20767 | -1.97 | 1 | 1046277.246 | 0.92 | Medium |
| QTAEQ | 5 | | 576.26238 | 576.26068 | -2.95 | 1 | 2579463 | 0.92 | Medium |
| QEMQ | 4 | | 535.21807 | 535.21625 | -3.41 | 1 | 137246.8125 | 0.92 | Medium |
| AGEMQ | 5 | | 535.21807 | 535.21625 | -3.41 | 1 | 137246.8125 | 0.92 | Medium |
| GAEMQ | 5 | | 535.21807 | 535.21625 | -3.41 | 1 | 137246.8125 | 0.92 | Medium |
| QTEAQ | 5 | | 576.26238 | 576.26068 | -2.95 | 1 | 2579463 | 0.92 | Medium |
| EGLAN | 5 | | 503.246 | 503.24512 | -1.76 | 1 | 96256.36719 | 0.92 | Medium |
| QDLAG | 5 | | 503.246 | 503.24542 | -1.15 | 1 | 91927.11719 | 0.92 | Medium |
| QDIQ | 4 | | 503.246 | 503.24542 | -1.15 | 1 | 91927.11719 | 0.92 | Medium |
| QDIGA | 5 | | 503.246 | 503.24542 | -1.15 | 1 | 91927.11719 | 0.92 | Medium |
| AGDLGA | 6 | | 503.246 | 503.24542 | -1.15 | 1 | 91927.11719 | 0.92 | Medium |
| GADLQ | 5 | | 503.246 | 503.24542 | -1.15 | 1 | 91927.11719 | 0.92 | Medium |
| AGDIAG | 6 | | 503.246 | 503.24542 | -1.15 | 1 | 91927.11719 | 0.92 | Medium |

| | | | | | | | | | |
|---|---|---|---|---|---|---|---|---|---|
| GADIAG | 6 | | 503.246 | 503.24542 | -1.15 | 1 | 91927.11719 | 0.92 | Medium |
| EGIE | 4 | | 447.20855 | 447.20767 | -1.97 | 1 | 1024736.398 | 0.92 | Medium |
| GEDGLT | 6 | | 591.26205 | 591.26056 | -2.52 | 1 | 12875939 | 0.92 | Medium |
| DADGIT | 6 | | 591.26205 | 591.26056 | -2.52 | 1 | 12875939 | 0.92 | Medium |
| EADI | 4 | | 447.20855 | 447.20758 | -2.18 | 1 | 1833809.344 | 0.92 | Medium |
| APAEAT | 6 | | 559.27222 | 559.27075 | -2.62 | 1 | 645040.625 | 0.92 | Medium |
| AGEIEE | 6 | | 647.28826 | 647.2868 | -2.25 | 1 | 437798.3438 | 0.91 | Medium |
| QEIEE | 5 | | 647.28826 | 647.2868 | -2.25 | 1 | 437798.3438 | 0.91 | Medium |
| SADDVDSH | 8 | | 845.32717 | 423.16702 | -0.47 | 2 | 287535.375 | 0.91 | Medium |
| QEEIE | 5 | | 647.28826 | 647.2868 | -2.25 | 1 | 437798.3438 | 0.91 | Medium |
| AGEEIE | 6 | | 647.28826 | 647.2868 | -2.25 | 1 | 437798.3438 | 0.91 | Medium |
| GISGGVGMILEAPP | 14 | | 1297.68205 | 649.33856 | -9.4 | 2 | | 0.91 | Medium |
| SPEDI | 5 | | 560.25623 | 560.2547 | -2.74 | 1 | 2384750.5 | 0.91 | Medium |
| DANMRVE | 7 | | 834.37743 | 417.69464 | 5.49 | 2 | 136075.4688 | 0.91 | Medium |
| KGECDV | 6 | | 650.2814 | 325.64331 | -3.16 | 2 | 7206699.875 | 0.91 | Medium |
| QEVAG | 5 | | 503.246 | 503.24496 | -2.06 | 1 | 206554.0781 | 0.91 | Medium |
| QEVQ | 4 | | 503.246 | 503.24496 | -2.06 | 1 | 206554.0781 | 0.91 | Medium |
| AGEVQ | 5 | | 503.246 | 503.24496 | -2.06 | 1 | 206554.0781 | 0.91 | Medium |
| GAEVQ | 5 | | 503.246 | 503.24496 | -2.06 | 1 | 206554.0781 | 0.91 | Medium |
| GSSID | 5 | | 478.21437 | 478.21313 | -2.58 | 1 | 1431645.125 | 0.9 | Medium |
| SGSID | 5 | | 478.21437 | 478.21313 | -2.58 | 1 | 1431645.125 | 0.9 | Medium |
| EATPQ | 5 | | 545.25657 | 545.25519 | -2.53 | 1 | 181865.0313 | 0.9 | Medium |
| AEIE | 4 | | 461.2242 | 461.22339 | -1.77 | 1 | 2039038.133 | 0.9 | Medium |
| ENIQ | 4 | | 503.246 | 503.24512 | -1.76 | 1 | 228505.1016 | 0.9 | Medium |
| ENIGA | 5 | | 503.246 | 503.24512 | -1.76 | 1 | 228505.1016 | 0.9 | Medium |
| ENLAG | 5 | | 503.246 | 503.24512 | -1.76 | 1 | 228505.1016 | 0.9 | Medium |
| EVAQ | 4 | | 446.22454 | 446.22305 | -3.33 | 1 | | 0.9 | Medium |
| EVAAG | 5 | | 446.22454 | 446.22305 | -3.33 | 1 | | 0.9 | Medium |
| EVAGA | 5 | | 446.22454 | 446.22305 | -3.33 | 1 | | 0.9 | Medium |
| GTEVA | 5 | | 476.2351 | 476.23428 | -1.72 | 1 | 659074.75 | 0.9 | Medium |
| ASEVA | 5 | | 476.2351 | 476.23428 | -1.72 | 1 | 659074.75 | 0.9 | Medium |
| TGEVA | 5 | | 476.2351 | 476.23428 | -1.72 | 1 | 1098544.156 | 0.9 | Medium |
| DIVDN | 5 | | 575.26713 | 575.26447 | -4.64 | 1 | 84166.52344 | 0.9 | Medium |
| EVVDN | 5 | | 575.26713 | 575.26447 | -4.64 | 1 | 84166.52344 | 0.9 | Medium |
| PSDAP | 5 | | 486.21945 | 486.2186 | -1.76 | 1 | 461636.1875 | 0.9 | Medium |
| EGGIQ | 5 | | 503.246 | 503.24512 | -1.76 | 1 | 290581.0469 | 0.9 | Medium |
| GETVA | 5 | | 476.2351 | 476.23428 | -1.72 | 1 | 967815.0313 | 0.9 | Medium |
| ADTVA | 5 | | 476.2351 | 476.23428 | -1.72 | 1 | 659074.75 | 0.9 | Medium |
| DATVA | 5 | | 476.2351 | 476.23428 | -1.72 | 1 | 659074.75 | 0.89 | Medium |
| EGTVA | 5 | | 476.2351 | 476.23428 | -1.72 | 1 | 659074.75 | 0.89 | Medium |
| EGVGA | 5 | | 432.20889 | 432.20801 | -2.04 | 1 | 1102079.906 | 0.89 | Medium |
| EGVAG | 5 | | 432.20889 | 432.20801 | -2.04 | 1 | 637591.7813 | 0.89 | Medium |
| EGVQ | 4 | | 432.20889 | 432.20801 | -2.04 | 1 | 637591.7813 | 0.89 | Medium |
| PTDSAQ | 6 | | 618.27295 | 618.27124 | -2.76 | 1 | 4150965.25 | 0.89 | Medium |
| TSELQ | 5 | | 577.28278 | 577.28131 | -2.55 | 1 | 103142.8047 | 0.89 | Medium |
| STEIQ | 5 | | 577.28278 | 577.28131 | -2.55 | 1 | 103142.8047 | 0.89 | Medium |
| ADDDL | 5 | | 548.21985 | 548.2182 | -3 | 1 | 509011.9375 | 0.88 | Medium |
| DADDL | 5 | | 548.21985 | 548.2182 | -3 | 1 | 509011.9375 | 0.88 | Medium |
| GEDEV | 5 | | 548.21985 | 548.2182 | -3 | 1 | 509011.9375 | 0.88 | Medium |
| GEDVE | 5 | | 548.21985 | 548.2182 | -3 | 1 | 509011.9375 | 0.88 | Medium |
| ADDEV | 5 | | 548.21985 | 548.2182 | -3 | 1 | 509011.9375 | 0.88 | Medium |
| EGDDL | 5 | | 548.21985 | 548.2182 | -3 | 1 | 509011.9375 | 0.88 | Medium |
| DADVE | 5 | | 548.21985 | 548.2182 | -3 | 1 | 509011.9375 | 0.88 | Medium |
| GYDAD | 5 | | 540.19363 | 540.19238 | -2.31 | 1 | 745680.8125 | 0.88 | Medium |
| QELRDYQ | 7 | | 951.45304 | 476.23337 | 6.75 | 2 | 308740.2813 | 0.88 | Medium |
| QEFRPFQ | 7 | | 951.46829 | 476.23337 | -9.28 | 2 | 308740.2813 | 0.88 | Medium |
| VGDPTA | 6 | | 559.27222 | 559.27075 | -2.62 | 1 | 645040.625 | 0.88 | Medium |
| QEYLDRQ | 7 | | 951.45304 | 476.23337 | 6.75 | 2 | 308740.2813 | 0.88 | Medium |
| QPDRQ | 5 | | 643.31581 | 643.3125 | -5.15 | 1 | 116168.1875 | 0.88 | Medium |
| QETWLFQ | 7 | | 951.45706 | 476.23337 | 2.52 | 2 | 308740.2813 | 0.88 | Medium |
| IDDGSD | 6 | | 621.23623 | 621.23456 | -2.68 | 1 | | 0.88 | Medium |
| QRPDQ | 5 | | 643.31581 | 643.3125 | -5.15 | 1 | 116168.1875 | 0.87 | Medium |
| QRDPQ | 5 | | 643.31581 | 643.3125 | -5.15 | 1 | 116168.1875 | 0.87 | Medium |
| IDGN | 4 | | 418.19324 | 418.19214 | -2.63 | 1 | 211149.3594 | 0.87 | Medium |
| EVGN | 4 | | 418.19324 | 418.19214 | -2.63 | 1 | 211149.3594 | 0.87 | Medium |
| DIGN | 4 | | 418.19324 | 418.19214 | -2.63 | 1 | 211149.3594 | 0.87 | Medium |
| VEGN | 4 | | 418.19324 | 418.19214 | -2.63 | 1 | 211149.3594 | 0.87 | Medium |
| EVGGG | 5 | | 418.19324 | 418.19214 | -2.63 | 1 | 211149.3594 | 0.87 | Medium |
| DIGGG | 5 | | 418.19324 | 418.19214 | -2.63 | 1 | 211149.3594 | 0.87 | Medium |
| LDGGG | 5 | | 418.19324 | 418.19214 | -2.63 | 1 | 211149.3594 | 0.87 | Medium |
| DNGSIS | 6 | | 592.2573 | 592.25598 | -2.22 | 1 | 390012.4688 | 0.87 | Medium |
| AQGPT | 5 | | 473.23544 | 473.23401 | -3.02 | 1 | 487367.0254 | 0.87 | Medium |
| AQGTP | 5 | | 473.23544 | 473.23401 | -3.02 | 1 | 456919.625 | 0.87 | Medium |
| AASQGTMN | 8 | 1xOxidation [M7] | 795.33014 | 795.32446 | -7.14 | 1 | 22354596 | 0.87 | Medium |
| AGAGPT | 6 | | 473.23544 | 473.23401 | -3.02 | 1 | 487367.0254 | 0.87 | Medium |
| VESPE | 5 | | 560.25623 | 560.2547 | -2.74 | 1 | 2384750.5 | 0.87 | Medium |
| DISPE | 5 | | 560.25623 | 560.2547 | -2.74 | 1 | 2768187.906 | 0.87 | Medium |
| IDSPE | 5 | | 560.25623 | 560.2547 | -2.74 | 1 | 2384750.5 | 0.87 | Medium |
| EVSPE | 5 | | 560.25623 | 560.2547 | -2.74 | 1 | 2797302.793 | 0.87 | Medium |
| PGSSGE | 6 | | 533.22018 | 533.21918 | -1.88 | 1 | 201939.4063 | 0.87 | Medium |
| TEMT | 4 | | 481.19628 | 481.19507 | -2.51 | 1 | 13000822.38 | 0.87 | Medium |
| ETMT | 4 | | 481.19628 | 481.19507 | -2.51 | 1 | 13000822.38 | 0.87 | Medium |
| VETAE | 5 | | 548.25623 | 548.25488 | -2.46 | 1 | 1335599 | 0.87 | Medium |

| | | | | | | | | | | |
|---|---|---|---|---|---|---|---|---|---|---|
| ESTLQ | 5 | | 577.28278 | 577.28131 | -2.55 | 1 | 103142.8047 | 0.87 | Medium |
| DITAE | 5 | | 548.25623 | 548.25488 | -2.46 | 1 | 1335599 | 0.87 | Medium |
| IDTAE | 5 | | 548.25623 | 548.25488 | -2.46 | 1 | 1335599 | 0.87 | Medium |
| TGVE | 4 | | 405.19799 | 405.1972 | -1.94 | 1 | 1460816.203 | 0.87 | Medium |
| SAVE | 4 | | 405.19799 | 405.1972 | -1.94 | 1 | 1460816.203 | 0.87 | Medium |
| ASVE | 4 | | 405.19799 | 405.1972 | -1.94 | 1 | 1460816.203 | 0.87 | Medium |
| GTVE | 4 | | 405.19799 | 405.1972 | -1.94 | 1 | 1460816.203 | 0.87 | Medium |
| AAPPS | 5 | | 442.22962 | 442.22836 | -2.85 | 1 | 476271.8281 | 0.87 | Medium |
| EVTAE | 5 | | 548.25623 | 548.25488 | -2.46 | 1 | 1335599 | 0.87 | Medium |
| EVFYE | 5 | | 686.30318 | 686.30884 | 8.24 | 1 | 495271.9688 | 0.86 | Medium |
| SVADPTTIN | 9 | | 917.45745 | 459.2283 | -8.86 | 2 | | 0.86 | Medium |
| GEAM | 4 | | 407.1595 | 407.1582 | -3.18 | 1 | 87302.07813 | 0.86 | Medium |
| ADAM | 4 | | 407.1595 | 407.1582 | -3.18 | 1 | 87302.07813 | 0.86 | Medium |
| DAAM | 4 | | 407.1595 | 407.1582 | -3.18 | 1 | 87302.07813 | 0.86 | Medium |
| EGAM | 4 | | 407.1595 | 407.1582 | -3.18 | 1 | 326119.4219 | 0.86 | Medium |
| TNDVAGD | 7 | | 691.28932 | 691.2879 | -2.06 | 1 | | 0.86 | Medium |
| QSDNID | 6 | | 691.28932 | 691.2879 | -2.06 | 1 | | 0.86 | Medium |
| QSDDQV | 6 | | 691.28932 | 691.2879 | -2.06 | 1 | | 0.86 | Medium |
| ASGDNLD | 7 | | 691.28932 | 691.2879 | -2.06 | 1 | | 0.86 | Medium |
| SIEAE | 5 | | 548.25623 | 548.25488 | -2.46 | 1 | 1208972.75 | 0.86 | Medium |
| VTEAE | 5 | | 548.25623 | 548.25488 | -2.46 | 1 | 1208972.75 | 0.86 | Medium |
| LSEAE | 5 | | 548.25623 | 548.25488 | -2.46 | 1 | 1208972.75 | 0.86 | Medium |
| QSDQVD | 6 | | 691.28932 | 691.2879 | -2.06 | 1 | | 0.86 | Medium |
| SSIE | 4 | | 435.20855 | 435.20758 | -2.24 | 1 | 404618.0938 | 0.86 | Medium |
| TDGTLS | 6 | | 593.2777 | 593.27631 | -2.34 | 1 | 233365.9063 | 0.86 | Medium |
| DTGTVT | 6 | | 593.2777 | 593.27631 | -2.34 | 1 | 233365.9063 | 0.86 | Medium |
| PDSPA | 5 | | 486.21945 | 486.2186 | -1.76 | 1 | 461636.1875 | 0.86 | Medium |
| DPSPA | 5 | | 486.21945 | 486.2186 | -1.76 | 1 | 461636.1875 | 0.86 | Medium |
| DPSAP | 5 | | 486.21945 | 486.2186 | -1.76 | 1 | 461636.1875 | 0.86 | Medium |
| AGEIE | 5 | | 518.24567 | 518.24445 | -2.36 | 1 | 194514.5117 | 0.86 | Medium |
| QEIE | 4 | | 518.24567 | 518.24445 | -2.36 | 1 | 300896.4316 | 0.86 | Medium |
| GAEIE | 5 | | 518.24567 | 518.24445 | -2.36 | 1 | 194514.5117 | 0.86 | Medium |
| VETGA | 5 | | 476.2351 | 476.2341 | -2.11 | 1 | 1079719.781 | 0.86 | Medium |
| IDTAG | 5 | | 476.2351 | 476.2341 | -2.11 | 1 | 770979.5 | 0.86 | Medium |
| IDTQ | 4 | | 476.2351 | 476.2341 | -2.11 | 1 | 770979.5 | 0.86 | Medium |
| IDTGA | 5 | | 476.2351 | 476.2341 | -2.11 | 1 | 1079719.781 | 0.86 | Medium |
| DLTAG | 5 | | 476.2351 | 476.2341 | -2.11 | 1 | 770979.5 | 0.86 | Medium |
| VETQ | 4 | | 476.2351 | 476.2341 | -2.11 | 1 | 770979.5 | 0.86 | Medium |
| DLTQ | 4 | | 476.2351 | 476.2341 | -2.11 | 1 | 770979.5 | 0.86 | Medium |
| GEGAI | 5 | | 446.22454 | 446.22363 | -2.03 | 1 | 276288.3438 | 0.85 | Medium |
| ADGAI | 5 | | 446.22454 | 446.22363 | -2.03 | 1 | 276288.3438 | 0.85 | Medium |
| DAGAI | 5 | | 446.22454 | 446.22363 | -2.03 | 1 | 276288.3438 | 0.85 | Medium |
| DAAIE | 5 | | 518.24567 | 518.24445 | -2.36 | 1 | 405648.1992 | 0.85 | Medium |
| ADAIE | 5 | | 518.24567 | 518.24445 | -2.36 | 1 | 123907.9805 | 0.85 | Medium |
| GEALE | 5 | | 518.24567 | 518.24445 | -2.36 | 1 | 48236.15234 | 0.85 | Medium |
| DALN | 4 | | 432.20889 | 432.20773 | -2.67 | 1 | 2849802.172 | 0.85 | Medium |
| GELGG | 5 | | 432.20889 | 432.20773 | -2.67 | 1 | 667305.7969 | 0.85 | Medium |
| ADIN | 4 | | 432.20889 | 432.20773 | -2.67 | 1 | 2385314.047 | 0.85 | Medium |
| GEIN | 4 | | 432.20889 | 432.20773 | -2.67 | 1 | 667305.7969 | 0.85 | Medium |
| DAIGG | 5 | | 432.20889 | 432.20773 | -2.67 | 1 | 2385314.047 | 0.85 | Medium |
| ASPGSTGG | 8 | | 633.28385 | 633.28204 | -2.84 | 1 | 365177.625 | 0.85 | Medium |
| ASMQ | 4 | | 436.18605 | 436.18503 | -2.33 | 1 | 55433.38672 | 0.85 | Medium |
| ASMGA | 5 | | 436.18605 | 436.18503 | -2.33 | 1 | 55433.38672 | 0.85 | Medium |
| ASMAG | 5 | | 436.18605 | 436.18503 | -2.33 | 1 | 55433.38672 | 0.85 | Medium |
| GTMQ | 4 | | 436.18605 | 436.18503 | -2.33 | 1 | 55433.38672 | 0.85 | Medium |
| SAMQ | 4 | | 436.18605 | 436.18503 | -2.33 | 1 | 55433.38672 | 0.85 | Medium |
| TGMAG | 5 | | 436.18605 | 436.18503 | -2.33 | 1 | 55433.38672 | 0.85 | Medium |
| TGMQ | 4 | | 436.18605 | 436.18503 | -2.33 | 1 | 55433.38672 | 0.85 | Medium |
| SAMGA | 5 | | 436.18605 | 436.18503 | -2.33 | 1 | 55433.38672 | 0.85 | Medium |
| EGGAI | 5 | | 446.22454 | 446.22363 | -2.03 | 1 | 276288.3438 | 0.85 | Medium |
| FDNS | 4 | | 482.18815 | 482.18698 | -2.43 | 1 | 176090.4219 | 0.85 | Medium |
| GEDISQ | 6 | | 648.28351 | 648.28186 | -2.55 | 1 | | 0.85 | Medium |
| DADLSQ | 6 | | 648.28351 | 648.28186 | -2.55 | 1 | | 0.85 | Medium |
| FDGGS | 5 | | 482.18815 | 482.18698 | -2.43 | 1 | 176090.4219 | 0.85 | Medium |
| ENAGNP | 6 | | 601.25763 | 601.25653 | -1.83 | 1 | 935708.25 | 0.85 | Medium |
| EEAYAYNA | 8 | | 930.38395 | 465.69244 | -6.82 | 2 | 405452.5938 | 0.85 | Medium |
| SADASI | 6 | | 563.26713 | 563.26581 | -2.35 | 1 | | 0.85 | Medium |
| TGDASI | 6 | | 563.26713 | 563.26581 | -2.35 | 1 | | 0.85 | Medium |
| SIEEYM | 6 | 1xOxidation [M6] | 787.31785 | 394.16336 | 2.03 | 2 | | 0.84 | Medium |
| TVEEYM | 6 | 1xOxidation [M6] | 787.31785 | 394.16336 | 2.03 | 2 | | 0.84 | Medium |
| DETI | 4 | | 477.21912 | 477.21799 | -2.37 | 1 | 240038.4531 | 0.84 | Medium |
| ETQLFWQ | 7 | | 951.45706 | 476.23349 | 2.78 | 2 | 308740.2813 | 0.84 | Medium |
| EDTI | 4 | | 477.21912 | 477.21799 | -2.37 | 1 | 240038.4531 | 0.84 | Medium |
| QDIE | 4 | | 504.23002 | 504.2291 | -1.83 | 1 | 228343.5508 | 0.84 | Medium |
| AGDIE | 5 | | 504.23002 | 504.2291 | -1.83 | 1 | 100020.6563 | 0.84 | Medium |
| GADIE | 5 | | 504.23002 | 504.2291 | -1.83 | 1 | 228343.5508 | 0.84 | Medium |
| YEFVE | 5 | | 686.30318 | 686.30884 | 8.24 | 1 | 495271.9688 | 0.83 | Medium |
| EIPE | 4 | | 487.23986 | 487.23788 | -4.04 | 1 | 314688.043 | 0.83 | Medium |
| YFEVE | 5 | | 686.30318 | 686.30884 | 8.24 | 1 | 495271.9688 | 0.83 | Medium |
| KGEIIPGQEETP | 12 | | 1297.66342 | 649.3385 | 4.86 | 2 | | 0.83 | Medium |
| VVAD | 4 | | 403.21873 | 403.21753 | -2.97 | 1 | 633697.25 | 0.83 | Medium |
| IEAA | 4 | | 403.21873 | 403.21768 | -2.59 | 1 | 445298.9688 | 0.83 | Medium |
| GEIST | 5 | | 506.24567 | 506.24454 | -2.23 | 1 | 387881.2813 | 0.83 | Medium |

| | | | | | | | | | |
|---|---|---|---|---|---|---|---|---|---|
| DALST | 5 | | 506.24567 | 506.24454 | -2.23 | 1 | 387881.2813 | 0.83 | Medium |
| EGIST | 5 | | 506.24567 | 506.24454 | -2.23 | 1 | 387881.2813 | 0.83 | Medium |
| EIAA | 4 | | 403.21873 | 403.21768 | -2.59 | 1 | 445298.9688 | 0.83 | Medium |
| SEDLLSTDGA | 10 | | 1007.45276 | 504.22913 | -1.77 | 2 | 57444.13672 | 0.83 | Medium |
| TNIE | 4 | | 476.2351 | 476.23386 | -2.62 | 1 | 298253.0508 | 0.83 | Medium |
| SQIE | 4 | | 476.2351 | 476.23386 | -2.62 | 1 | 298253.0508 | 0.83 | Medium |
| NTIE | 4 | | 476.2351 | 476.23386 | -2.62 | 1 | 298253.0508 | 0.83 | Medium |
| ISDIQMPN | 8 | | 917.43969 | 459.22687 | 7.37 | 2 | | 0.83 | Medium |
| DSAIQ | 5 | | 533.25657 | 533.25537 | -2.24 | 1 | 76086.60156 | 0.83 | Medium |
| DSAIAG | 6 | | 533.25657 | 533.25537 | -2.24 | 1 | 76086.60156 | 0.83 | Medium |
| SDAIAG | 6 | | 533.25657 | 533.25537 | -2.24 | 1 | 76086.60156 | 0.83 | Medium |
| DATI | 4 | | 419.21364 | 419.2131 | -1.28 | 1 | 254591.25 | 0.83 | Medium |
| GETI | 4 | | 419.21364 | 419.2131 | -1.28 | 1 | 2902680.25 | 0.83 | Medium |
| ADTI | 4 | | 419.21364 | 419.2131 | -1.28 | 1 | 2902680.25 | 0.83 | Medium |
| SLGDMAIPN | 9 | | 917.43969 | 459.22687 | 7.37 | 2 | | 0.83 | Medium |
| VVDSAN | 6 | | 604.29368 | 604.29504 | 2.25 | 1 | 77637.95313 | 0.83 | Medium |
| EGTI | 4 | | 419.21364 | 419.2131 | -1.28 | 1 | 2706572.719 | 0.83 | Medium |
| GTDI | 4 | | 405.19799 | 405.19699 | -2.47 | 1 | 1615788.438 | 0.83 | Medium |
| SADI | 4 | | 405.19799 | 405.19699 | -2.47 | 1 | 1615788.438 | 0.83 | Medium |
| TGDI | 4 | | 405.19799 | 405.19699 | -2.47 | 1 | 1264484.906 | 0.83 | Medium |
| ASDI | 4 | | 405.19799 | 405.19699 | -2.47 | 1 | 1615788.438 | 0.83 | Medium |
| SPSSP | 5 | | 474.21945 | 474.21832 | -2.39 | 1 | 47073.75781 | 0.83 | Medium |
| PSSSP | 5 | | 474.21945 | 474.21832 | -2.39 | 1 | 47073.75781 | 0.83 | Medium |
| SPSPS | 5 | | 474.21945 | 474.21832 | -2.39 | 1 | 47073.75781 | 0.83 | Medium |
| PSSPS | 5 | | 474.21945 | 474.21832 | -2.39 | 1 | 47073.75781 | 0.83 | Medium |
| TEIE | 4 | | 491.23477 | 491.2337 | -2.17 | 1 | 440270.3594 | 0.82 | Medium |
| ETIE | 4 | | 491.23477 | 491.2337 | -2.17 | 1 | 440270.3594 | 0.82 | Medium |
| DESI | 4 | | 463.20347 | 463.20251 | -2.06 | 1 | 436600.5938 | 0.82 | Medium |
| GIPQ | 4 | | 414.23471 | 414.23355 | -2.8 | 1 | 113345.4063 | 0.82 | Medium |
| AVPQ | 4 | | 414.23471 | 414.23355 | -2.8 | 1 | 113345.4063 | 0.82 | Medium |
| GLPGA | 5 | | 414.23471 | 414.23355 | -2.8 | 1 | 113345.4063 | 0.82 | Medium |
| GIPAG | 5 | | 414.23471 | 414.23355 | -2.8 | 1 | 113345.4063 | 0.82 | Medium |
| AVPGA | 5 | | 414.23471 | 414.23355 | -2.8 | 1 | 113345.4063 | 0.82 | Medium |
| VAPGA | 5 | | 414.23471 | 414.23355 | -2.8 | 1 | 113345.4063 | 0.82 | Medium |
| AVPAG | 5 | | 414.23471 | 414.23355 | -2.8 | 1 | 113345.4063 | 0.82 | Medium |
| VAPAG | 5 | | 414.23471 | 414.23355 | -2.8 | 1 | 113345.4063 | 0.82 | Medium |
| VAPQ | 4 | | 414.23471 | 414.23355 | -2.8 | 1 | 113345.4063 | 0.82 | Medium |
| EDSI | 4 | | 463.20347 | 463.20251 | -2.06 | 1 | 436600.5938 | 0.82 | Medium |
| VVAGMVDPN | 9 | 1xOxidation [M5] | 917.43969 | 459.22626 | 6.04 | 2 | | 0.82 | Medium |
| STTPT | 5 | | 506.24567 | 506.24451 | -2.3 | 1 | 495440.25 | 0.82 | Medium |
| TSTPT | 5 | | 506.24567 | 506.24451 | -2.3 | 1 | 495440.25 | 0.82 | Medium |
| TAGGVSHAR | 9 | | 855.44314 | 428.22397 | -2.9 | 2 | | 0.82 | Medium |
| PGSGAT | 6 | | 489.23035 | 489.22922 | -2.32 | 1 | 725356.7344 | 0.82 | Medium |
| SQVTE | 5 | | 563.26713 | 563.26611 | -1.81 | 1 | 67616.6875 | 0.82 | Medium |
| SAGVTE | 6 | | 563.26713 | 563.26611 | -1.81 | 1 | 67616.6875 | 0.82 | Medium |
| TNVTE | 5 | | 563.26713 | 563.26611 | -1.81 | 1 | 67616.6875 | 0.82 | Medium |
| EDEIE | 5 | | 634.25663 | 634.25507 | -2.46 | 1 | 44017.64453 | 0.82 | Medium |
| DEEIE | 5 | | 634.25663 | 634.25507 | -2.46 | 1 | 44017.64453 | 0.82 | Medium |
| ESVGREFQ | 8 | | 951.45304 | 476.23349 | 7.01 | 2 | 1133706.344 | 0.82 | Medium |
| EEHGSAAET | 9 | | 930.37993 | 465.69244 | -2.49 | 2 | 405452.5938 | 0.82 | Medium |
| TSIE | 4 | | 449.2242 | 449.22342 | -1.75 | 1 | 181053.4531 | 0.82 | Medium |
| STIE | 4 | | 449.2242 | 449.22342 | -1.75 | 1 | 181053.4531 | 0.82 | Medium |
| EDTLQ | 5 | | 605.2777 | 605.27716 | -0.89 | 1 | 173630.7813 | 0.82 | Medium |
| AIGAE | 5 | | 460.24019 | 460.2395 | -1.49 | 1 | 118150.7422 | 0.82 | Medium |
| LAGAE | 5 | | 460.24019 | 460.2395 | -1.49 | 1 | 118150.7422 | 0.82 | Medium |
| ENLGG | 5 | | 489.23035 | 489.2291 | -2.57 | 1 | 4487008.5 | 0.82 | Medium |
| ENIN | 4 | | 489.23035 | 489.2291 | -2.57 | 1 | 4487008.5 | 0.82 | Medium |
| ENVAG | 5 | | 489.23035 | 489.2291 | -2.57 | 1 | 4487008.5 | 0.82 | Medium |
| ENVQ | 4 | | 489.23035 | 489.2291 | -2.57 | 1 | 4995357.844 | 0.82 | Medium |
| AEDIN | 5 | | 561.25148 | 561.25 | -2.64 | 1 | 814768.9375 | 0.82 | Medium |
| AEDLGG | 6 | | 561.25148 | 561.25 | -2.64 | 1 | 732084 | 0.82 | Medium |
| AEELN | 5 | | 575.26713 | 575.26599 | -1.98 | 1 | 91075.6875 | 0.81 | Medium |
| ADEI | 4 | | 447.20855 | 447.20755 | -2.25 | 1 | 321291.0195 | 0.81 | Medium |
| GEEL | 4 | | 447.20855 | 447.20755 | -2.25 | 1 | 321291.0195 | 0.81 | Medium |
| EADIN | 5 | | 561.25148 | 561.25 | -2.64 | 1 | 814768.9375 | 0.81 | Medium |
| TIGE | 4 | | 419.21364 | 419.21259 | -2.52 | 1 | 2988308.531 | 0.81 | Medium |
| ITGE | 4 | | 419.21364 | 419.21259 | -2.52 | 1 | 2988308.531 | 0.81 | Medium |
| VPQE | 4 | | 472.24019 | 472.23889 | -2.75 | 1 | 128770.5469 | 0.81 | Medium |
| EAEIN | 5 | | 575.26713 | 575.26599 | -1.98 | 1 | 91075.6875 | 0.81 | Medium |
| VPGAE | 5 | | 472.24019 | 472.23889 | -2.75 | 1 | 128770.5469 | 0.81 | Medium |
| NGIE | 4 | | 432.20889 | 432.20786 | -2.39 | 1 | 1900991.828 | 0.81 | Medium |
| GNIE | 4 | | 432.20889 | 432.20786 | -2.39 | 1 | 1900991.828 | 0.81 | Medium |
| GGGIE | 5 | | 432.20889 | 432.20786 | -2.39 | 1 | 1900991.828 | 0.81 | Medium |
| TDGGVA | 6 | | 519.24092 | 519.23999 | -1.79 | 1 | 129749.1094 | 0.81 | Medium |
| SEGGLG | 6 | | 519.24092 | 519.23999 | -1.79 | 1 | 129749.1094 | 0.81 | Medium |
| ESGGIG | 6 | | 519.24092 | 519.23999 | -1.79 | 1 | 129749.1094 | 0.81 | Medium |
| VPAEG | 5 | | 472.24019 | 472.23889 | -2.75 | 1 | 128770.5469 | 0.81 | Medium |
| VPADA | 5 | | 472.24019 | 472.23889 | -2.75 | 1 | 128770.5469 | 0.81 | Medium |
| VPAGE | 5 | | 472.24019 | 472.23889 | -2.75 | 1 | 128770.5469 | 0.81 | Medium |
| EGDVE | 5 | | 548.21985 | 548.2182 | -3 | 1 | 509011.9375 | 0.81 | Medium |
| DSSSTM | 6 | | 627.22903 | 627.22778 | -1.99 | 1 | 59176.82813 | 0.81 | Medium |
| SESSAPE | 7 | | 706.28899 | 706.2879 | -1.54 | 1 | 72499.42188 | 0.81 | Medium |
| KGAVMQ | 6 | 1xOxidation [M5] | 649.33377 | 649.33844 | 7.19 | 1 | | 0.81 | Medium |

| | | | | | | | | | |
|---|---|---|---|---|---|---|---|---|---|
| KGKEMG | 6 | | 649.33377 | 649.33844 | 7.19 | 1 | 122317.8906 | 0.81 | Medium |
| ESEIQ | 5 | | 605.2777 | 605.27716 | -0.89 | 1 | 60707.97656 | 0.8 | Medium |
| ESELAG | 6 | | 605.2777 | 605.27716 | -0.89 | 1 | 60707.97656 | 0.8 | Medium |
| EVTGA | 5 | | 476.2351 | 476.23407 | -2.17 | 1 | 1314238.172 | 0.8 | Medium |
| ELGLQMAGA | 9 | | 889.44478 | 445.22845 | 5.46 | 2 | 617603.375 | 0.8 | Medium |
| GDIE | 4 | | 433.1929 | 433.19177 | -2.61 | 1 | 89982.14063 | 0.8 | Medium |
| DGIE | 4 | | 433.1929 | 433.19177 | -2.61 | 1 | 89982.14063 | 0.8 | Medium |
| PGGSAT | 6 | | 489.23035 | 489.22922 | -2.32 | 1 | 217007.3906 | 0.8 | Medium |
| WEEGKLQ | 7 | | 889.44141 | 445.22833 | 8.97 | 2 | 617603.375 | 0.8 | Medium |
| AAAPE | 5 | | 458.22454 | 458.22354 | -2.18 | 1 | 317965.4824 | 0.8 | Medium |
| GVGPE | 5 | | 458.22454 | 458.22354 | -2.18 | 1 | 317965.4824 | 0.8 | Medium |
| VNPE | 4 | | 458.22454 | 458.22354 | -2.18 | 1 | 317965.4824 | 0.8 | Medium |
| NVPE | 4 | | 458.22454 | 458.22354 | -2.18 | 1 | 317965.4824 | 0.8 | Medium |
| GINE | 4 | | 432.20889 | 432.20801 | -2.04 | 1 | 1023800.063 | 0.8 | Medium |
| IGNE | 4 | | 432.20889 | 432.20801 | -2.04 | 1 | 1023800.063 | 0.8 | Medium |
| AVNE | 4 | | 432.20889 | 432.20801 | -2.04 | 1 | 1023800.063 | 0.8 | Medium |
| VANE | 4 | | 432.20889 | 432.20801 | -2.04 | 1 | 1023800.063 | 0.8 | Medium |
| WGRTELQ | 7 | | 889.45264 | 445.22833 | -3.66 | 2 | 617603.375 | 0.8 | Medium |
| DIISTDDL | 8 | | 891.43057 | 446.22308 | 9.34 | 2 | 126756.1719 | 0.8 | Medium |
| MGVKNSPE | 8 | | 861.41348 | 431.21259 | 5.13 | 2 | 251528.0313 | 0.8 | Medium |
| LGGGE | 5 | | 432.20889 | 432.20801 | -2.04 | 1 | 1023800.063 | 0.8 | Medium |
| AVGGE | 5 | | 432.20889 | 432.20801 | -2.04 | 1 | 1023800.063 | 0.8 | Medium |
| GIGGE | 5 | | 432.20889 | 432.20801 | -2.04 | 1 | 1023800.063 | 0.8 | Medium |
| ISIESATVAG | 10 | | 947.5044 | 474.25366 | -4.6 | 2 | | 0.8 | Medium |
| TDIE | 4 | | 477.21912 | 477.21823 | -1.86 | 1 | 481512.25 | 0.8 | Medium |
| SEIE | 4 | | 477.21912 | 477.21823 | -1.86 | 1 | 481512.25 | 0.8 | Medium |
| DTIE | 4 | | 477.21912 | 477.21823 | -1.86 | 1 | 481512.25 | 0.8 | Medium |
| ESIE | 4 | | 477.21912 | 477.21823 | -1.86 | 1 | 481512.25 | 0.8 | Medium |
| NVTDK | 5 | | 576.29877 | 576.29742 | -2.33 | 1 | 31486.83594 | 0.8 | Medium |
| VESDE | 5 | | 578.23041 | 578.22925 | -2.01 | 1 | 52454.04297 | 0.79 | Medium |
| IDSDE | 5 | | 578.23041 | 578.22925 | -2.01 | 1 | 52454.04297 | 0.79 | Medium |
| DISDE | 5 | | 578.23041 | 578.22925 | -2.01 | 1 | 52454.04297 | 0.79 | Medium |
| VSVE | 4 | | 433.22929 | 433.22815 | -2.63 | 1 | 126780.6406 | 0.79 | Medium |
| SVVE | 4 | | 433.22929 | 433.22815 | -2.63 | 1 | 126780.6406 | 0.79 | Medium |
| AEVE | 4 | | 447.20855 | 447.20746 | -2.45 | 1 | 652580.5625 | 0.79 | Medium |
| EAVE | 4 | | 447.20855 | 447.20746 | -2.45 | 1 | 815338.4531 | 0.79 | Medium |
| ESVE | 4 | | 463.20347 | 463.20282 | -1.4 | 1 | 418686.5 | 0.79 | Medium |
| TDVE | 4 | | 463.20347 | 463.20282 | -1.4 | 1 | 271670.9688 | 0.79 | Medium |
| DTVE | 4 | | 463.20347 | 463.20282 | -1.4 | 1 | 271670.9688 | 0.79 | Medium |
| SEVE | 4 | | 463.20347 | 463.20282 | -1.4 | 1 | 271670.9688 | 0.79 | Medium |
| WMPQ | 4 | | 561.24898 | 561.25055 | 2.8 | 1 | | 0.79 | Medium |
| NEIE | 4 | | 504.23002 | 504.22861 | -2.8 | 1 | 175137.6211 | 0.79 | Medium |
| DQIE | 4 | | 504.23002 | 504.22861 | -2.8 | 1 | 175137.6211 | 0.79 | Medium |
| GGEIE | 5 | | 504.23002 | 504.22861 | -2.8 | 1 | 75116.96484 | 0.79 | Medium |
| DDQIE | 5 | | 619.25696 | 619.25562 | -2.17 | 1 | 183570.8594 | 0.79 | Medium |
| EIAE | 4 | | 461.2242 | 461.22324 | -2.1 | 1 | 539275.2695 | 0.79 | Medium |
| EGVP | 4 | | 401.20308 | 401.20215 | -2.31 | 1 | 142372.9883 | 0.79 | Medium |
| EGPV | 4 | | 401.20308 | 401.20215 | -2.31 | 1 | 142372.9883 | 0.79 | Medium |
| DDAGIE | 6 | | 619.25696 | 619.25562 | -2.17 | 1 | 183570.8594 | 0.79 | Medium |
| PAGEDVD | 7 | | 702.29408 | 702.29333 | -1.05 | 1 | 44398.01563 | 0.79 | Medium |
| ADSASL | 6 | | 563.26713 | 563.26605 | -1.92 | 1 | | 0.79 | Medium |
| DVVE | 4 | | 461.2242 | 461.22336 | -1.84 | 1 | 118640.8125 | 0.79 | Medium |
| VDVE | 4 | | 461.2242 | 461.22336 | -1.84 | 1 | 118640.8125 | 0.79 | Medium |
| GDSIT | 5 | | 492.23002 | 492.22888 | -2.31 | 1 | 473339.4688 | 0.79 | Medium |
| DGSIT | 5 | | 492.23002 | 492.22888 | -2.31 | 1 | 473339.4688 | 0.79 | Medium |
| IAAE | 4 | | 403.21873 | 403.21777 | -2.36 | 1 | 214286.1563 | 0.79 | Medium |
| AIAE | 4 | | 403.21873 | 403.21777 | -2.36 | 1 | 214286.1563 | 0.79 | Medium |
| GAETPG | 6 | | 531.24092 | 531.23956 | -2.55 | 1 | 292808.5 | 0.79 | Medium |
| QETPG | 5 | | 531.24092 | 531.23956 | -2.55 | 1 | 292808.5 | 0.79 | Medium |
| DSIT | 4 | | 435.20855 | 435.20758 | -2.24 | 1 | 220979.0156 | 0.79 | Medium |
| SDIT | 4 | | 435.20855 | 435.20758 | -2.24 | 1 | 220979.0156 | 0.79 | Medium |
| GGTLE | 5 | | 476.2351 | 476.23438 | -1.53 | 1 | 113708.4609 | 0.79 | Medium |
| GSAIE | 5 | | 476.2351 | 476.23438 | -1.53 | 1 | 113708.4609 | 0.79 | Medium |
| SGALE | 5 | | 476.2351 | 476.23438 | -1.53 | 1 | 113708.4609 | 0.79 | Medium |
| EVSDE | 5 | | 578.23041 | 578.22925 | -2.01 | 1 | 52454.04297 | 0.79 | Medium |
| VIDE | 4 | | 475.23986 | 475.23889 | -2.03 | 1 | 505516.625 | 0.79 | Medium |
| IVDE | 4 | | 475.23986 | 475.23889 | -2.03 | 1 | 505516.625 | 0.79 | Medium |
| DGSDEV | 6 | | 621.23623 | 621.2348 | -2.29 | 1 | | 0.79 | Medium |
| GAGEL | 5 | | 446.22454 | 446.22302 | -3.4 | 1 | 11295279 | 0.79 | Medium |
| AGGEI | 5 | | 446.22454 | 446.22302 | -3.4 | 1 | 11295279 | 0.79 | Medium |
| QGEI | 4 | | 446.22454 | 446.22302 | -3.4 | 1 | 11295279 | 0.79 | Medium |
| DGSDVE | 6 | | 621.23623 | 621.2348 | -2.29 | 1 | | 0.79 | Medium |
| EQTGP | 5 | | 531.24092 | 531.23956 | -2.55 | 1 | 292808.5 | 0.79 | Medium |
| EQTPG | 5 | | 531.24092 | 531.23956 | -2.55 | 1 | 292808.5 | 0.79 | Medium |
| DGAIE | 5 | | 504.23002 | 504.22855 | -2.92 | 1 | 79241.93359 | 0.79 | Medium |
| GDALE | 5 | | 504.23002 | 504.22855 | -2.92 | 1 | 179262.5898 | 0.79 | Medium |
| AITE | 4 | | 433.22929 | 433.22827 | -2.35 | 1 | 137101.5508 | 0.79 | Medium |
| IATE | 4 | | 433.22929 | 433.22827 | -2.35 | 1 | 137101.5508 | 0.79 | Medium |
| AEDI | 4 | | 447.20855 | 447.20749 | -2.38 | 1 | 1491482.5 | 0.79 | Medium |
| AVDE | 4 | | 433.1929 | 433.19147 | -3.32 | 1 | 58632568 | 0.79 | Medium |
| VADE | 4 | | 433.1929 | 433.19147 | -3.32 | 1 | 58632568 | 0.79 | Medium |
| GIDE | 4 | | 433.1929 | 433.19147 | -3.32 | 1 | 58632568 | 0.79 | Medium |
| IGDE | 4 | | 433.1929 | 433.19147 | -3.32 | 1 | 58632568 | 0.79 | Medium |

| | | | | | | | | | |
|---|---|---|---|---|---|---|---|---|---|
| ESDYE | 5 | | 642.22533 | 642.22375 | -2.45 | 1 | 182812.3594 | 0.79 | Medium |
| AGGLE | 5 | | 446.22454 | 446.22302 | -3.4 | 1 | 11295279 | 0.79 | Medium |
| QGIE | 4 | | 446.22454 | 446.22302 | -3.4 | 1 | 11295279 | 0.79 | Medium |
| GAGIE | 5 | | 446.22454 | 446.22302 | -3.4 | 1 | 11295279 | 0.79 | Medium |
| AEID | 4 | | 447.20855 | 447.20746 | -2.45 | 1 | 652580.5625 | 0.79 | Medium |
| EAID | 4 | | 447.20855 | 447.20746 | -2.45 | 1 | 652580.5625 | 0.79 | Medium |
| GDPEAE | 6 | | 617.24131 | 617.23969 | -2.64 | 1 | 431264.0625 | 0.79 | Medium |
| DENVQ | 5 | | 604.2573 | 604.25641 | -1.47 | 1 | 2080173.828 | 0.79 | Medium |
| SIDDE | 5 | | 578.23041 | 578.22925 | -2.01 | 1 | 52454.04297 | 0.79 | Medium |
| ISDDE | 5 | | 578.23041 | 578.22925 | -2.01 | 1 | 52454.04297 | 0.79 | Medium |
| EGHCR | 5 | | 601.25111 | 601.25598 | 8.11 | 1 | 935708.25 | 0.79 | Medium |
| GEME | 4 | | 465.16498 | 465.164 | -2.09 | 1 | 2933245.656 | 0.79 | Medium |
| DAME | 4 | | 465.16498 | 465.164 | -2.09 | 1 | 2933245.656 | 0.79 | Medium |
| ADME | 4 | | 465.16498 | 465.164 | -2.09 | 1 | 2933245.656 | 0.79 | Medium |
| EDVNQ | 5 | | 604.2573 | 604.25629 | -1.67 | 1 | 2027437.125 | 0.79 | Medium |
| TESI | 4 | | 449.2242 | 449.22318 | -2.29 | 1 | 653791.3125 | 0.79 | Medium |
| TEIQ | 4 | | 490.25075 | 490.24939 | -2.78 | 1 | | 0.79 | Medium |
| TEIGA | 5 | | 490.25075 | 490.24939 | -2.78 | 1 | | 0.79 | Medium |
| TEIAG | 5 | | 490.25075 | 490.24939 | -2.78 | 1 | | 0.79 | Medium |
| ETSI | 4 | | 449.2242 | 449.22318 | -2.29 | 1 | 653791.3125 | 0.79 | Medium |
| SVGAE | 5 | | 462.21945 | 462.21863 | -1.79 | 1 | 141179.8281 | 0.79 | Medium |
| AAGPM | 5 | 1xOxidation [M5] | 462.2017 | 462.20035 | -2.92 | 1 | 77852.78125 | 0.79 | Medium |
| AAPGM | 5 | 1xOxidation [M5] | 462.2017 | 462.20035 | -2.92 | 1 | 77852.78125 | 0.79 | Medium |
| EEIE | 4 | | 519.22968 | 519.22742 | -4.37 | 1 | 403773.7891 | 0.79 | Medium |
| EDITQ | 5 | | 605.2777 | 605.27716 | -0.89 | 1 | 60707.97656 | 0.78 | Medium |
| IAETE | 5 | | 562.27188 | 562.27167 | -0.38 | 1 | | 0.78 | Medium |
| AIETE | 5 | | 562.27188 | 562.27167 | -0.38 | 1 | | 0.78 | Medium |
| IDVE | 4 | | 475.23986 | 475.23865 | -2.54 | 1 | 963763.3203 | 0.78 | Medium |
| EVVE | 4 | | 475.23986 | 475.23865 | -2.54 | 1 | 1240947.164 | 0.78 | Medium |
| DIVE | 4 | | 475.23986 | 475.23865 | -2.54 | 1 | 963763.3203 | 0.78 | Medium |
| VEVE | 4 | | 475.23986 | 475.23865 | -2.54 | 1 | 963763.3203 | 0.78 | Medium |
| VASE | 4 | | 405.19799 | 405.19724 | -1.86 | 1 | 541778.5469 | 0.78 | Medium |
| AVSE | 4 | | 405.19799 | 405.19724 | -1.86 | 1 | 541778.5469 | 0.78 | Medium |
| IGSE | 4 | | 405.19799 | 405.19724 | -1.86 | 1 | 315673.125 | 0.78 | Medium |
| GISE | 4 | | 405.19799 | 405.19724 | -1.86 | 1 | 541778.5469 | 0.78 | Medium |
| EDAIN | 5 | | 561.25148 | 561.24994 | -2.75 | 1 | 427734.8906 | 0.78 | Medium |
| DEAIN | 5 | | 561.25148 | 561.24994 | -2.75 | 1 | 282226.3438 | 0.78 | Medium |
| MTAE | 4 | | 451.18571 | 451.18466 | -2.33 | 1 | 261267.5781 | 0.78 | Medium |
| TMAE | 4 | | 451.18571 | 451.18466 | -2.33 | 1 | 261267.5781 | 0.78 | Medium |
| DADTM | 5 | 1xOxidation [M5] | 568.19192 | 568.19061 | -2.3 | 1 | 199207.1406 | 0.78 | Medium |
| SVEPE | 5 | | 560.25623 | 560.25494 | -2.3 | 1 | 2797302.793 | 0.78 | Medium |
| VSEPE | 5 | | 560.25623 | 560.25494 | -2.3 | 1 | 383437.4063 | 0.78 | Medium |
| DTDYE | 5 | | 642.22533 | 642.22382 | -2.35 | 1 | 182812.3594 | 0.78 | Medium |
| ETSLQ | 5 | | 577.28278 | 577.28131 | -2.55 | 1 | 103142.8047 | 0.78 | Medium |
| EDGTM | 5 | 1xOxidation [M5] | 568.19192 | 568.19049 | -2.51 | 1 | 72487.28125 | 0.78 | Medium |
| DEGTM | 5 | 1xOxidation [M5] | 568.19192 | 568.19049 | -2.51 | 1 | 72487.28125 | 0.78 | Medium |
| GEAGEV | 6 | | 561.25148 | 561.25055 | -1.66 | 1 | 558846.5625 | 0.77 | Medium |
| QEAVDSG | 7 | | 705.30497 | 705.30371 | -1.79 | 1 | 631444.8125 | 0.77 | Medium |
| EGSHAT | 6 | | 601.25763 | 601.25598 | -2.74 | 1 | 935708.25 | 0.77 | Medium |
| EQAEQT | 6 | | 705.30497 | 705.30371 | -1.79 | 1 | 631444.8125 | 0.77 | Medium |
| QEAQTE | 6 | | 705.30497 | 705.30371 | -1.79 | 1 | 631444.8125 | 0.77 | Medium |
| VEAT | 4 | | 419.21364 | 419.21277 | -2.08 | 1 | 2648089 | 0.77 | Medium |
| IDAT | 4 | | 419.21364 | 419.21277 | -2.08 | 1 | 2648089 | 0.77 | Medium |
| GNDY | 4 | | 468.1725 | 468.1716 | -1.93 | 1 | 66747.25 | 0.77 | Medium |
| NGDY | 4 | | 468.1725 | 468.1716 | -1.93 | 1 | 66747.25 | 0.77 | Medium |
| GGGDY | 5 | | 468.1725 | 468.1716 | -1.93 | 1 | 66747.25 | 0.77 | Medium |
| ISDGN | 5 | | 505.22527 | 505.224 | -2.51 | 1 | 478301.75 | 0.77 | Medium |
| TVDGN | 5 | | 505.22527 | 505.224 | -2.51 | 1 | 478301.75 | 0.77 | Medium |
| SIDGGG | 6 | | 505.22527 | 505.224 | -2.51 | 1 | 478301.75 | 0.77 | Medium |
| SIDGN | 5 | | 505.22527 | 505.224 | -2.51 | 1 | 478301.75 | 0.77 | Medium |
| PGSTQ | 5 | | 489.23035 | 489.22922 | -2.32 | 1 | 659644.7656 | 0.77 | Medium |
| DSAI | 4 | | 405.19799 | 405.19711 | -2.16 | 1 | 344760.6816 | 0.76 | Medium |
| SDAI | 4 | | 405.19799 | 405.19711 | -2.16 | 1 | 344760.6816 | 0.76 | Medium |
| IAEGA | 5 | | 460.24019 | 460.23932 | -1.89 | 1 | 712078.6875 | 0.76 | Medium |
| AIEQ | 4 | | 460.24019 | 460.23932 | -1.89 | 1 | 712078.6875 | 0.76 | Medium |
| IAEQ | 4 | | 460.24019 | 460.23932 | -1.89 | 1 | 712078.6875 | 0.76 | Medium |
| IAEAG | 5 | | 460.24019 | 460.23932 | -1.89 | 1 | 712078.6875 | 0.76 | Medium |
| ALEGA | 5 | | 460.24019 | 460.23932 | -1.89 | 1 | 712078.6875 | 0.76 | Medium |
| AIEAG | 5 | | 460.24019 | 460.23932 | -1.89 | 1 | 712078.6875 | 0.76 | Medium |
| GIEPN | 5 | | 529.26165 | 529.26038 | -2.41 | 1 | 63014.35547 | 0.76 | Medium |
| VAEPN | 5 | | 529.26165 | 529.26038 | -2.41 | 1 | 63014.35547 | 0.76 | Medium |
| AVEPN | 5 | | 529.26165 | 529.26038 | -2.41 | 1 | 63014.35547 | 0.76 | Medium |
| GLEPGG | 6 | | 529.26165 | 529.26038 | -2.41 | 1 | 63014.35547 | 0.76 | Medium |
| AGGEV | 5 | | 432.20889 | 432.2077 | -2.75 | 1 | 313861.6875 | 0.76 | Medium |
| AGGVE | 5 | | 432.20889 | 432.2077 | -2.75 | 1 | 340946.998 | 0.76 | Medium |
| GAGVE | 5 | | 432.20889 | 432.2077 | -2.75 | 1 | 313861.6875 | 0.76 | Medium |
| QGVE | 4 | | 432.20889 | 432.2077 | -2.75 | 1 | 340946.998 | 0.76 | Medium |
| QGEV | 4 | | 432.20889 | 432.2077 | -2.75 | 1 | 313861.6875 | 0.76 | Medium |
| TEPTDAAS | 8 | | 791.34175 | 791.34039 | -1.72 | 1 | 49879.03516 | 0.76 | Medium |
| QGDI | 4 | | 432.20889 | 432.2077 | -2.75 | 1 | 313861.6875 | 0.76 | Medium |
| AGGID | 5 | | 432.20889 | 432.2077 | -2.75 | 1 | 313861.6875 | 0.76 | Medium |
| GAGDI | 5 | | 432.20889 | 432.2077 | -2.75 | 1 | 313861.6875 | 0.76 | Medium |
| QGLD | 4 | | 432.20889 | 432.2077 | -2.75 | 1 | 313861.6875 | 0.76 | Medium |

| | | | | | | | | | | |
|---|---|---|---|---|---|---|---|---|---|---|
| DSLLGDE | 7 | | 748.33594 | 748.33453 | -1.88 | 1 | 669942.8125 | 0.76 | Medium |
| ASEI | 4 | | 419.21364 | 419.21271 | -2.23 | 1 | 3430952.016 | 0.76 | Medium |
| YDESE | 5 | | 642.22533 | 642.22382 | -2.35 | 1 | 182812.3594 | 0.76 | Medium |
| STAVE | 5 | | 506.24567 | 506.24454 | -2.23 | 1 | 362564.1172 | 0.76 | Medium |
| TSAVE | 5 | | 506.24567 | 506.24454 | -2.23 | 1 | 285901.625 | 0.76 | Medium |
| ADVVE | 5 | | 532.26132 | 532.26025 | -2 | 1 | | 0.76 | Medium |
| GEVVE | 5 | | 532.26132 | 532.26025 | -2 | 1 | | 0.76 | Medium |
| EGVVE | 5 | | 532.26132 | 532.26025 | -2 | 1 | | 0.76 | Medium |
| DAVVE | 5 | | 532.26132 | 532.26025 | -2 | 1 | | 0.76 | Medium |
| EGAEAPN | 7 | | 687.29441 | 687.2934 | -1.47 | 1 | 226685.2969 | 0.76 | Medium |
| DDSI | 4 | | 449.18782 | 449.18692 | -2 | 1 | 155126.0781 | 0.76 | Medium |
| NQGTQAM | 7 | | 749.32466 | 749.32239 | -3.04 | 1 | 25152.02148 | 0.75 | Medium |
| IEAE | 4 | | 461.2242 | 461.22302 | -2.56 | 1 | 456462.8398 | 0.75 | Medium |
| ETAIE | 5 | | 562.27188 | 562.27167 | -0.38 | 1 | | 0.75 | Medium |
| TEAIE | 5 | | 562.27188 | 562.27167 | -0.38 | 1 | | 0.75 | Medium |
| IVSGG | 5 | | 432.24527 | 432.24414 | -2.62 | 1 | 91169.95313 | 0.75 | Medium |
| VISN | 4 | | 432.24527 | 432.24414 | -2.62 | 1 | 91169.95313 | 0.75 | Medium |
| VISGG | 5 | | 432.24527 | 432.24414 | -2.62 | 1 | 91169.95313 | 0.75 | Medium |
| IVSN | 4 | | 432.24527 | 432.24414 | -2.62 | 1 | 91169.95313 | 0.75 | Medium |
| IETT | 4 | | 463.23986 | 463.23892 | -2.01 | 1 | 60569.46875 | 0.75 | Medium |
| EITT | 4 | | 463.23986 | 463.23892 | -2.01 | 1 | 60569.46875 | 0.75 | Medium |
| PDNPN | 5 | | 556.23617 | 556.23474 | -2.56 | 1 | 49182.07813 | 0.75 | Medium |
| DSQIN | 5 | | 576.26238 | 576.26172 | -1.15 | 1 | 56710.05859 | 0.75 | Medium |
| SDQIN | 5 | | 576.26238 | 576.26172 | -1.15 | 1 | 56710.05859 | 0.75 | Medium |
| AQTPE | 5 | | 545.25657 | 545.25635 | -0.4 | 1 | 54308.74219 | 0.75 | Medium |
| GSSIQD | 6 | | 606.27295 | 606.27069 | -3.72 | 1 | 209628.5 | 0.75 | Medium |
| DSAGIN | 6 | | 576.26238 | 576.26172 | -1.15 | 1 | 56710.05859 | 0.75 | Medium |
| DSGAIGG | 7 | | 576.26238 | 576.26172 | -1.15 | 1 | 56710.05859 | 0.75 | Medium |
| AQTEP | 5 | | 545.25657 | 545.25635 | -0.4 | 1 | 54308.74219 | 0.74 | Medium |
| EMLHGE | 6 | | 715.30795 | 715.31183 | 5.42 | 1 | 196920.2031 | 0.74 | Medium |
| TAIE | 4 | | 433.22929 | 433.22809 | -2.77 | 1 | 224962 | 0.74 | Medium |
| ATIE | 4 | | 433.22929 | 433.22809 | -2.77 | 1 | 276032.0039 | 0.74 | Medium |
| GSSLEN | 6 | | 606.27295 | 606.27069 | -3.72 | 1 | 209628.5 | 0.74 | Medium |
| SSPVE | 5 | | 518.24567 | 518.24438 | -2.48 | 1 | | 0.74 | Medium |
| AEDLKE | 6 | | 704.34611 | 704.34222 | -5.52 | 1 | 56395.08984 | 0.74 | Medium |
| AAGSGDME | 8 | | 737.27705 | 737.27582 | -1.66 | 1 | 89637.32813 | 0.74 | Medium |
| GEPTEQ | 6 | | 660.28351 | 660.28107 | -3.7 | 1 | 50919.70313 | 0.74 | Medium |
| SGSIDQ | 6 | | 606.27295 | 606.27069 | -3.72 | 1 | 209628.5 | 0.74 | Medium |
| MSEPT | 5 | | 564.23339 | 564.23248 | -1.61 | 1 | 56863.43359 | 0.74 | Medium |
| MAEPT | 5 | 1xOxidation [M1] | 564.23339 | 564.23248 | -1.61 | 1 | 56863.43359 | 0.74 | Medium |
| VEPAG | 5 | | 472.24019 | 472.23926 | -1.97 | 1 | 128770.5469 | 0.74 | Medium |
| VEPGA | 5 | | 472.24019 | 472.23926 | -1.97 | 1 | 128770.5469 | 0.74 | Medium |
| VEPQ | 4 | | 472.24019 | 472.23926 | -1.97 | 1 | 128770.5469 | 0.74 | Medium |
| ETAV | 4 | | 419.21364 | 419.21277 | -2.08 | 1 | 2753301 | 0.74 | Medium |
| TEAV | 4 | | 419.21364 | 419.21277 | -2.08 | 1 | 2753301 | 0.74 | Medium |
| VVAE | 4 | | 417.23438 | 417.23337 | -2.42 | 1 | 561181.75 | 0.74 | Medium |
| NDGNLA | 6 | | 603.27328 | 603.27191 | -2.27 | 1 | 113294.0391 | 0.74 | Medium |
| PEFPGA | 6 | | 617.29295 | 617.28748 | -8.87 | 1 | 215856.2656 | 0.74 | Medium |
| AEDIEK | 6 | | 704.34611 | 704.34222 | -5.52 | 1 | 56395.08984 | 0.74 | Medium |
| EADLLSG | 7 | | 704.34611 | 704.34222 | -5.52 | 1 | 56395.08984 | 0.74 | Medium |
| EVGPG | 5 | | 458.22454 | 458.2236 | -2.04 | 1 | 854207.6094 | 0.74 | Medium |
| EVGGP | 5 | | 458.22454 | 458.2236 | -2.04 | 1 | 854207.6094 | 0.74 | Medium |
| EADLSAV | 7 | | 704.34611 | 704.34222 | -5.52 | 1 | 56395.08984 | 0.74 | Medium |
| QTIE | 4 | | 490.25075 | 490.24969 | -2.16 | 1 | 62917.98828 | 0.74 | Medium |
| AGTIE | 5 | | 490.25075 | 490.24969 | -2.16 | 1 | 62917.98828 | 0.74 | Medium |
| TQIE | 4 | | 490.25075 | 490.24969 | -2.16 | 1 | 62917.98828 | 0.74 | Medium |
| GATLE | 5 | | 490.25075 | 490.24969 | -2.16 | 1 | 62917.98828 | 0.74 | Medium |
| VGGEGDNQ | 8 | | 775.32169 | 775.32056 | -1.46 | 1 | 96650.53125 | 0.74 | Medium |
| SAAIE | 5 | | 490.25075 | 490.24969 | -2.16 | 1 | 453357.5508 | 0.74 | Medium |
| TGAIE | 5 | | 490.25075 | 490.24969 | -2.16 | 1 | 453357.5508 | 0.74 | Medium |
| ASAIE | 5 | | 490.25075 | 490.24969 | -2.16 | 1 | 453357.5508 | 0.74 | Medium |
| DEIE | 4 | | 505.21403 | 505.21268 | -2.69 | 1 | 255417.6641 | 0.74 | Medium |
| SPGSTTG | 7 | | 606.27295 | 606.27173 | -2.01 | 1 | 229950.875 | 0.74 | Medium |
| EGGTM | 5 | 1xOxidation [M5] | 510.18644 | 510.18491 | -3.01 | 1 | 67376.625 | 0.74 | Medium |
| GEGTM | 5 | 1xOxidation [M5] | 510.18644 | 510.18491 | -3.01 | 1 | 67376.625 | 0.74 | Medium |
| QSSM | 4 | | 452.18096 | 452.1795 | -3.22 | 1 | 186711.4375 | 0.73 | Medium |
| GASSM | 5 | | 452.18096 | 452.1795 | -3.22 | 1 | 186711.4375 | 0.73 | Medium |
| IEAGG | 5 | | 446.22454 | 446.22388 | -1.48 | 1 | 93532.88281 | 0.73 | Medium |
| EIAGG | 5 | | 446.22454 | 446.22388 | -1.48 | 1 | 93532.88281 | 0.73 | Medium |
| IEAN | 4 | | 446.22454 | 446.22388 | -1.48 | 1 | 93532.88281 | 0.73 | Medium |
| EIAN | 4 | | 446.22454 | 446.22388 | -1.48 | 1 | 93532.88281 | 0.73 | Medium |
| VDGCAA | 6 | | 535.21807 | 535.21643 | -3.07 | 1 | 19616012 | 0.73 | Medium |
| VEEAT | 5 | | 548.25623 | 548.25507 | -2.13 | 1 | 126626.25 | 0.73 | Medium |
| LDEAT | 5 | | 548.25623 | 548.25507 | -2.13 | 1 | 126626.25 | 0.73 | Medium |
| EVEAT | 5 | | 548.25623 | 548.25507 | -2.13 | 1 | 126626.25 | 0.73 | Medium |
| DIEAT | 5 | | 548.25623 | 548.25507 | -2.13 | 1 | 126626.25 | 0.73 | Medium |
| DVGMN | 5 | | 535.21807 | 535.21643 | -3.07 | 1 | 19616012 | 0.73 | Medium |
| VDGMN | 5 | | 535.21807 | 535.21643 | -3.07 | 1 | 19616012 | 0.73 | Medium |
| ADEM | 4 | | 465.16498 | 465.16385 | -2.42 | 1 | 2610868.5 | 0.73 | Medium |
| GEEM | 4 | | 465.16498 | 465.16385 | -2.42 | 1 | 2610868.5 | 0.73 | Medium |
| DAEM | 4 | | 465.16498 | 465.16385 | -2.42 | 1 | 2610868.5 | 0.73 | Medium |
| EGEM | 4 | | 465.16498 | 465.16385 | -2.42 | 1 | 2610868.5 | 0.73 | Medium |
| EGHCAE | 6 | | 645.2297 | 645.23456 | 7.53 | 1 | 1925368.125 | 0.73 | Medium |

| | | | | | | | | | | |
|---|---|---|---|---|---|---|---|---|---|---|
| ISPE | 4 | | 445.22929 | 445.22836 | -2.08 | 1 | 848338 | 0.73 | Medium |
| TVPE | 4 | | 445.22929 | 445.22836 | -2.08 | 1 | 848338 | 0.73 | Medium |
| SIPE | 4 | | 445.22929 | 445.22836 | -2.08 | 1 | 848338 | 0.73 | Medium |
| VTPE | 4 | | 445.22929 | 445.22836 | -2.08 | 1 | 848338 | 0.73 | Medium |
| TEAPE | 5 | | 546.24058 | 546.23895 | -2.99 | 1 | 193999.8516 | 0.72 | Medium |
| ETAPE | 5 | | 546.24058 | 546.23895 | -2.99 | 1 | 193999.8516 | 0.72 | Medium |
| ASIE | 4 | | 419.21364 | 419.21265 | -2.37 | 1 | 6113879.531 | 0.72 | Medium |
| IESET | 5 | | 578.2668 | 578.26605 | -1.29 | 1 | 458752.9844 | 0.72 | Medium |
| EISET | 5 | | 578.2668 | 578.26605 | -1.29 | 1 | 233112.9219 | 0.72 | Medium |
| DGSIDD | 6 | | 621.23623 | 621.23492 | -2.1 | 1 | | 0.72 | Medium |
| GGGGETL | 7 | | 590.27803 | 590.2771 | -1.58 | 1 | 68722.10156 | 0.72 | Medium |
| GNGEIT | 6 | | 590.27803 | 590.2771 | -1.58 | 1 | 68722.10156 | 0.72 | Medium |
| ITDE | 4 | | 477.21912 | 477.21768 | -3.01 | 1 | 206502.3984 | 0.72 | Medium |
| TIDE | 4 | | 477.21912 | 477.21768 | -3.01 | 1 | 206502.3984 | 0.72 | Medium |
| SAEIA | 5 | | 490.25075 | 490.24969 | -2.16 | 1 | 390439.5625 | 0.72 | Medium |
| ASEIA | 5 | | 490.25075 | 490.24969 | -2.16 | 1 | 390439.5625 | 0.72 | Medium |
| GTEIA | 5 | | 490.25075 | 490.24969 | -2.16 | 1 | 390439.5625 | 0.72 | Medium |
| TGEIA | 5 | | 490.25075 | 490.24969 | -2.16 | 1 | 390439.5625 | 0.72 | Medium |
| DYVE | 4 | | 525.21912 | 525.21887 | -0.47 | 1 | 49188.95313 | 0.72 | Medium |
| DEGI | 4 | | 433.1929 | 433.19162 | -2.97 | 1 | 151775.0625 | 0.72 | Medium |
| YDVE | 4 | | 525.21912 | 525.21887 | -0.47 | 1 | 49188.95313 | 0.72 | Medium |
| SAEAI | 5 | | 490.25075 | 490.24969 | -2.16 | 1 | 390439.5625 | 0.72 | Medium |
| GIPE | 4 | | 415.21873 | 415.21765 | -2.59 | 1 | 1389837.25 | 0.72 | Medium |
| AVPE | 4 | | 415.21873 | 415.21765 | -2.59 | 1 | 1389837.25 | 0.72 | Medium |
| IGPE | 4 | | 415.21873 | 415.21765 | -2.59 | 1 | 1389837.25 | 0.72 | Medium |
| EDGI | 4 | | 433.1929 | 433.19162 | -2.97 | 1 | 58784343.06 | 0.72 | Medium |
| GTGLE | 5 | | 476.2351 | 476.23373 | -2.88 | 1 | 35920.52734 | 0.72 | Medium |
| ASGIE | 5 | | 476.2351 | 476.23373 | -2.88 | 1 | 35920.52734 | 0.72 | Medium |
| SAEAV | 5 | | 476.2351 | 476.23407 | -2.17 | 1 | 659074.75 | 0.72 | Medium |
| GTEIG | 5 | | 476.2351 | 476.23407 | -2.17 | 1 | 1098544.156 | 0.72 | Medium |
| ASEIG | 5 | | 476.2351 | 476.23407 | -2.17 | 1 | 659074.75 | 0.72 | Medium |
| ASEAV | 5 | | 476.2351 | 476.23407 | -2.17 | 1 | 659074.75 | 0.72 | Medium |
| SAEIG | 5 | | 476.2351 | 476.23407 | -2.17 | 1 | 659074.75 | 0.72 | Medium |
| TGEIG | 5 | | 476.2351 | 476.23407 | -2.17 | 1 | 1098544.156 | 0.72 | Medium |
| ADNLD | 5 | | 547.23583 | 547.2345 | -2.44 | 1 | 38555.01172 | 0.72 | Medium |
| EGNVE | 5 | | 547.23583 | 547.2345 | -2.44 | 1 | 38555.01172 | 0.72 | Medium |
| GENEV | 5 | | 547.23583 | 547.2345 | -2.44 | 1 | 38555.01172 | 0.72 | Medium |
| EGNLD | 5 | | 547.23583 | 547.2345 | -2.44 | 1 | 38555.01172 | 0.72 | Medium |
| GENID | 5 | | 547.23583 | 547.2345 | -2.44 | 1 | 38555.01172 | 0.72 | Medium |
| GENDL | 5 | | 547.23583 | 547.2345 | -2.44 | 1 | 38555.01172 | 0.72 | Medium |
| DANLD | 5 | | 547.23583 | 547.2345 | -2.44 | 1 | 38555.01172 | 0.72 | Medium |
| EGNDI | 5 | | 547.23583 | 547.2345 | -2.44 | 1 | 38555.01172 | 0.72 | Medium |
| LGDQ | 4 | | 432.20889 | 432.20773 | -2.67 | 1 | 1023800.063 | 0.72 | Medium |
| AVDAG | 5 | | 432.20889 | 432.20773 | -2.67 | 1 | 1023800.063 | 0.72 | Medium |
| VADQ | 4 | | 432.20889 | 432.20773 | -2.67 | 1 | 1023800.063 | 0.72 | Medium |
| IGDGA | 5 | | 432.20889 | 432.20773 | -2.67 | 1 | 1023800.063 | 0.72 | Medium |
| GIDAG | 5 | | 432.20889 | 432.20773 | -2.67 | 1 | 1023800.063 | 0.72 | Medium |
| AVDQ | 4 | | 432.20889 | 432.20773 | -2.67 | 1 | 1023800.063 | 0.72 | Medium |
| LGDAG | 5 | | 432.20889 | 432.20773 | -2.67 | 1 | 1023800.063 | 0.72 | Medium |
| GIDQ | 4 | | 432.20889 | 432.20773 | -2.67 | 1 | 1023800.063 | 0.72 | Medium |
| GLDGA | 5 | | 432.20889 | 432.20773 | -2.67 | 1 | 1023800.063 | 0.72 | Medium |
| AMGE | 4 | | 407.1595 | 407.15839 | -2.73 | 1 | 149350.5938 | 0.71 | Medium |
| CVGE | 4 | | 407.1595 | 407.15839 | -2.73 | 1 | 149350.5938 | 0.71 | Medium |
| VCGE | 4 | | 407.1595 | 407.15839 | -2.73 | 1 | 149350.5938 | 0.71 | Medium |
| GTGPA | 5 | | 402.19832 | 402.19708 | -3.09 | 1 | 4397279 | 0.71 | Medium |
| GEVTTG | 6 | | 563.26713 | 563.26593 | -2.13 | 1 | 83845.58594 | 0.71 | Medium |
| ADVTSA | 6 | | 563.26713 | 563.26593 | -2.13 | 1 | 83845.58594 | 0.71 | Medium |
| FNDGG | 5 | | 509.19905 | 509.19781 | -2.43 | 1 | 154952.2188 | 0.71 | Medium |
| FNDN | 4 | | 509.19905 | 509.19781 | -2.43 | 1 | 154952.2188 | 0.71 | Medium |
| TDIET | 5 | | 578.2668 | 578.26605 | -1.29 | 1 | 233112.9219 | 0.71 | Medium |
| DTIET | 5 | | 578.2668 | 578.26605 | -1.29 | 1 | 233112.9219 | 0.71 | Medium |
| ESLET | 5 | | 578.2668 | 578.26605 | -1.29 | 1 | 233112.9219 | 0.71 | Medium |
| SELET | 5 | | 578.2668 | 578.26605 | -1.29 | 1 | 233112.9219 | 0.71 | Medium |
| GSSDI | 5 | | 478.21437 | 478.21304 | -2.77 | 1 | 1431645.125 | 0.7 | Medium |
| SGSVE | 5 | | 478.21437 | 478.21304 | -2.77 | 1 | 1431645.125 | 0.7 | Medium |
| GSSVE | 5 | | 478.21437 | 478.21304 | -2.77 | 1 | 1431645.125 | 0.7 | Medium |
| SGSDL | 5 | | 478.21437 | 478.21304 | -2.77 | 1 | 1431645.125 | 0.7 | Medium |
| IETE | 4 | | 491.23477 | 491.23386 | -1.86 | 1 | 583882.6484 | 0.7 | Medium |
| PGSNTT | 6 | | 576.26238 | 576.26068 | -2.95 | 1 | 2579463 | 0.7 | Medium |
| SAEI | 4 | | 419.21364 | 419.21265 | -2.37 | 1 | 712489.5313 | 0.7 | Medium |
| TGEL | 4 | | 419.21364 | 419.21265 | -2.37 | 1 | 782863.0156 | 0.7 | Medium |
| EVSAG | 5 | | 462.21945 | 462.21857 | -1.92 | 1 | 189392.9219 | 0.7 | Medium |
| EVSQ | 4 | | 462.21945 | 462.21857 | -1.92 | 1 | 189392.9219 | 0.7 | Medium |
| SGEAY | 5 | | 526.21437 | 526.21283 | -2.92 | 1 | 21577.15234 | 0.7 | Medium |
| EVSGA | 5 | | 462.21945 | 462.21857 | -1.92 | 1 | 189392.9219 | 0.7 | Medium |
| EGIGAD | 6 | | 561.25148 | 561.24994 | -2.75 | 1 | 558846.5625 | 0.7 | Medium |
| GELGAD | 6 | | 561.25148 | 561.24994 | -2.75 | 1 | 558846.5625 | 0.7 | Medium |
| DISDP | 5 | | 546.24058 | 546.23926 | -2.43 | 1 | 78806.92969 | 0.7 | Medium |
| IDSDP | 5 | | 546.24058 | 546.23926 | -2.43 | 1 | 78806.92969 | 0.7 | Medium |
| DISPD | 5 | | 546.24058 | 546.23926 | -2.43 | 1 | 78806.92969 | 0.7 | Medium |
| EVSPD | 5 | | 546.24058 | 546.23926 | -2.43 | 1 | 78806.92969 | 0.7 | Medium |
| LDSPD | 5 | | 546.24058 | 546.23926 | -2.43 | 1 | 78806.92969 | 0.7 | Medium |
| SAAEAQE | 7 | | 705.30497 | 705.30334 | -2.31 | 1 | 631444.8125 | 0.7 | Medium |

| | | | | | | | | | |
|---|---|---|---|---|---|---|---|---|---|
| MEETE | 5 | | 638.23378 | 638.23291 | -1.37 | 1 | 82184.75 | 0.7 | Medium |
| **Fraction no. 14** | | | | | | | | | |
| IPIN | 4 | | 456.28166 | 456.2804 | -2.77 | 1 | 9504470 | 1.1 | Medium |
| VPSV | 4 | | 401.23946 | 401.23837 | -2.71 | 1 | 54510248 | 0.9 | Medium |
| AAKDPSNFDPR | 11 | | 1217.59093 | 406.53409 | -2.64 | 3 | 2642089.75 | 1.57 | Medium |
| AAPTF | 5 | | 506.26092 | 506.25912 | -3.56 | 1 | 3227605.5 | 1.27 | High |
| ADANF | 5 | | 537.23035 | 537.22772 | -4.9 | 1 | 1004501.438 | 0.75 | Medium |
| ADDDVAL | 7 | | 718.32538 | 718.32233 | -4.24 | 1 | 607485.6875 | 1.05 | Medium |
| ADFR | 4 | | 508.25142 | 508.25031 | -2.2 | 1 | 205533.9453 | 0.71 | Medium |
| ADGMLE | 6 | | 635.2705 | 635.26898 | -2.39 | 1 | 3439213.5 | 1.1 | Medium |
| ADIIK | 5 | | 559.34499 | 559.34344 | -2.76 | 1 | 3278808.375 | 1.36 | High |
| ADIIS | 5 | | 518.28205 | 518.2807 | -2.61 | 1 | 8543845 | 0.83 | Medium |
| ADLTV | 5 | | 518.28205 | 518.2807 | -2.61 | 1 | 8543845 | 0.83 | Medium |
| ADMF | 4 | 1xOxidation [M3] | 499.18571 | 499.1846 | -2.22 | 1 | 5938820.5 | 0.84 | Medium |
| ADVEQLR | 7 | | 830.43666 | 415.72058 | -3.34 | 2 | 1748187.5 | 0.85 | Medium |
| AEAAALQAA | 9 | | 815.42576 | 815.42444 | -1.62 | 1 | 2238012 | 1.25 | High |
| AEAAPL | 6 | | 571.3086 | 571.30682 | -3.11 | 1 | 1250706 | 0.76 | Medium |
| AEAIP | 5 | | 500.27149 | 500.27014 | -2.69 | 1 | 6027148 | 1.03 | Medium |
| AEAPI | 5 | | 500.27149 | 500.27014 | -2.69 | 1 | 6027148 | 1.03 | Medium |
| AEYIENGAKLGWLIDPKNK | 19 | | 2159.14953 | 720.39172 | 5.14 | 3 | | 1.03 | Medium |
| AGAAF | 5 | | 436.21906 | 436.21741 | -3.79 | 1 | 1911378.125 | 1.06 | Medium |
| AGDIIE | 6 | | 617.31408 | 617.31274 | -2.17 | 1 | 796395.125 | 0.93 | Medium |
| AGEGF | 5 | | 480.20889 | 480.20782 | -2.22 | 1 | 12471353 | 0.74 | Medium |
| AGENF | 5 | | 537.23035 | 537.22772 | -4.9 | 1 | 1004501.438 | 0.75 | Medium |
| AGEPLE | 6 | | 615.29843 | 615.29736 | -1.74 | 1 | 168858.1875 | 0.77 | Medium |
| AGGLYE | 6 | | 609.28787 | 609.28705 | -1.34 | 1 | | 1.16 | Medium |
| AGIDADIR | 8 | | 830.43666 | 415.72058 | -3.34 | 2 | 1748187.5 | 0.87 | Medium |
| AGIDI | 5 | | 488.27149 | 488.27075 | -1.51 | 1 | 1568978.75 | 0.7 | Medium |
| AGIEGI | 6 | | 559.3086 | 559.30695 | -2.96 | 1 | 23551980 | 0.75 | Medium |
| AGIEIG | 6 | | 559.3086 | 559.30695 | -2.96 | 1 | 23551980 | 0.75 | Medium |
| AGIIE | 5 | | 502.28714 | 502.28705 | -0.18 | 1 | 1159842.25 | 0.99 | Medium |
| AGNAF | 5 | | 479.22487 | 479.22382 | -2.21 | 1 | 10988858 | 1.03 | Medium |
| AGNWE | 5 | | 576.24125 | 576.24023 | -1.77 | 1 | 1150539.875 | 0.85 | Medium |
| AGSGIY | 6 | | 567.2773 | 567.27582 | -2.62 | 1 | 913628.625 | 0.79 | Medium |
| AGTALP | 6 | | 529.29804 | 529.29645 | -3.01 | 1 | 1138546.875 | 0.75 | Medium |
| AHDF | 4 | | 489.20922 | 489.20789 | -2.73 | 1 | 332517 | 0.74 | Medium |
| AHTLTTACIL | 10 | | 1043.55539 | 522.28168 | 0.66 | 2 | 10550444 | 0.85 | Medium |
| AKRIIYN | 7 | | 877.52541 | 439.26483 | -3.45 | 2 | 1423605.75 | 1.82 | Medium |
| AIAIGK | 6 | | 572.37662 | 572.37518 | -2.52 | 1 | 1143380.25 | 1.82 | High |
| AIDSPKLH | 8 | | 880.48869 | 440.74695 | -2.35 | 2 | 1472831.75 | 1.35 | Medium |
| AIGLH | 5 | | 510.30346 | 510.30173 | -3.39 | 1 | 4903355.5 | 1.68 | High |
| AIGVTKTLN | 9 | | 916.54621 | 916.54437 | -2 | 1 | 948161.875 | 0.81 | Medium |
| AIIGG | 5 | | 430.26601 | 430.26477 | -2.88 | 1 | 48216220 | 0.94 | Medium |
| ALLKWLN | 7 | | 857.52435 | 429.26895 | 7.32 | 2 | 2859924.5 | 0.82 | Medium |
| ALIN | 4 | | 430.26601 | 430.26477 | -2.88 | 1 | 48216220 | 0.94 | Medium |
| AILYGRIN | 8 | | 919.53598 | 919.54401 | 8.73 | 1 | 1489829.25 | 1.05 | Medium |
| AIPIN | 5 | | 527.31877 | 527.31738 | -2.64 | 1 | 1921580.75 | 1.03 | Medium |
| ALPSLYR | 7 | | 819.47231 | 410.23889 | -2.21 | 2 | 282928.25 | 1.1 | Medium |
| AISDDITSK | 9 | | 949.48367 | 949.48346 | -0.22 | 1 | 23910600.5 | 2.97 | High |
| AIVDN | 5 | | 531.2773 | 531.27515 | -4.06 | 1 | 3529007.25 | 0.75 | Medium |
| ALVGDG | 6 | | 531.2773 | 531.27515 | -4.06 | 1 | 3529007.25 | 0.75 | Medium |
| AIVND | 5 | | 531.2773 | 531.27515 | -4.06 | 1 | 3529007.25 | 0.75 | Medium |
| AIVNDPA | 7 | | 699.36718 | 699.36505 | -3.04 | 1 | 257844472 | 1.09 | Medium |
| AIVPM | 5 | 1xOxidation [M5] | 546.2956 | 546.29376 | -3.36 | 1 | 2840759.25 | 1.06 | Medium |
| ANDFR | 5 | | 622.29435 | 311.64996 | -2.73 | 2 | 272638.2813 | 0.87 | Medium |
| ANDLP | 5 | | 529.26165 | 529.2608 | -1.61 | 1 | 3142693.75 | 1.09 | Medium |
| ANDPL | 5 | | 529.26165 | 529.2608 | -1.61 | 1 | 3142693.75 | 1.09 | Medium |
| ANFPNTE | 7 | | 792.35226 | 792.34985 | -3.04 | 1 | 3217419.5 | 0.97 | Medium |
| ANLYE | 5 | | 609.28787 | 609.28705 | -1.34 | 1 | | 1.16 | Medium |
| ANPVIE | 6 | | 642.34572 | 642.34265 | -4.77 | 1 | 2527219 | 0.97 | Medium |
| APGFGDR | 7 | | 719.34711 | 360.17642 | -2.15 | 2 | 3492133.75 | 1.55 | Medium |
| APGGQ | 5 | | 429.20922 | 429.20801 | -2.83 | 1 | 10736032 | 1.7 | High |
| APQVGY | 6 | | 634.3195 | 634.31781 | -2.67 | 1 | 7545662.5 | 1.1 | Medium |
| APTPPGYT | 8 | | 803.3934 | 803.39215 | -1.55 | 1 | 952121.5625 | 1.19 | Medium |
| AQAF | 4 | | 436.21906 | 436.21741 | -3.79 | 1 | 1911378.125 | 1.06 | Medium |
| AQPDLVRQ | 8 | | 926.50541 | 463.755 | -2.88 | 2 | 1588867 | 0.97 | Medium |
| AQPKLNQQ | 8 | | 926.50541 | 463.755 | -2.88 | 2 | 1588867 | 0.97 | Medium |
| ARVI | 4 | | 458.30854 | 229.65733 | -2.52 | 2 | 20562840.5 | 1.16 | Medium |
| ASAAIP | 6 | | 529.29804 | 529.29645 | -3.01 | 1 | 1138546.875 | 0.75 | Medium |
| ASAAPL | 6 | | 529.29804 | 529.29645 | -3.01 | 1 | 1138546.875 | 0.75 | Medium |
| ASGIY | 5 | | 510.25584 | 510.25339 | -4.81 | 1 | 1668234.125 | 0.87 | Medium |
| ASGSLP | 6 | | 531.2773 | 531.276 | -2.45 | 1 | 13971215 | 0.84 | Medium |
| ASTVPIGNLGH | 11 | | 1065.56873 | 533.2865 | -2.83 | 2 | 146211408 | 0.86 | Medium |
| ATATITINPVNDAP | 14 | | 1397.72708 | 1397.72253 | -3.26 | 1 | 1808993.625 | 0.75 | Medium |
| AVEPPSYS | 8 | | 849.39887 | 849.40295 | 4.8 | 1 | | 1.05 | Medium |
| AVKLGA | 6 | | 558.36097 | 558.35968 | -2.32 | 1 | 1115697 | 1.41 | High |
| AVID | 4 | | 417.23438 | 417.23325 | -2.71 | 1 | 29495884 | 0.88 | Medium |
| AVIK | 4 | | 430.3024 | 430.30127 | -2.62 | 1 | 1368010.625 | 0.84 | Medium |
| AVIT | 4 | | 403.25511 | 403.25415 | -2.38 | 1 | 964773.5 | 0.8 | Medium |
| AVIVM | 5 | 1xOxidation [M5] | 548.31125 | 548.31085 | -0.72 | 1 | 1267634.875 | 1.11 | Medium |
| AVPWFADGK | 9 | | 990.50434 | 495.75189 | -7.91 | 2 | 1287403 | 0.96 | Medium |
| AVYEGR | 6 | | 694.35187 | 347.67877 | -2.3 | 2 | 1121682.75 | 0.81 | Medium |
| AYVQ | 4 | | 480.24527 | 480.24368 | -3.31 | 1 | | 0.76 | Medium |

| | | | | | | | | | |
|---|---|---|---|---|---|---|---|---|---|
| CSNNYGPYH | 9 | | 1054.40471 | 527.7063 | 0.58 | 2 | 291693.7188 | 0.93 | Medium |
| CVAEPGLTS | 9 | | 876.41314 | 876.41943 | 7.18 | 1 | 1718222.625 | 1.09 | Medium |
| DAANF | 5 | | 537.23035 | 537.22772 | -4.9 | 1 | 1004501.438 | 0.75 | Medium |
| DADTLV | 6 | | 633.309 | 633.30695 | -3.24 | 1 | 1130173.375 | 0.82 | Medium |
| DAFR | 4 | | 508.25142 | 508.25031 | -2.2 | 1 | 205533.9453 | 0.71 | Medium |
| DAIIK | 5 | | 559.34499 | 559.34344 | -2.76 | 1 | 3278808.375 | 1.35 | High |
| DAIIS | 5 | | 518.28205 | 518.2807 | -2.61 | 1 | 8543845 | 0.83 | Medium |
| DAMF | 4 | 1xOxidation [M3] | 499.18571 | 499.1846 | -2.22 | 1 | 5938820.5 | 0.84 | Medium |
| DAVDAVAV | 8 | | 759.38831 | 759.38672 | -2.1 | 1 | 3793671.75 | 0.96 | Medium |
| DAVDAVLG | 8 | | 759.38831 | 759.38672 | -2.1 | 1 | 3793671.75 | 0.96 | Medium |
| DDAPNRL | 7 | | 800.38971 | 400.69751 | -2.45 | 2 | 1224743.5 | 1.2 | Medium |
| DDISI | 5 | | 562.27188 | 562.27045 | -2.56 | 1 | 489225.5313 | 0.86 | Medium |
| DDLTDPAPA | 9 | | 914.41017 | 914.40802 | -2.35 | 1 | 954478.4375 | 0.73 | Medium |
| DDTPVTPAPTPA | 12 | | 1181.56846 | 1181.56531 | -2.67 | 1 | 257910.4375 | 0.75 | Medium |
| DDTVI | 5 | | 562.27188 | 562.27045 | -2.56 | 1 | 489225.5313 | 0.82 | Medium |
| DEGNLVA | 7 | | 717.34136 | 717.3396 | -2.45 | 1 | 401176.9688 | 1.02 | Medium |
| DEVI | 4 | | 475.23986 | 475.23871 | -2.41 | 1 | 7106328.5 | 0.76 | Medium |
| DFKGP | 5 | | 563.28239 | 563.28113 | -2.24 | 1 | 3105287.75 | 1.03 | Medium |
| DGDGGLV | 7 | | 632.2886 | 632.28735 | -1.97 | 1 | 2001603.25 | 0.74 | Medium |
| DGGML | 5 | | 492.21226 | 492.21063 | -3.31 | 1 | 1687721.75 | 0.76 | Medium |
| DGITLS | 6 | | 605.31408 | 605.31085 | -5.34 | 1 | 26197130 | 0.86 | Medium |
| DGITSI | 6 | | 605.31408 | 605.31085 | -5.34 | 1 | 26197130 | 0.86 | Medium |
| DGMLE | 5 | | 564.23339 | 564.23212 | -2.26 | 1 | 147385.5625 | 1.02 | Medium |
| DGPGI | 5 | | 458.22454 | 458.22339 | -2.51 | 1 | 592150.625 | 0.79 | Medium |
| DGSDGW | 6 | | 636.226 | 636.22406 | -3.04 | 1 | 11625267 | 1.06 | Medium |
| DGSVIL | 6 | | 603.33482 | 603.33307 | -2.9 | 1 | 1116935 | 0.7 | Medium |
| DGVVIH | 6 | | 639.34605 | 639.34412 | -3.03 | 1 | 830024.25 | 1.01 | Medium |
| DHARF | 5 | | 645.31033 | 323.15793 | -2.72 | 2 | 136800.5625 | 0.89 | Medium |
| DHKEAIIS | 8 | | 912.47852 | 912.47772 | -0.88 | 1 | 16862267.25 | 1.48 | High |
| DHISI | 5 | | 584.30385 | 584.30298 | -1.5 | 1 | 719384.375 | 0.71 | Medium |
| DKIEIN | 6 | | 731.3934 | 731.39185 | -2.12 | 1 | 6153296 | 0.88 | Medium |
| DKNSPI | 6 | | 673.35153 | 673.34955 | -2.94 | 1 | 2870663.75 | 0.97 | Medium |
| DIDIDVGGMVDFAR | 14 | | 1522.72062 | 508.24936 | 8.47 | 3 | 2831342.016 | 1.16 | Medium |
| DIEI | 4 | | 489.25551 | 489.25406 | -2.96 | 1 | 2683889.25 | 1.04 | Medium |
| DIGLE | 5 | | 546.27697 | 546.27423 | -5.01 | 1 | 3583164.25 | 0.76 | Medium |
| DILAE | 5 | | 560.29262 | 560.2912 | -2.53 | 1 | 425404.5 | 1 | Medium |
| DIIGGH | 6 | | 611.31475 | 611.3125 | -3.68 | 1 | 1397604.875 | 1.7 | High |
| DINSLTESL | 9 | | 991.49423 | 331.16968 | 0.25 | 3 | | 1.03 | Medium |
| DLPDRK | 6 | | 743.40463 | 372.20502 | -2.52 | 2 | 1803041 | 0.87 | Medium |
| DLPRDK | 6 | | 743.40463 | 372.20502 | -2.52 | 2 | 1803041 | 0.87 | Medium |
| DITI | 4 | | 461.26059 | 461.25949 | -2.38 | 1 | 78935728 | 0.91 | Medium |
| DIVPS | 5 | | 530.28205 | 530.28088 | -2.21 | 1 | 1436009.375 | 0.94 | Medium |
| DIVSP | 5 | | 530.28205 | 530.28088 | -2.21 | 1 | 1436009.375 | 0.94 | Medium |
| DNMI | 4 | | 492.21226 | 492.21063 | -3.31 | 1 | 1687721.75 | 0.76 | Medium |
| DPAGI | 5 | | 472.24019 | 472.23923 | -2.04 | 1 | 2332819.75 | 0.77 | Medium |
| DPGGI | 5 | | 458.22454 | 458.22339 | -2.51 | 1 | 592150.625 | 0.79 | Medium |
| DPIEL | 5 | | 586.30827 | 586.30688 | -2.36 | 1 | 6733985 | 0.89 | Medium |
| DPIPK | 5 | | 569.32934 | 569.32813 | -2.13 | 1 | 4732611.5 | 0.85 | Medium |
| DPQI | 4 | | 472.24019 | 472.23923 | -2.04 | 1 | 2332819.75 | 0.77 | Medium |
| DPTLAG | 6 | | 573.28787 | 573.28638 | -2.6 | 1 | 4079337.25 | 1.02 | Medium |
| DQPVTV | 6 | | 658.34063 | 658.33923 | -2.12 | 1 | 2739944 | 1.09 | Medium |
| DRFPSQW | 7 | | 935.43699 | 935.43213 | -5.2 | 1 | 8741917 | 0.94 | Medium |
| DRIM | 4 | | 534.27044 | 534.26892 | -2.85 | 1 | 1013516.125 | 1.1 | Medium |
| DRIPNPA | 7 | | 782.41553 | 391.71024 | -2.98 | 2 | 891926.25 | 1.17 | Medium |
| DRNLP | 5 | | 614.32565 | 614.32477 | -1.44 | 1 | 3540734.5 | 1.28 | High |
| DRNPL | 5 | | 614.32565 | 614.32477 | -1.44 | 1 | 3540734.5 | 1.28 | High |
| DRQAF | 5 | | 636.31 | 636.30865 | -2.11 | 1 | 2484203 | 1.29 | High |
| DTDIV | 5 | | 562.27188 | 562.27045 | -2.56 | 1 | 896799.0625 | 0.77 | Medium |
| DTYIE | 5 | | 640.28245 | 640.28125 | -1.87 | 1 | 986687.25 | 0.72 | Medium |
| DVAEQIR | 7 | | 830.43666 | 415.72058 | -3.34 | 2 | 1748187.5 | 0.85 | Medium |
| DVEDMPNLR | 9 | 1xOxidation [M5] | 1104.499 | 552.75171 | -2.59 | 2 | 1213684.75 | 1.25 | Medium |
| DVGDIA | 6 | | 589.28278 | 589.28156 | -2.08 | 1 | 6428334.5 | 0.99 | Medium |
| DVGGGFQ | 7 | | 679.30458 | 679.302 | -3.8 | 1 | 2899761.25 | 1.38 | High |
| DVLDPK | 6 | | 686.37193 | 686.36938 | -3.71 | 1 | 1905262.125 | 1.36 | High |
| DVMGIR | 6 | | 690.36032 | 345.68115 | -7.67 | 2 | 16382090.81 | 0.95 | Medium |
| DYKFK | 5 | | 700.36645 | 700.36578 | -0.95 | 1 | 1142515.125 | 1.41 | High |
| DYTPV | 5 | | 594.27697 | 594.27521 | -2.96 | 1 | 12521226 | 1.05 | Medium |
| DYTTM | 5 | | 630.24395 | 630.24231 | -2.61 | 1 | 8685706 | 0.95 | Medium |
| EAAIP | 5 | | 500.27149 | 500.27014 | -2.69 | 1 | 6027148 | 1.03 | Medium |
| EAAPI | 5 | | 500.27149 | 500.27014 | -2.69 | 1 | 6027148 | 1.03 | Medium |
| EALQGEIQ | 8 | | 887.44689 | 887.448 | 1.25 | 1 | 871451.8125 | 0.73 | Medium |
| EAVADITP | 8 | | 815.41452 | 408.2149 | 9.82 | 2 | 776661.25 | 0.81 | Medium |
| EDAIAQLQ | 8 | | 887.44689 | 887.448 | 1.25 | 1 | 871451.8125 | 0.73 | Medium |
| EDGTKF | 6 | | 696.3199 | 696.31799 | -2.73 | 1 | 785880.125 | 0.74 | Medium |
| EDLGIN | 6 | | 660.3199 | 660.31848 | -2.14 | 1 | 17306406 | 1.2 | High |
| EDPEFE | 6 | | 765.29374 | 765.29095 | -3.64 | 1 | 788647.25 | 1.48 | High |
| EDVI | 4 | | 475.23986 | 475.23871 | -2.41 | 1 | 7106328.5 | 0.75 | Medium |
| EENRPVLD | 8 | | 971.47925 | 486.24176 | -3.09 | 2 | 1021581.625 | 1.18 | Medium |
| EGANF | 5 | | 537.23035 | 537.22772 | -4.9 | 1 | 1004501.438 | 0.75 | Medium |
| EGDTLV | 6 | | 633.309 | 633.30695 | -3.24 | 1 | 1130173.375 | 0.82 | Medium |
| EGDTVL | 6 | | 633.309 | 633.30695 | -3.24 | 1 | 1130173.375 | 0.82 | Medium |
| EGFR | 4 | | 508.25142 | 508.25031 | -2.2 | 1 | 205533.9453 | 0.71 | Medium |
| EGGGAF | 6 | | 537.23035 | 537.22772 | -4.9 | 1 | 1004501.438 | 0.74 | Medium |

| | | | | | | | | | | |
|---|---|---|---|---|---|---|---|---|---|---|
| EGGIDEGM | 8 | 1xOxidation [M8] | 823.31382 | 823.31274 | -1.31 | 1 | 862977.125 | 0.93 | Medium |
| EGLQINPHIP | 10 | | 1117.60003 | 559.30725 | 6.43 | 2 | 1817487.625 | 0.92 | Medium |
| EGITV | 5 | | 518.28205 | 518.2807 | -2.61 | 1 | 8543845 | 0.83 | Medium |
| EGMF | 4 | 1xOxidation [M3] | 499.18571 | 499.1846 | -2.22 | 1 | 5938820.5 | 0.84 | Medium |
| EGQEIALQ | 8 | | 887.44689 | 887.448 | 1.25 | 1 | 871451.8125 | 0.73 | Medium |
| EHTGEYV | 7 | | 834.36282 | 417.68369 | -3.27 | 2 | 1723706.5 | 1.06 | Medium |
| EKTKIGY | 7 | | 838.46689 | 838.46606 | -0.99 | 1 | 4191760.25 | 1.56 | High |
| EIDI | 4 | | 489.25551 | 489.25452 | -2.02 | 1 | 2683889.25 | 0.98 | Medium |
| ELDYIM | 6 | | 783.35932 | 392.18454 | 3.17 | 2 | | 0.85 | Medium |
| EIEI | 4 | | 503.27116 | 503.26974 | -2.8 | 1 | 6904470.75 | 0.72 | Medium |
| EIEQQLQ | 7 | | 887.44689 | 887.448 | 1.25 | 1 | 871451.8125 | 0.73 | Medium |
| EIGY | 4 | | 481.22929 | 481.22784 | -3.01 | 1 | 235788192 | 0.96 | Medium |
| EIIAG | 5 | | 502.28714 | 502.2858 | -2.67 | 1 | 2888925.25 | 0.81 | Medium |
| EIIE | 4 | | 503.27116 | 503.26721 | -7.84 | 1 | 9909028 | 0.71 | Medium |
| EIIEAG | 6 | | 631.32973 | 631.32764 | -3.32 | 1 | 1348242.5 | 0.84 | Medium |
| ELLEGA | 6 | | 631.32973 | 631.32764 | -3.32 | 1 | 1348242.5 | 0.84 | Medium |
| EIIEQ | 5 | | 631.32973 | 631.32764 | -3.32 | 1 | 1348242.5 | 0.84 | Medium |
| ELIGA | 5 | | 502.28714 | 502.2858 | -2.67 | 1 | 2888925.25 | 0.81 | Medium |
| EIIQ | 4 | | 502.28714 | 502.2858 | -2.67 | 1 | 2888925.25 | 0.81 | Medium |
| EIMDSERL | 8 | 1xOxidation [M3] | 1008.46664 | 504.73422 | -5.42 | 2 | | 0.84 | Medium |
| ELQQELQ | 7 | | 887.44689 | 887.448 | 1.25 | 1 | 871451.8125 | 0.73 | Medium |
| ELTPAGLNLLDGR | 13 | | 1368.74815 | 684.87598 | -2.54 | 2 | 4034899 | 1.15 | Medium |
| EMPAE | 5 | | 576.23339 | 576.23175 | -2.84 | 1 | 1166789.25 | 0.92 | Medium |
| EMRPTGGGGGGAY | 13 | 1xOxidation [M2] | 1225.52661 | 1225.53394 | 5.98 | 1 | 1001313.5 | 0.82 | Medium |
| EMSIK | 5 | | 607.31197 | 607.31012 | -3.05 | 1 | 1641475.5 | 1.16 | Medium |
| ENDHAF | 6 | | 732.29474 | 732.29382 | -1.26 | 1 | 3305468.75 | 1.68 | High |
| ENLVTT | 6 | | 676.3512 | 676.34882 | -3.52 | 1 | 1391979.5 | 0.7 | Medium |
| ENTIP | 5 | | 573.28787 | 573.28613 | -3.03 | 1 | 4079337.25 | 0.85 | Medium |
| ENTPI | 5 | | 573.28787 | 573.28613 | -3.03 | 1 | 4079337.25 | 0.85 | Medium |
| EPQIE | 5 | | 615.29843 | 615.29736 | -1.74 | 1 | 168858.1875 | 0.77 | Medium |
| EQAIDAIQ | 8 | | 887.44689 | 887.448 | 1.25 | 1 | 871451.8125 | 0.73 | Medium |
| EQIEQIQ | 7 | | 887.44689 | 887.448 | 1.25 | 1 | 871451.8125 | 0.73 | Medium |
| EQPLE | 5 | | 615.29843 | 615.29736 | -1.74 | 1 | 168858.1875 | 0.77 | Medium |
| EQSDFLQ | 7 | | 866.38904 | 866.39178 | 3.17 | 1 | 817335.25 | 0.7 | Medium |
| ERPSFR | 6 | | 791.41586 | 396.21054 | -2.6 | 2 | 574582.375 | 1.37 | Medium |
| ERPVIE | 6 | | 742.40938 | 371.70786 | -1.27 | 2 | 2322758.75 | 1.28 | Medium |
| ERRIILSG | 8 | | 943.56834 | 315.19315 | -3.66 | 3 | 905925 | 1.36 | Medium |
| ERTIP | 5 | | 615.34605 | 615.34467 | -2.25 | 1 | 47456300 | 1.35 | High |
| ERTPL | 5 | | 615.34605 | 615.34467 | -2.25 | 1 | 47456300 | 1.41 | High |
| ERWGKPG | 7 | | 829.43151 | 415.21817 | -2.95 | 2 | 3043229.531 | 1.13 | Medium |
| ESDIV | 5 | | 562.27188 | 562.27045 | -2.56 | 1 | 896799.0625 | 0.8 | Medium |
| ESDVL | 5 | | 562.27188 | 562.27045 | -2.56 | 1 | 896799.0625 | 0.8 | Medium |
| ESEEYPK | 7 | | 881.3887 | 881.38538 | -3.78 | 1 | 995118.3125 | 0.75 | Medium |
| ESNKRLDSV | 9 | | 1047.54291 | 524.27374 | -2.58 | 2 | 984068.375 | 1.07 | Medium |
| ESRLNHHLA | 9 | | 1076.55957 | 538.78076 | -4.94 | 2 | 250163.9219 | 1.23 | Medium |
| ESYIE | 5 | | 640.28245 | 640.28125 | -1.87 | 1 | 986687.25 | 0.71 | Medium |
| ETPLTE | 6 | | 689.33521 | 689.33386 | -1.96 | 1 | 1240376.25 | 0.94 | Medium |
| ETPTEI | 6 | | 689.33521 | 689.33386 | -1.96 | 1 | 1240376.25 | 0.96 | Medium |
| ETTFPK | 6 | | 722.37193 | 722.36981 | -2.93 | 1 | 1999459.375 | 1.33 | High |
| EVDLTQ | 6 | | 704.34611 | 704.34381 | -3.27 | 1 | 1060646.625 | 1.02 | Medium |
| EVEI | 4 | | 489.25551 | 489.25406 | -2.96 | 1 | 2683889.25 | 1.04 | Medium |
| EVFAG | 5 | | 522.25584 | 522.25464 | -2.3 | 1 | 2093721.75 | 1.03 | Medium |
| EVFGA | 5 | | 522.25584 | 522.25464 | -2.3 | 1 | 2093721.75 | 1.02 | Medium |
| EVFQ | 4 | | 522.25584 | 522.25464 | -2.3 | 1 | 2093721.75 | 1.03 | Medium |
| EVGIE | 5 | | 546.27697 | 546.27423 | -5.01 | 1 | 3583164.25 | 0.76 | Medium |
| EVGTVV | 6 | | 603.33482 | 603.33307 | -2.9 | 1 | 1116935 | 0.7 | Medium |
| EVIAE | 5 | | 560.29262 | 560.2912 | -2.53 | 1 | 425404.5 | 1 | Medium |
| EVIPR | 5 | | 613.36679 | 307.1864 | -2.06 | 2 | 10368106.25 | 1.17 | Medium |
| EVLQQAQ | 7 | | 815.42576 | 408.2149 | -3.96 | 2 | 776661.25 | 0.81 | Medium |
| EVVSP | 5 | | 530.28205 | 530.28088 | -2.21 | 1 | 1436009.375 | 0.93 | Medium |
| EVVTT | 5 | | 548.29262 | 548.29199 | -1.14 | 1 | | 0.84 | Medium |
| EYDQAIQ | 7 | | 866.38904 | 866.39178 | 3.17 | 1 | 817335.25 | 0.7 | Medium |
| EYISPE | 6 | | 737.33521 | 737.33356 | -2.24 | 1 | 1589787.75 | 0.95 | Medium |
| EYQIR | 5 | | 708.36752 | 708.36572 | -2.53 | 1 | 17573248 | 1.31 | High |
| FDEGSTIN | 8 | | 882.38395 | 882.38171 | -2.54 | 1 | 1057948.5 | 1.23 | High |
| FDGVE | 5 | | 566.24567 | 566.24445 | -2.16 | 1 | 2198855 | 0.81 | Medium |
| FDNIR | 5 | | 664.3413 | 332.67358 | -2.12 | 2 | 3298101.25 | 0.94 | Medium |
| FDRVC | 5 | | 639.29191 | 320.14865 | -2.94 | 2 | 3839002.75 | 1.04 | Medium |
| FDYR | 4 | | 600.27764 | 300.64136 | -3.66 | 2 | 4187493.5 | 0.83 | Medium |
| FGDPE | 5 | | 564.23002 | 564.23047 | 0.8 | 1 | 1845533.625 | 1.23 | High |
| FGPGA | 5 | | 448.21906 | 448.21817 | -1.99 | 1 | 22379732 | 0.88 | Medium |
| FGPQ | 4 | | 448.21906 | 448.21817 | -1.99 | 1 | 22379732 | 0.88 | Medium |
| FGQPAVD | 7 | | 733.35153 | 733.34998 | -2.12 | 1 | 1385996 | 1.13 | Medium |
| FKGYRPQ | 7 | | 895.47846 | 895.47644 | -2.26 | 1 | 3248115.063 | 1.37 | High |
| FIAG | 4 | | 407.2289 | 407.22772 | -2.88 | 1 | 25617920 | 0.79 | Medium |
| FIGA | 4 | | 407.2289 | 407.22772 | -2.88 | 1 | 25617920 | 0.79 | Medium |
| FINPS | 5 | | 577.29804 | 577.29272 | -9.2 | 1 | 1534005.75 | 0.74 | Medium |
| FLNSP | 5 | | 577.29804 | 577.29272 | -9.2 | 1 | 1534005.75 | 0.74 | Medium |
| FITGG | 5 | | 494.26092 | 494.2597 | -2.47 | 1 | 4133417.5 | 0.98 | Medium |
| FITN | 4 | | 494.26092 | 494.2597 | -2.47 | 1 | 4133417.5 | 0.98 | Medium |
| FITR | 4 | | 536.31911 | 268.66235 | -3.13 | 2 | 1358602.125 | 0.96 | Medium |
| FMPR | 4 | | 550.28061 | 550.27765 | -5.39 | 1 | 1002354.75 | 0.8 | Medium |
| FNGKID | 6 | | 693.35662 | 693.35535 | -1.83 | 1 | 2242486 | 1.3 | High |

| | | | | | | | | | | |
|---|---|---|---|---|---|---|---|---|---|---|
| FNTNPE | 6 | | 721.31515 | 721.31274 | -3.33 | 1 | 3219601.5 | 0.78 | Medium |
| FQKRPDVVSPGGN | 13 | | 1400.72809 | 467.57919 | -3.61 | 3 | 2429558.938 | 2.11 | Medium |
| FRNIGSS | 7 | | 780.39988 | 390.70236 | -3.11 | 2 | 1215340.75 | 1.53 | Medium |
| FRYPI | 5 | | 695.38752 | 695.38184 | -8.18 | 1 | 1233173.25 | 0.76 | Medium |
| FSIEPK | 6 | | 720.39267 | 720.39099 | -2.33 | 1 | 8677017 | 1.28 | High |
| FSNSQP | 6 | | 679.30458 | 679.30292 | -2.45 | 1 | | 0.9 | Medium |
| FSNY | 4 | | 530.22454 | 530.22296 | -2.98 | 1 | 1484211.875 | 0.73 | Medium |
| FSVAG | 5 | | 480.24527 | 480.24368 | -3.31 | 1 | | 0.8 | Medium |
| FSVGA | 5 | | 480.24527 | 480.24368 | -3.31 | 1 | | 0.8 | Medium |
| FSVGG | 5 | | 466.22962 | 466.22797 | -3.56 | 1 | 2565986.75 | 0.86 | Medium |
| FSVN | 4 | | 466.22962 | 466.22797 | -3.56 | 1 | 2565986.75 | 0.86 | Medium |
| FSVQ | 4 | | 480.24527 | 480.24368 | -3.31 | 1 | | 0.8 | Medium |
| FTDPA | 5 | | 550.25075 | 550.24927 | -2.7 | 1 | 2658656.5 | 0.76 | Medium |
| FVGIMLERIR | 10 | 1xOxidation [M5] | 1249.70854 | 417.24405 | 7.25 | 3 | 223979.4688 | 1.14 | Medium |
| FYDR | 4 | | 600.27764 | 300.64136 | -3.66 | 2 | 4187493.5 | 0.84 | Medium |
| FYDRA | 5 | | 671.31475 | 671.31323 | -2.26 | 1 | 29404902 | 1.06 | Medium |
| FYPN | 4 | | 540.24527 | 540.24353 | -3.23 | 1 | 18538314 | 0.88 | Medium |
| GADGAF | 6 | | 537.23035 | 537.22772 | -4.9 | 1 | 1004501.438 | 0.74 | Medium |
| GADILE | 6 | | 617.31408 | 617.31274 | -2.17 | 1 | 796395.125 | 0.93 | Medium |
| GAEGF | 5 | | 480.20889 | 480.20782 | -2.22 | 1 | 12471353 | 0.74 | Medium |
| GAENF | 5 | | 537.23035 | 537.22772 | -4.9 | 1 | 1004501.438 | 0.75 | Medium |
| GAGGVIPPN | 9 | | 781.42028 | 781.41882 | -1.86 | 1 | 1091475.5 | 1.36 | High |
| GAIDL | 5 | | 488.27149 | 488.27075 | -1.51 | 1 | 1568978.75 | 0.7 | Medium |
| GAIIE | 5 | | 502.28714 | 502.28705 | -0.18 | 1 | 1159842.25 | 0.99 | Medium |
| GANAF | 5 | | 479.22487 | 479.22382 | -2.21 | 1 | 10988858 | 1.03 | Medium |
| GASVI | 5 | | 446.26092 | 446.26132 | 0.89 | 1 | 20861782 | 0.83 | Medium |
| GAVVPS | 6 | | 529.29804 | 529.29675 | -2.43 | 1 | 3637882.25 | 0.89 | Medium |
| GDALVTT | 7 | | 676.3512 | 676.34882 | -3.52 | 1 | 1391979.5 | 0.7 | Medium |
| GDGMI | 5 | | 492.21226 | 492.21063 | -3.31 | 1 | 1687721.75 | 0.76 | Medium |
| GDHPAL | 6 | | 609.2991 | 609.29626 | -4.66 | 1 | 1325655.25 | 1.09 | Medium |
| GDLTVT | 6 | | 605.31408 | 605.31085 | -5.34 | 1 | 26197130 | 0.86 | Medium |
| GDPGI | 5 | | 458.22454 | 458.22339 | -2.51 | 1 | 592150.625 | 0.79 | Medium |
| GDRPVRIG | 8 | | 869.49518 | 290.50204 | -4.13 | 3 | 1115629 | 1.64 | Medium |
| GDSDGW | 6 | | 636.226 | 636.22406 | -3.04 | 1 | 11625267 | 1.06 | Medium |
| GDSDRF | 6 | | 696.29474 | 696.29211 | -3.78 | 1 | 1372172.938 | 1.01 | Medium |
| GDVTPIEE | 8 | | 859.40435 | 859.40277 | -1.84 | 1 | 22909448 | 1.52 | High |
| GEDKVQID | 8 | | 903.4418 | 452.22488 | 0.76 | 2 | 932915.3125 | 1.02 | Medium |
| GEDLQLR | 7 | | 830.43666 | 415.72058 | -3.34 | 2 | 1748187.5 | 0.85 | Medium |
| GEDTVL | 6 | | 633.309 | 633.30695 | -3.24 | 1 | 1130173.375 | 0.82 | Medium |
| GEDTVPYR | 8 | | 936.44214 | 468.72379 | -1.97 | 2 | 2433171.75 | 2.2 | High |
| GEFAPT | 6 | | 621.28787 | 621.28705 | -1.32 | 1 | 1020595.438 | 0.73 | Medium |
| GEFR | 4 | | 508.25142 | 508.25031 | -2.2 | 1 | 140705.6719 | 0.71 | Medium |
| GEIIK | 5 | | 559.34499 | 559.34344 | -2.76 | 1 | 3278808.375 | 1.36 | High |
| GEIIS | 5 | | 518.28205 | 518.2807 | -2.61 | 1 | 8543845 | 0.83 | Medium |
| GEITV | 5 | | 518.28205 | 518.2807 | -2.61 | 1 | 8543845 | 0.83 | Medium |
| GEMF | 4 | 1xOxidation [M3] | 499.18571 | 499.1846 | -2.22 | 1 | 5938820.5 | 0.84 | Medium |
| GFDPE | 5 | | 564.23002 | 564.23047 | 0.8 | 1 | | 1.19 | Medium |
| GFEAP | 5 | | 520.24019 | 520.23871 | -2.85 | 1 | 38232408 | 0.71 | Medium |
| GFEPA | 5 | | 520.24019 | 520.23871 | -2.85 | 1 | 38232408 | 0.71 | Medium |
| GGDVIREA | 8 | | 816.42101 | 816.41968 | -1.63 | 1 | 7481874 | 1.63 | High |
| GGKPF | 5 | | 505.27691 | 253.14163 | -1.82 | 2 | 5636452.5 | 0.9 | Medium |
| GGIGHY | 6 | | 603.28854 | 603.28754 | -1.66 | 1 | 653699.375 | 0.8 | Medium |
| GGILR | 5 | | 515.33001 | 515.32867 | -2.59 | 1 | 4643888.5 | 1.19 | Medium |
| GGSEEPQPTPPNVD | 14 | | 1423.63358 | 1423.63318 | -0.28 | 1 | 4214892.625 | 1.02 | Medium |
| GGSGGN | 6 | | 448.17865 | 448.181 | 5.24 | 1 | 657374.125 | 0.99 | Medium |
| GGTGLY | 6 | | 567.2773 | 567.27582 | -2.62 | 1 | 913628.625 | 0.79 | Medium |
| GGTVI | 5 | | 446.26092 | 446.26132 | 0.89 | 1 | 20861782 | 0.83 | Medium |
| GGYPLR | 6 | | 662.36204 | 331.68384 | -2.47 | 2 | 14522127 | 1.24 | Medium |
| GHGLGI | 6 | | 553.30927 | 553.30817 | -2 | 1 | 949262.1875 | 1.04 | Medium |
| GHHPNPLPH | 9 | | 1005.50132 | 335.83786 | -2.28 | 3 | 547548.8828 | 1.39 | Medium |
| GHKGIY | 6 | | 674.36204 | 337.68387 | -2.34 | 2 | 403446.75 | 1.39 | Medium |
| GHNYY | 5 | | 653.2678 | 653.26404 | -5.76 | 1 | 3267150.563 | 0.71 | Medium |
| GKIIPQ | 6 | | 655.41374 | 655.41223 | -2.3 | 1 | 2329077 | 1.46 | High |
| GKPF | 4 | | 448.25545 | 448.25433 | -2.48 | 1 | 1158918.625 | 1.01 | Medium |
| GKSPLYR | 7 | | 820.46756 | 410.73642 | -2.44 | 2 | 11198786 | 1.93 | High |
| GIDEIN | 6 | | 660.3199 | 660.31848 | -2.14 | 1 | 17306406 | 1.24 | High |
| GLDEVAG | 7 | | 660.3199 | 660.31848 | -2.14 | 1 | | 1.19 | Medium |
| GIDGIKNE | 8 | | 845.43632 | 423.22061 | -2.81 | 2 | 1825956.625 | 0.89 | Medium |
| GLDTPAS | 7 | | 660.3199 | 660.31824 | -2.51 | 1 | 17306406 | 1.35 | High |
| GIFSDR | 6 | | 694.35187 | 347.67877 | -2.3 | 2 | 1121682.75 | 0.81 | Medium |
| GLGDLAA | 7 | | 616.33007 | 616.32733 | -4.44 | 1 | 8176471 | 0.84 | Medium |
| GIGLAPR | 7 | | 683.41989 | 342.21271 | -2.56 | 2 | 479742.125 | 0.92 | Medium |
| GLGVSP | 6 | | 529.29804 | 529.29633 | -3.24 | 1 | 3637882.25 | 1.05 | Medium |
| GLGYRLNP | 8 | | 889.48903 | 445.2478 | -0.79 | 2 | | 0.87 | Medium |
| GIKIQ | 5 | | 558.36097 | 558.35968 | -2.32 | 1 | 1115697 | 1.41 | High |
| GIID | 4 | | 417.23438 | 417.23325 | -2.71 | 1 | 29495884 | 0.88 | Medium |
| GIIK | 4 | | 430.3024 | 430.30127 | -2.62 | 1 | 1368010.625 | 0.84 | Medium |
| GIIT | 4 | | 403.25511 | 403.25415 | -2.38 | 1 | 964773.5 | 0.8 | Medium |
| GILVM | 5 | 1xOxidation [M5] | 548.31125 | 548.31085 | -0.72 | 1 | 1267634.875 | 1.11 | Medium |
| GIREPVAG | 8 | | 798.44683 | 798.44482 | -2.51 | 1 | 48330912.5 | 1.41 | High |
| GLWEFPGGK | 9 | | 990.50434 | 495.75189 | -7.91 | 2 | 1287403 | 0.96 | Medium |
| GIYEGR | 6 | | 694.35187 | 347.67877 | -2.3 | 2 | 1121682.75 | 0.81 | Medium |
| GMIT | 4 | | 421.21153 | 421.2103 | -2.93 | 1 | 3729498.75 | 0.74 | Medium |

| | | | | | | | | | |
|---|---|---|---|---|---|---|---|---|---|
| GMTDVSVEKQ | 10 | | 1093.5194 | 547.26276 | -1.06 | 2 | 1017546 | 1.31 | Medium |
| GNDILSKDILFY | 12 | | 1397.73111 | 1397.72253 | -6.13 | 1 | 1808993.625 | 0.76 | Medium |
| GNGGLLS | 7 | | 617.32532 | 617.32452 | -1.28 | 1 | 1241508.25 | 0.88 | Medium |
| GNNIIS | 6 | | 617.32532 | 617.32452 | -1.28 | 1 | 1241508.25 | 0.89 | Medium |
| GNSGN | 5 | | 448.17865 | 448.181 | 5.24 | 1 | 657374.125 | 0.99 | Medium |
| GNSRPILR | 8 | | 912.53737 | 456.77133 | -2.18 | 2 | 3486132.813 | 1.94 | High |
| GPDGL | 5 | | 458.22454 | 458.22339 | -2.51 | 1 | 592150.625 | 0.79 | Medium |
| GPFAR | 5 | | 547.29871 | 547.297 | -3.12 | 1 | 4702021.25 | 1.21 | High |
| GPKGF | 5 | | 505.27691 | 505.27567 | -2.46 | 1 | 454210.8438 | 1.3 | High |
| GPLATVG | 7 | | 614.3508 | 614.34894 | -3.03 | 1 | 497514912 | 0.83 | Medium |
| GQDFR | 5 | | 622.29435 | 311.64996 | -2.73 | 2 | 272638.2813 | 0.87 | Medium |
| GQDLP | 5 | | 529.26165 | 529.2608 | -1.61 | 1 | 3142693.75 | 1.09 | Medium |
| GQDPL | 5 | | 529.26165 | 529.2608 | -1.61 | 1 | 3142693.75 | 1.09 | Medium |
| GQGWE | 5 | | 576.24125 | 576.24023 | -1.77 | 1 | 1150539.875 | 0.81 | Medium |
| GQIYE | 5 | | 609.28787 | 609.28705 | -1.34 | 1 | | 1.16 | Medium |
| GQPVEL | 6 | | 642.34572 | 642.34265 | -4.77 | 1 | 2527219 | 0.97 | Medium |
| GRDGF | 5 | | 551.25724 | 551.25562 | -2.94 | 1 | 1816801.25 | 1.15 | Medium |
| GREEPGGNL | 9 | | 928.44828 | 464.72595 | -3.94 | 2 | 4433430 | 1.6 | Medium |
| GREGDLVE | 8 | | 874.42649 | 437.71573 | -2.64 | 2 | 1735490.375 | 1.32 | Medium |
| GRFR | 4 | | 535.30994 | 268.15802 | -2.2 | 2 | 961049 | 0.81 | Medium |
| GRPPGVIGQ | 9 | | 880.49993 | 440.75208 | -3.47 | 2 | 1012369.375 | 1.18 | Medium |
| GRYID | 5 | | 623.31475 | 312.16034 | -2.16 | 2 | 1536394.188 | 0.91 | Medium |
| GRYIISISA | 9 | | 979.55711 | 490.27859 | -7.34 | 2 | 726929.375 | 0.97 | Medium |
| GSAVI | 5 | | 446.26092 | 446.26132 | 0.89 | 1 | 20861782 | 0.83 | Medium |
| GSIQPVAPS | 9 | | 855.45706 | 855.45544 | -1.89 | 1 | 6638699.5 | 1.09 | Medium |
| GSIY | 4 | | 439.21873 | 439.21759 | -2.59 | 1 | 3598803 | 0.77 | Medium |
| GSVLLLAALG | 10 | | 913.57169 | 913.57159 | -0.11 | 1 | | 0.96 | Medium |
| GTAPGSL | 7 | | 602.31442 | 602.31305 | -2.27 | 1 | 2002563.625 | 0.99 | Medium |
| GTGIY | 5 | | 510.25584 | 510.25339 | -4.81 | 1 | 1668234.125 | 0.87 | Medium |
| GTGVI | 5 | | 446.26092 | 446.26132 | 0.89 | 1 | 20861782 | 0.83 | Medium |
| GVAVDPDGQ | 9 | | 857.39994 | 429.20782 | 9.84 | 2 | | 0.99 | Medium |
| GVAVSP | 6 | | 529.29804 | 529.29675 | -2.43 | 1 | 3637882.25 | 0.89 | Medium |
| GVDIE | 5 | | 532.26132 | 532.26056 | -1.43 | 1 | 3222326.75 | 0.79 | Medium |
| GVDLPRDK | 8 | | 899.49451 | 450.24991 | -2.19 | 2 | 2914482.75 | 1.31 | Medium |
| GVFELIR | 7 | | 833.48796 | 417.24405 | -8.57 | 2 | | 0.96 | Medium |
| GVGDIE | 6 | | 589.28278 | 589.28125 | -2.6 | 1 | 1123788.625 | 1.37 | High |
| GVKPI | 5 | | 513.33951 | 513.33844 | -2.08 | 1 | 784074.8125 | 0.83 | Medium |
| GVIAEPS | 7 | | 672.35628 | 672.35425 | -3.02 | 1 | 775776.1875 | 1 | Medium |
| GVIAEPSTAEPV | 12 | | 1169.60484 | 585.31024 | 7.15 | 2 | 20175128 | 0.97 | Medium |
| GVIGV | 5 | | 444.28166 | 444.28049 | -2.64 | 1 | 109830880 | 0.78 | Medium |
| GVIVG | 5 | | 444.28166 | 444.28049 | -2.64 | 1 | 109830880 | 0.78 | Medium |
| GVPAGL | 6 | | 513.30312 | 513.30194 | -2.3 | 1 | 1998379.75 | 1.03 | Medium |
| GVPIE | 5 | | 514.28714 | 514.28571 | -2.79 | 1 | 16506790 | 1.16 | Medium |
| GVPIGA | 6 | | 513.30312 | 513.30176 | -2.66 | 1 | 1998379.75 | 0.8 | Medium |
| GVPIGAT | 7 | | 614.3508 | 614.34937 | -2.34 | 1 | 497514912 | 1.02 | Medium |
| GVPQI | 5 | | 513.30312 | 513.30176 | -2.66 | 1 | 1998379.75 | 0.8 | Medium |
| GVRPVVG | 7 | | 683.41989 | 342.21307 | -1.48 | 2 | 926471.75 | 0.93 | Medium |
| GVSVPT | 6 | | 559.3086 | 559.30688 | -3.07 | 1 | 3308384.25 | 0.97 | Medium |
| GVTVLLGR | 8 | | 814.51451 | 272.1767 | 1.26 | 3 | 2877815.5 | 1.18 | Medium |
| GVVM | 4 | | 405.21662 | 405.21552 | -2.72 | 1 | 6955801 | 1.08 | Medium |
| GVVM | 4 | 1xOxidation [M4] | 421.21153 | 421.2106 | -2.21 | 1 | 133303.0781 | 0.82 | Medium |
| GYDADID | 7 | | 768.30464 | 768.30206 | -3.35 | 1 | 2504925.25 | 1.13 | Medium |
| GYIAF | 5 | | 570.29223 | 570.29425 | 3.55 | 1 | 8964888 | 0.7 | Medium |
| GYLIAM | 6 | | 667.34836 | 667.34967 | 1.96 | 1 | 1750997.75 | 0.74 | Medium |
| GYPKPPSEE | 9 | | 1003.4731 | 502.237 | -6.36 | 2 | | 0.81 | Medium |
| HDARF | 5 | | 645.31033 | 323.15793 | -2.72 | 2 | 136800.5625 | 0.89 | Medium |
| HDDDYIER | 8 | | 1062.44868 | 531.72662 | -2.55 | 2 | 4076503.75 | 2.64 | High |
| HDIIS | 5 | | 584.30385 | 584.30298 | -1.5 | 1 | 719384.375 | 0.72 | Medium |
| HDISL | 5 | | 584.30385 | 584.30298 | -1.5 | 1 | 719384.375 | 0.72 | Medium |
| HDITV | 5 | | 584.30385 | 584.30298 | -1.5 | 1 | 719384.375 | 0.71 | Medium |
| HDIY | 4 | | 547.25109 | 547.24908 | -3.66 | 1 | 2890270.25 | 0.84 | Medium |
| HEGEEPIYGK | 10 | | 1158.54258 | 579.77374 | -2.05 | 2 | 1322160.375 | 2.16 | High |
| HFGAY | 5 | | 594.26707 | 594.2655 | -2.64 | 1 | 2632376.438 | 0.74 | Medium |
| HFGPE | 5 | | 586.26199 | 586.26093 | -1.81 | 1 | 1000076.188 | 0.83 | Medium |
| HFVPEKPM | 8 | 1xOxidation [M8] | 1000.49206 | 500.74826 | -2.82 | 2 | 1434750.25 | 1.61 | Medium |
| HGGIVA | 6 | | 553.30927 | 553.30817 | -2 | 1 | 949262.1875 | 1.02 | Medium |
| HGGW | 4 | | 456.19899 | 456.19809 | -1.98 | 1 | 329342.8633 | 0.75 | Medium |
| HKGPFTGE | 8 | | 872.42609 | 436.71552 | -2.68 | 2 | 4103382 | 0.94 | Medium |
| HIDGIR | 6 | | 710.3944 | 355.69995 | -2.5 | 2 | 894190.0625 | 1.71 | Medium |
| HIEPVPG | 7 | | 748.39882 | 748.39606 | -3.69 | 1 | 1913483.625 | 0.98 | Medium |
| HIEPVPGEDN | 10 | | 1106.51128 | 553.75806 | -2.21 | 2 | 2852338.25 | 1.85 | Medium |
| HIIATMA | 7 | 1xOxidation [M6] | 772.40219 | 386.70755 | 7.3 | 2 | 2733587.75 | 1.19 | Medium |
| HLSVMLG | 7 | 1xOxidation [M5] | 772.40219 | 386.70755 | 7.3 | 2 | 2733587.75 | 1.18 | Medium |
| HITNGNFAQS | 10 | | 1088.51195 | 544.75745 | -3.98 | 2 | 2289996 | 1.62 | Medium |
| HMLAP | 5 | 1xOxidation [M2] | 584.28609 | 584.28448 | -2.75 | 1 | 3307319 | 0.85 | Medium |
| HMPAL | 5 | 1xOxidation [M2] | 584.28609 | 584.28448 | -2.75 | 1 | 3307319 | 0.85 | Medium |
| HNVPT | 5 | | 567.28854 | 567.28497 | -6.28 | 1 | | 0.79 | Medium |
| HNVTP | 5 | | 567.28854 | 567.28497 | -6.28 | 1 | | 0.8 | Medium |
| HPAP | 4 | | 421.21939 | 421.21826 | -2.69 | 1 | 31926316 | 0.75 | Medium |
| HQGIY | 5 | | 617.30419 | 617.30005 | -6.7 | 1 | 8131936.5 | 0.84 | Medium |
| HQITVLNSPG | 10 | | 1065.56873 | 533.28687 | -2.14 | 2 | 156675556 | 1.2 | Medium |
| HQYIA | 5 | | 631.31984 | 631.31659 | -5.14 | 1 | 1301004.75 | 0.93 | Medium |
| HSIGV | 5 | | 512.28272 | 512.28156 | -2.28 | 1 | 879027.3125 | 0.84 | Medium |

| | | | | | | | | | |
|---|---|---|---|---|---|---|---|---|---|
| HSNKPI | 6 | | 695.3835 | 695.38184 | -2.39 | 1 | 1233173.25 | 0.78 | Medium |
| HVFE | 4 | | 531.25617 | 531.255 | -2.2 | 1 | 1563680 | 0.84 | Medium |
| HVVV | 4 | | 453.28199 | 453.28085 | -2.52 | 1 | 172224.8906 | 0.7 | Medium |
| HYNQIR | 6 | | 830.42676 | 415.72058 | 8.58 | 2 | 1748187.5 | 0.85 | Medium |
| KAAEIR | 6 | | 687.4148 | 344.20999 | -3.05 | 2 | 1584398.25 | 0.88 | Medium |
| KAEFER | 6 | | 779.40463 | 390.20233 | -9.29 | 2 | 329134.5938 | 0.86 | Medium |
| KAIY | 4 | | 494.29731 | 494.29614 | -2.36 | 1 | 329168.2813 | 1.01 | Medium |
| KAVI | 4 | | 430.3024 | 430.30115 | -2.9 | 1 | 68206848.1 | 1.11 | Medium |
| KCEQPR | 6 | | 760.37703 | 380.6951 | 7.74 | 2 | 259371.3594 | 1.17 | Medium |
| KDDAVRSQDFDRA | 13 | | 1522.72446 | 508.24936 | 5.95 | 3 | 230996.7656 | 1.15 | Medium |
| KDELLN | 6 | | 731.3934 | 731.39185 | -2.12 | 1 | 6153296 | 0.9 | Medium |
| KDKPAEIVDPK | 11 | | 1239.69433 | 413.90219 | -1.86 | 3 | 960090.8125 | 1.07 | Medium |
| KDNTTIV | 7 | | 790.43051 | 395.71777 | -2.83 | 2 | 17862031 | 1.53 | Medium |
| KDPSNFDPR | 9 | | 1075.5167 | 538.2608 | -2.2 | 2 | 1406033.625 | 1.58 | Medium |
| KDRLM | 5 | | 662.36541 | 331.68542 | -2.77 | 2 | 432947.3672 | 1.15 | Medium |
| KDSTTIV | 7 | | 763.41961 | 763.41785 | -2.31 | 1 | 1533512.875 | 1.32 | High |
| KDTSLSL | 7 | | 763.41961 | 382.2131 | -0.89 | 2 | 1479363.5 | 0.86 | Medium |
| KDYIH | 5 | | 675.34605 | 675.34344 | -3.86 | 1 | | 0.78 | Medium |
| KDYTTM | 6 | | 758.33892 | 758.33716 | -2.32 | 1 | 9686441.25 | 1.74 | High |
| KDYTTM | 6 | 1xOxidation [M6] | 774.33383 | 774.33221 | -2.09 | 1 | 2305348.813 | 1.72 | High |
| KEAIH | 5 | | 597.33549 | 597.33362 | -3.13 | 1 | | 0.94 | Medium |
| KEFAER | 6 | | 779.40463 | 390.20233 | -9.29 | 2 | 329134.5938 | 0.86 | Medium |
| KEIKPY | 6 | | 777.45052 | 389.22903 | 0.35 | 2 | 1360428.5 | 0.87 | Medium |
| KELLEAH | 7 | | 839.46214 | 420.23392 | -1.89 | 2 | 4094771.75 | 1.62 | Medium |
| KEQHEDYKFK | 10 | | 1351.66409 | 676.33362 | -3.06 | 2 | 956432.2656 | 2.38 | High |
| KFAP | 4 | | 462.2711 | 462.27023 | -1.87 | 1 | 4208192.5 | 1.09 | Medium |
| KFIPGKDE | 8 | | 933.50401 | 467.25452 | -2.41 | 2 | 1023520.125 | 1.76 | Medium |
| KFPA | 4 | | 462.2711 | 462.27023 | -1.87 | 1 | 4208192.5 | 1.09 | Medium |
| KFPDP | 5 | | 603.31369 | 603.31567 | 3.29 | 1 | | 0.8 | Medium |
| KFPPD | 5 | | 603.31369 | 603.31567 | 3.29 | 1 | | 0.8 | Medium |
| KFSPE | 5 | | 607.3086 | 304.15704 | -2.95 | 2 | 2665487 | 0.96 | Medium |
| KGAIPY | 6 | | 648.37154 | 324.68848 | -2.87 | 2 | 2114230.5 | 1.05 | Medium |
| KGFP | 4 | | 448.25545 | 448.25439 | -2.34 | 1 | 2166931.734 | 0.82 | Medium |
| KGGIM | 5 | | 505.28028 | 505.27676 | -6.96 | 1 | 4553646 | 0.77 | Medium |
| KGGIY | 5 | | 537.30312 | 537.30267 | -0.84 | 1 | 1078988.625 | 1.15 | Medium |
| KGGML | 5 | | 505.28028 | 505.27676 | -6.96 | 1 | 4553646 | 0.77 | Medium |
| KGILPR | 6 | | 683.45627 | 342.23093 | -2.48 | 2 | 404505.0938 | 1.36 | Medium |
| KGIM | 4 | | 448.25882 | 448.25439 | -9.86 | 1 | 1957196.406 | 0.82 | Medium |
| KGIY | 4 | | 480.28166 | 480.28049 | -2.44 | 1 | 1801631.75 | 1.16 | Medium |
| KGMI | 4 | | 448.25882 | 448.25439 | -9.86 | 1 | 1957196.406 | 0.82 | Medium |
| KGMI | 4 | 1xOxidation [M3] | 464.25373 | 464.25247 | -2.71 | 1 | 212152.4063 | 0.74 | Medium |
| KGNAVP | 6 | | 585.33549 | 585.33508 | -0.69 | 1 | | 0.71 | Medium |
| KGNTASKMAAFLEELR | 16 | | 1765.92653 | 589.3183 | 7.82 | 3 | 2327663 | 1.03 | Medium |
| KGPF | 4 | | 448.25545 | 448.25433 | -2.48 | 1 | 3325850.359 | 1.06 | Medium |
| KGPGF | 5 | | 505.27691 | 505.27676 | -0.29 | 1 | 4553646 | 0.77 | Medium |
| KGPKGF | 6 | | 633.37187 | 633.37079 | -1.71 | 1 | 2209662.826 | 1.22 | High |
| KGSGFP | 6 | | 592.30894 | 592.31024 | 2.2 | 1 | 2577425.438 | 1.11 | Medium |
| KGSGLM | 6 | | 592.31231 | 592.31024 | -3.49 | 1 | 2577425.438 | 1.11 | Medium |
| KGSGMI | 6 | | 592.31231 | 592.31024 | -3.49 | 1 | 2577425.438 | 1.11 | Medium |
| KGTCI | 5 | | 521.27519 | 521.27338 | -3.49 | 1 | 536183.375 | 0.77 | Medium |
| KGVI | 4 | | 416.28675 | 416.28571 | -2.5 | 1 | 129897653.3 | 1.15 | Medium |
| KGVPL | 5 | | 513.33951 | 513.33844 | -2.08 | 1 | 784074.8125 | 0.8 | Medium |
| KHAPAL | 6 | | 636.38277 | 318.69406 | -3.03 | 2 | 6071827.5 | 1.1 | Medium |
| KHHDLR | 6 | | 805.44275 | 403.229 | 9.91 | 2 | 10698027 | 0.88 | Medium |
| KHPI | 4 | | 494.30854 | 494.30838 | -0.33 | 1 | 760101.0625 | 0.85 | Medium |
| KLCRIGR | 7 | | 845.5138 | 423.25934 | -2.84 | 2 | | 0.93 | Medium |
| KIDEV | 5 | | 603.33482 | 603.33331 | -2.49 | 1 | 899166.3125 | 0.82 | Medium |
| KIFAA | 5 | | 549.33951 | 275.17242 | -3.53 | 2 | 884844.25 | 1.21 | Medium |
| KIFR | 4 | | 563.36639 | 282.18607 | -2.73 | 2 | 390362.7109 | 0.8 | Medium |
| KLGAPY | 6 | | 648.37154 | 648.36981 | -2.66 | 1 | 1134047.375 | 1.27 | High |
| KLGIAG | 6 | | 558.36097 | 558.35968 | -2.32 | 1 | 1115697 | 1.39 | High |
| KIGIPQ | 6 | | 655.41374 | 655.41223 | -2.3 | 1 | 2329077 | 1.45 | High |
| KIGIQ | 5 | | 558.36097 | 558.35968 | -2.32 | 1 | 1115697 | 1.39 | High |
| KIIA | 4 | | 444.31805 | 444.31702 | -2.32 | 1 | 32436662.13 | 1.09 | Medium |
| KIIAG | 5 | | 501.33951 | 501.33856 | -1.89 | 1 | 789695.2188 | 1.15 | Medium |
| KIIGA | 5 | | 501.33951 | 501.33856 | -1.89 | 1 | 789695.2188 | 1.15 | Medium |
| KIIQ | 4 | | 501.33951 | 501.33856 | -1.89 | 1 | 789695.2188 | 1.15 | Medium |
| KIIR | 4 | | 529.38204 | 265.19391 | -2.84 | 2 | 837089.7227 | 0.81 | Medium |
| KLMPD | 5 | | 603.31706 | 603.31567 | -2.3 | 1 | 13700086.75 | 1.33 | High |
| KLMPD | 5 | 1xOxidation [M3] | 619.31197 | 619.31055 | -2.31 | 1 | 872943.125 | 0.99 | Medium |
| KINI | 4 | | 487.32386 | 487.32275 | -2.27 | 1 | 1333432.25 | 1.12 | Medium |
| KIPLK | 5 | | 598.42866 | 299.71722 | -2.49 | 2 | 948323.25 | 1.16 | Medium |
| KLQMI | 5 | 1xOxidation [M4] | 648.37491 | 648.36981 | -7.86 | 1 | 1134047.375 | 1.32 | High |
| KIRPI | 5 | | 626.43481 | 313.72034 | -2.25 | 2 | 1268644 | 0.89 | Medium |
| KISIND | 6 | | 689.38283 | 345.19403 | -2.97 | 2 | 690935.6875 | 0.83 | Medium |
| KMLKN | 5 | 1xOxidation [M2] | 649.37016 | 649.36615 | -6.17 | 1 | 1131586.5 | 1.2 | High |
| KMSVR | 5 | | 620.35484 | 310.68381 | 8.86 | 2 | | 0.87 | Medium |
| KNARIGR | 7 | | 814.50059 | 272.17142 | -1.1 | 3 | 2731247.25 | 1.05 | Medium |
| KNHDLVV | 7 | | 824.46248 | 412.73361 | -3.07 | 2 | 2230818.25 | 0.83 | Medium |
| KNPF | 4 | | 505.27691 | 505.27676 | -0.29 | 1 | 10190098.5 | 0.77 | Medium |
| KNRLCDRFL | 9 | | 1164.63062 | 388.88269 | 2.49 | 3 | 3525646.75 | 1.04 | Medium |
| KNVDLA | 6 | | 659.37227 | 659.37024 | -3.07 | 1 | 603916.375 | 0.88 | Medium |
| KPFDP | 5 | | 603.31369 | 603.31567 | 3.29 | 1 | | 0.81 | Medium |

| | | | | | | | | | | |
|---|---|---|---|---|---|---|---|---|---|---|
| KPFKN | 5 | | 633.37187 | 633.37177 | -0.17 | 1 | 2152933.084 | 0.75 | Medium |
| KPFNK | 5 | | 633.37187 | 633.37177 | -0.17 | 1 | 2152933.084 | 0.74 | Medium |
| KPGF | 4 | | 448.25545 | 448.254 | -3.23 | 1 | 277902.9375 | 0.74 | Medium |
| KPKFN | 5 | | 633.37187 | 633.37177 | -0.17 | 1 | 2152933.084 | 0.75 | Medium |
| KPKNF | 5 | | 633.37187 | 633.37177 | -0.17 | 1 | 2152933.084 | 0.75 | Medium |
| KPLDVDQ | 7 | | 814.43051 | 407.71735 | -3.8 | 2 | 933484.625 | 1.02 | Medium |
| KPLGSPESN | 9 | | 928.47344 | 464.73941 | -2.04 | 2 | 2956032.5 | 1.01 | Medium |
| KPIVR | 5 | | 612.41916 | 306.71228 | -3.06 | 2 | 1452769.375 | 0.97 | Medium |
| KPVIVR | 6 | | 711.48757 | 711.48602 | -2.18 | 1 | 748818.3125 | 0.74 | Medium |
| KPVNPI | 6 | | 667.41374 | 334.20917 | -4.01 | 2 | 2453927 | 0.84 | Medium |
| KPVNRL | 6 | | 726.46208 | 363.73364 | -2.86 | 2 | 569038.375 | 1.19 | Medium |
| KQAQIR | 6 | | 743.45225 | 372.22824 | -4.09 | 2 | 1163769 | 0.84 | Medium |
| KQFIN | 5 | | 649.36679 | 649.36615 | -0.98 | 1 | | 1.16 | Medium |
| KQLAVQLGI | 9 | | 969.60914 | 485.30679 | -2.92 | 2 | | 0.84 | Medium |
| KQIGVQLAI | 9 | | 969.60914 | 485.30679 | -2.92 | 2 | | 0.87 | Medium |
| KQLPY | 5 | | 648.37154 | 324.68848 | -2.87 | 2 | 2114230.5 | 1.05 | Medium |
| KQLQGLVIA | 9 | | 969.60914 | 485.30679 | -2.92 | 2 | | 0.87 | Medium |
| KQPGSF | 6 | | 663.34605 | 663.34393 | -3.19 | 1 | 1017285.625 | 1.36 | High |
| KQQTGKLVGR | 10 | | 1114.66912 | 372.22824 | 0.94 | 3 | 1163769 | 1.17 | Medium |
| KQRIIQ | 6 | | 785.4992 | 393.2515 | -4.44 | 2 | 708114.5625 | 1.73 | Medium |
| KRFP | 4 | | 547.33509 | 274.17044 | -2.72 | 2 | 1367851.301 | 1 | Medium |
| KRIM | 4 | | 547.33846 | 274.17044 | -8.88 | 2 | 1090970.77 | 0.95 | Medium |
| KRMI | 4 | | 547.33846 | 274.17044 | -8.88 | 2 | 1004410.816 | 0.95 | Medium |
| KRNVKLLLPVLK | 12 | | 1420.97262 | 474.32733 | -3.64 | 3 | 2093851 | 1.1 | Medium |
| KRPDVVSP | 8 | | 897.51524 | 449.26001 | -2.78 | 2 | 7547441.813 | 2.17 | High |
| KRPF | 4 | | 547.33509 | 274.17044 | -2.72 | 2 | 1090970.77 | 0.95 | Medium |
| KRSTVCYR | 8 | | 1012.53566 | 338.1813 | -6.22 | 3 | 4170621.875 | 1 | Medium |
| KSMI | 4 | | 478.26938 | 478.26801 | -2.88 | 1 | 18209.56055 | 0.75 | Medium |
| KSVI | 4 | | 446.29731 | 446.2962 | -2.48 | 1 | 3571750.156 | 1.16 | Medium |
| KTDDPELGDR | 10 | | 1145.54331 | 573.27838 | 5.39 | 2 | 712580.4375 | 0.86 | Medium |
| KTDYDFAR | 8 | | 1015.48434 | 508.24411 | -3.34 | 2 | 64892.73828 | 1.24 | Medium |
| KTFP | 4 | | 492.28166 | 492.28027 | -2.82 | 1 | | 0.71 | Medium |
| KTHINPE | 7 | | 838.44174 | 419.72855 | 9.63 | 2 | | 0.85 | Medium |
| KTIM | 4 | | 492.28503 | 492.28027 | -9.66 | 1 | | 0.71 | Medium |
| KTIQIALQ | 8 | | 914.56694 | 914.55945 | -8.19 | 1 | | 0.7 | Medium |
| KTMI | 4 | | 492.28503 | 492.28027 | -9.66 | 1 | | 0.71 | Medium |
| KTMISSR | 7 | 1xOxidation [M3] | 838.44511 | 419.72855 | 5.61 | 2 | | 0.85 | Medium |
| KTPF | 4 | | 492.28166 | 492.2803 | -2.76 | 1 | 28233070 | 1.25 | High |
| KTQFPQ | 6 | | 748.39882 | 374.70181 | -3.3 | 2 | 1524309.25 | 0.82 | Medium |
| KTQKMVA | 7 | | 805.46004 | 403.23093 | -6.78 | 2 | 3962834.5 | 0.82 | Medium |
| KTVI | 4 | | 460.31296 | 460.31168 | -2.79 | 1 | 1133458.625 | 1.21 | High |
| KTYY | 4 | | 574.28714 | 574.28516 | -3.45 | 1 | 761372.875 | 1.26 | High |
| KVDF | 4 | | 508.27657 | 508.2746 | -3.89 | 1 | 78960.69531 | 1.09 | Medium |
| KVIP | 4 | | 456.31805 | 456.31699 | -2.32 | 1 | 1086806.125 | 1.1 | Medium |
| KVNDAL | 6 | | 659.37227 | 659.37024 | -3.07 | 1 | 603916.375 | 0.92 | Medium |
| KVNDLA | 6 | | 659.37227 | 659.37024 | -3.07 | 1 | 603916.375 | 0.92 | Medium |
| KVRINP | 6 | | 726.46208 | 363.73364 | -2.86 | 2 | 569038.375 | 1.23 | Medium |
| KVSPY | 5 | | 593.32934 | 297.16779 | -1.76 | 2 | 879280.3125 | 1.07 | Medium |
| KVVY | 4 | | 508.31296 | 508.31165 | -2.59 | 1 | 6697229.75 | 0.93 | Medium |
| KWSPE | 5 | | 646.3195 | 646.31769 | -2.81 | 1 | 7947077.75 | 1.38 | High |
| KYLPS | 5 | | 607.34499 | 607.34454 | -0.73 | 1 | | 1.27 | High |
| KYLSP | 5 | | 607.34499 | 607.34454 | -0.73 | 1 | | 1.27 | High |
| IADFFS | 6 | | 699.33482 | 699.3407 | 8.41 | 1 | 786317.75 | 0.96 | Medium |
| LADGANL | 7 | | 673.35153 | 673.34955 | -2.94 | 1 | 2870663.75 | 0.96 | Medium |
| IAFSPELGKLA | 11 | | 1145.65649 | 573.33453 | 4.63 | 2 | | 0.96 | Medium |
| IAGGTGPM | 8 | | 703.34434 | 703.34473 | 0.55 | 1 | 24211666 | 1.52 | High |
| IAGGTGPM | 8 | 1xOxidation [M8] | 719.33925 | 719.33771 | -2.15 | 1 | 5126370.875 | 1.03 | Medium |
| IAGYR | 5 | | 579.32492 | 579.32349 | -2.48 | 1 | 4063479.75 | 0.84 | Medium |
| IALALTALQ | 9 | | 913.57169 | 913.57159 | -0.11 | 1 | | 0.97 | Medium |
| IALGG | 5 | | 430.26601 | 430.26477 | -2.88 | 1 | 48216220 | 0.94 | Medium |
| IALN | 4 | | 430.26601 | 430.26477 | -2.88 | 1 | 48216220 | 0.94 | Medium |
| IAIR | 4 | | 472.32419 | 236.66533 | -1.72 | 2 | 3177487.5 | 0.9 | Medium |
| IAPLGG | 6 | | 527.31877 | 527.31738 | -2.64 | 1 | 1921580.75 | 1.03 | Medium |
| IAPIN | 5 | | 527.31877 | 527.31738 | -2.64 | 1 | 1921580.75 | 1.03 | Medium |
| LATTINKLQ | 9 | | 1001.59897 | 501.30173 | -2.79 | 2 | | 0.81 | Medium |
| IAVDGG | 6 | | 531.2773 | 531.27515 | -4.06 | 1 | 3529007.25 | 0.75 | Medium |
| IAVDN | 5 | | 531.2773 | 531.27515 | -4.06 | 1 | 3529007.25 | 0.75 | Medium |
| IAVND | 5 | | 531.2773 | 531.27515 | -4.06 | 1 | 3529007.25 | 0.75 | Medium |
| LAVPM | 5 | 1xOxidation [M5] | 546.2956 | 546.29376 | -3.36 | 1 | 2840759.25 | 1.06 | Medium |
| IAWLIR | 6 | | 771.48757 | 257.83185 | -8.53 | 3 | | 1.31 | Medium |
| LCEGIR | 6 | | 690.36032 | 345.68115 | -7.67 | 2 | 16382090.81 | 0.95 | Medium |
| LDDNPSLR | 8 | | 929.46869 | 465.23764 | -0.73 | 2 | | 1.76 | Medium |
| IDEL | 4 | | 489.25551 | 489.25406 | -2.96 | 1 | 2683889.25 | 1.04 | Medium |
| IDGIE | 5 | | 546.27697 | 546.27423 | -5.01 | 1 | 3583164.25 | 0.76 | Medium |
| LDGTVV | 6 | | 603.33482 | 603.33307 | -2.9 | 1 | 1116935 | 0.7 | Medium |
| IDIAE | 5 | | 560.29262 | 560.2912 | -2.53 | 1 | 425404.5 | 1 | Medium |
| IDIGAK | 6 | | 616.36645 | 308.68582 | -3.38 | 2 | 1882979.25 | 1.38 | Medium |
| IDIK | 4 | | 488.30788 | 488.3045 | -6.9 | 1 | 5852783 | 1.15 | Medium |
| LDLPNSP | 7 | | 755.3934 | 755.39307 | -0.44 | 1 | 696330 | 0.9 | Medium |
| IDIQK | 5 | | 616.36645 | 308.68582 | -3.38 | 2 | 1882979.25 | 1.39 | Medium |
| LDRVIR | 6 | | 771.48355 | 257.83185 | -3.31 | 3 | | 1.26 | Medium |
| IDTI | 4 | | 461.26059 | 461.25949 | -2.38 | 1 | 78935728 | 0.91 | Medium |
| IDVPS | 5 | | 530.28205 | 530.28088 | -2.21 | 1 | 1436009.375 | 0.94 | Medium |

| | | | | | | | | | |
|---|---|---|---|---|---|---|---|---|---|
| IDVSP | 5 | | 530.28205 | 530.28088 | -2.21 | 1 | 1436009.375 | 0.94 | Medium |
| LEAKRPV | 7 | | 812.49886 | 406.75223 | -2.07 | 2 | 1284886.5 | 1.33 | Medium |
| IEDI | 4 | | 489.25551 | 489.25452 | -2.02 | 1 | 2683889.25 | 0.98 | Medium |
| IEEI | 4 | | 503.27116 | 503.26974 | -2.8 | 1 | 6904470.75 | 0.73 | Medium |
| IEGY | 4 | | 481.22929 | 481.22784 | -3.01 | 1 | 235788192 | 0.96 | Medium |
| LEICPL | 6 | | 687.37457 | 687.37659 | 2.93 | 1 | 1230449.375 | 0.94 | Medium |
| IEIE | 4 | | 503.27116 | 503.26721 | -7.84 | 1 | 9909028 | 0.71 | Medium |
| IESGVIN | 7 | | 731.3934 | 731.39185 | -2.12 | 1 | 6153296 | 0.88 | Medium |
| LETLDINPQVRA | 12 | | 1368.74815 | 684.87598 | -2.54 | 2 | 4034899 | 1.21 | Medium |
| LETVVN | 6 | | 674.37193 | 674.37 | -2.87 | 1 | 1020174.313 | 0.98 | Medium |
| LGATDALNL | 9 | | 887.48327 | 444.24417 | -2.49 | 2 | | 0.99 | Medium |
| LGAYDR | 6 | | 694.35187 | 347.67877 | -2.3 | 2 | 1121682.75 | 0.81 | Medium |
| IGAYR | 5 | | 579.32492 | 579.32349 | -2.48 | 1 | 9534797.75 | 0.84 | Medium |
| IGDELN | 6 | | 660.3199 | 660.31848 | -2.14 | 1 | 17306406 | 1.24 | High |
| LGGDDFDKK | 9 | | 994.484 | 497.74478 | -1.72 | 2 | 1304258.5 | 1.5 | Medium |
| LGGDLAA | 7 | | 616.33007 | 616.32733 | -4.44 | 1 | 8176471 | 0.84 | Medium |
| IGGLK | 5 | | 487.32386 | 487.32275 | -2.27 | 1 | 1539803 | 1.06 | Medium |
| LGGVSP | 6 | | 529.29804 | 529.29633 | -3.24 | 1 | 3637882.25 | 1.05 | Medium |
| LGKLGA | 6 | | 558.36097 | 558.35968 | -2.32 | 1 | 1115697 | 1.42 | High |
| IGKLQ | 5 | | 558.36097 | 558.35968 | -2.32 | 1 | 1115697 | 1.42 | High |
| IGID | 4 | | 417.23438 | 417.23343 | -2.27 | 1 | 29495884 | 0.98 | Medium |
| IGIK | 4 | | 430.3024 | 430.30127 | -2.62 | 1 | 1368010.625 | 0.84 | Medium |
| IGIR | 4 | | 458.30854 | 458.30798 | -1.22 | 1 | 17577624.63 | 1.09 | Medium |
| IGIT | 4 | | 403.25511 | 403.25415 | -2.38 | 1 | 964773.5 | 0.8 | Medium |
| IGIVM | 5 | 1xOxidation [M5] | 548.31125 | 548.31085 | -0.72 | 1 | 1267634.875 | 1.11 | Medium |
| LGRAPYDE | 8 | | 920.44722 | 460.72614 | -2.42 | 2 | 1626167.5 | 1 | Medium |
| LGRAPYDES | 9 | | 1007.47925 | 504.24271 | -1.11 | 2 | | 1.27 | Medium |
| LGRAPYDESE | 10 | | 1136.52184 | 568.76343 | -1.99 | 2 | 9529563.313 | 2.01 | High |
| LGRMPS | 6 | | 660.34976 | 660.34723 | -3.83 | 1 | 38950845.25 | 1.37 | High |
| LGRMPS | 6 | 1xOxidation [M4] | 676.34467 | 338.67499 | -2.92 | 2 | 490795.6133 | 0.81 | Medium |
| IKGGI | 5 | | 487.32386 | 487.32275 | -2.27 | 1 | 1333432.25 | 1.14 | Medium |
| IKIAG | 5 | | 501.33951 | 251.17279 | -2.4 | 2 | 770371.0625 | 0.92 | Medium |
| IKLGA | 5 | | 501.33951 | 251.17279 | -2.4 | 2 | 770371.0625 | 0.92 | Medium |
| IKIQ | 4 | | 501.33951 | 251.17279 | -2.4 | 2 | 770371.0625 | 0.92 | Medium |
| IKIR | 4 | | 529.38204 | 265.19391 | -2.84 | 2 | 768868.2578 | 0.83 | Medium |
| IKNI | 4 | | 487.32386 | 487.32275 | -2.27 | 1 | 1333432.25 | 1.14 | Medium |
| IIAGP | 5 | | 470.29731 | 470.29623 | -2.29 | 1 | 613625408 | 0.78 | Medium |
| ILANPQ | 6 | | 655.37735 | 655.37531 | -3.12 | 1 | 193208908 | 1.2 | High |
| IIAPG | 5 | | 470.29731 | 470.29623 | -2.29 | 1 | 613625408 | 0.78 | Medium |
| ILAPQN | 6 | | 655.37735 | 655.37531 | -3.12 | 1 | 193208908 | 1.2 | High |
| ILASHP | 6 | | 637.36679 | 637.36523 | -2.44 | 1 | 1397632 | 0.83 | Medium |
| LLATTH | 6 | | 655.37735 | 655.37531 | -3.12 | 1 | 156385168 | 1.21 | High |
| LLDDEDAKE | 9 | | 1047.48406 | 524.24426 | -2.68 | 2 | 3130207.25 | 1.69 | Medium |
| IIDPETR | 7 | | 843.45706 | 422.23135 | -1.93 | 2 | 2671163.25 | 1.38 | Medium |
| IIDPQ | 5 | | 585.32425 | 585.32281 | -2.46 | 1 | 1705200.125 | 0.97 | Medium |
| IIGVGG | 6 | | 515.31877 | 515.31775 | -1.99 | 1 | 521605.4688 | 0.99 | Medium |
| IIGVN | 5 | | 515.31877 | 515.31775 | -1.99 | 1 | 521605.4688 | 0.99 | Medium |
| IIKR | 4 | | 529.38204 | 265.19391 | -2.84 | 2 | 277526.1016 | 0.84 | Medium |
| IILGLSAAPG | 10 | | 911.55604 | 911.55402 | -2.22 | 1 | 4401971 | 0.81 | Medium |
| ILLSCYC | 7 | | 814.38376 | 814.39185 | 9.93 | 1 | 9278673 | 0.83 | Medium |
| LLPASH | 6 | | 637.36679 | 637.36523 | -2.44 | 1 | 3704764.5 | 0.83 | Medium |
| LLPDGNGE | 8 | | 814.39412 | 814.3902 | -4.82 | 1 | 9278673 | 0.9 | Medium |
| IIPT | 4 | | 443.28641 | 443.28503 | -3.11 | 1 | 69035384 | 0.79 | Medium |
| IIQRPDGPVASK | 12 | | 1280.73211 | 427.58148 | -1.73 | 3 | 970866.875 | 1.51 | Medium |
| IITP | 4 | | 443.28641 | 443.28503 | -3.11 | 1 | 69035384 | 0.79 | Medium |
| LIVDGS | 6 | | 603.33482 | 603.33307 | -2.9 | 1 | 1116935 | 0.71 | Medium |
| IIVGGG | 6 | | 515.31877 | 515.31775 | -1.99 | 1 | 521605.4688 | 0.98 | Medium |
| IIVGN | 5 | | 515.31877 | 515.31775 | -1.99 | 1 | 521605.4688 | 0.98 | Medium |
| LMTPPL | 6 | 1xOxidation [M2] | 687.37457 | 687.37659 | 2.93 | 1 | 1230449.375 | 0.94 | Medium |
| IMVRGI | 6 | 1xOxidation [M2] | 704.41236 | 704.4173 | 7.01 | 1 | 1421272 | 0.79 | Medium |
| INIK | 4 | | 487.32386 | 487.32275 | -2.27 | 1 | 1539803 | 1.06 | Medium |
| INLPR | 5 | | 612.38277 | 306.69406 | -3.14 | 2 | 1632788.625 | 1.08 | Medium |
| INPIRDG | 7 | | 784.43118 | 392.71817 | -2.7 | 2 | 4959336 | 1.36 | Medium |
| LNVPS | 5 | | 529.29804 | 529.29675 | -2.43 | 1 | 3637882.25 | 0.89 | Medium |
| INVSP | 5 | | 529.29804 | 529.29675 | -2.43 | 1 | 3637882.25 | 0.89 | Medium |
| IPAIN | 5 | | 527.31877 | 527.31738 | -2.64 | 1 | 1921580.75 | 1 | Medium |
| IPANDDAIR | 9 | | 984.51088 | 492.75867 | -0.84 | 2 | 2536097 | 2.02 | High |
| IPDPR | 5 | | 597.33549 | 597.3338 | -2.82 | 1 | 66527128 | 0.87 | Medium |
| LPEDEKER | 8 | | 1015.50546 | 508.25446 | -3.77 | 2 | 2537432.5 | 0.92 | Medium |
| IPETDI | 6 | | 687.35595 | 687.3573 | 1.97 | 1 | 10289165 | 0.78 | Medium |
| IPGAT | 5 | | 458.26092 | 458.25986 | -2.33 | 1 | 16555660.44 | 0.81 | Medium |
| IPGDCLLKPWN | 11 | | 1255.65036 | 628.328 | -1.29 | 2 | 418389.5313 | 0.96 | Medium |
| LPGGP | 5 | | 440.25036 | 220.62816 | -3 | 2 | 3487557.25 | 0.99 | Medium |
| LPGINT | 6 | | 614.3508 | 614.34906 | -2.84 | 1 | 499311716.9 | 1.01 | Medium |
| LPGPG | 5 | | 440.25036 | 220.62816 | -3 | 2 | 3487557.25 | 0.99 | Medium |
| IPGVGAT | 7 | | 614.3508 | 614.34906 | -2.84 | 1 | 499311716.9 | 1.01 | Medium |
| LPGYPLR | 7 | | 815.4774 | 408.23914 | -7.85 | 2 | 11456782 | 0.95 | Medium |
| IPIGG | 5 | | 456.28166 | 456.2804 | -2.77 | 1 | 9504470 | 1.1 | Medium |
| LPQGVT | 6 | | 614.3508 | 614.34906 | -2.84 | 1 | 497514912 | 0.99 | Medium |
| IPQRLLDYN | 9 | | 1131.61568 | 1131.61938 | 3.27 | 1 | 834102.3125 | 1.05 | Medium |
| LPQTGV | 6 | | 614.3508 | 614.34894 | -3.03 | 1 | 497514912 | 0.79 | Medium |
| LPRSIR | 6 | | 741.47298 | 371.23904 | -2.93 | 2 | 1681105.375 | 0.83 | Medium |
| LQPGPVH | 7 | | 747.4148 | 747.41235 | -3.27 | 1 | 10522736 | 0.84 | Medium |

| | | | | | | | | | | |
|---|---|---|---|---|---|---|---|---|---|---|
| LQPPGPE | 7 | | 737.38283 | 737.38147 | -1.85 | 1 | 953897.875 | 1.19 | Medium |
| IQYR | 4 | | 579.32492 | 579.32349 | -2.48 | 1 | 4063479.75 | 0.84 | Medium |
| LRDGVVE | 7 | | 787.43084 | 394.21732 | -4.43 | 2 | 6492929.5 | 1.59 | Medium |
| LRDGVVEE | 8 | | 916.47344 | 916.47144 | -2.18 | 1 | 8555229.125 | 1.51 | High |
| IRGDI | 5 | | 573.33549 | 287.17084 | -1.9 | 2 | 7316383 | 1.12 | Medium |
| IRSVVDWYKLP | 11 | | 1375.77325 | 688.39655 | 9.13 | 2 | | 1.04 | Medium |
| ISDVSLSIN | 9 | | 947.5044 | 474.25482 | -2.15 | 2 | 6178690 | 0.81 | Medium |
| ISFAG | 5 | | 494.26092 | 494.25946 | -2.96 | 1 | 16496116 | 0.9 | Medium |
| ISFGA | 5 | | 494.26092 | 494.25946 | -2.96 | 1 | 16496116 | 0.9 | Medium |
| LSFQ | 4 | | 494.26092 | 494.25946 | -2.96 | 1 | 16496116 | 0.9 | Medium |
| ISKPI | 5 | | 557.36572 | 557.3642 | -2.74 | 1 | 5434322.5 | 1.08 | Medium |
| ISIITIQ | 7 | | 787.49238 | 787.49036 | -2.57 | 1 | 471643.4375 | 0.83 | Medium |
| ISNLE | 5 | | 575.30352 | 575.3009 | -4.54 | 1 | 1042037.688 | 1.05 | Medium |
| ISPSDKGGKLY | 11 | | 1164.62591 | 388.88269 | 6.53 | 3 | 3525646.75 | 1.03 | Medium |
| ISPY | 4 | | 479.25003 | 479.24884 | -2.47 | 1 | 20519446 | 1.06 | Medium |
| ISPYE | 5 | | 608.29262 | 608.29163 | -1.63 | 1 | 5312555.5 | 0.86 | Medium |
| LSQLNPTRD | 9 | | 1043.548 | 522.27643 | -2.32 | 2 | 2301548.5 | 1.13 | Medium |
| LSRDY | 5 | | 653.32532 | 653.32269 | -4.01 | 1 | 2447197.75 | 0.95 | Medium |
| ISTKTL | 6 | | 662.40832 | 662.40662 | -2.57 | 1 | 7293540.5 | 0.7 | Medium |
| ISTI | 4 | | 433.26568 | 433.26413 | -3.57 | 1 | 978312.6875 | 0.7 | Medium |
| ISTIIIQ | 7 | | 787.49238 | 787.49036 | -2.57 | 1 | 471643.4375 | 0.83 | Medium |
| ISTTKI | 6 | | 662.40832 | 662.40662 | -2.57 | 1 | 7293540.5 | 0.7 | Medium |
| LSVGGA | 6 | | 503.28239 | 503.28201 | -0.75 | 1 | 6262132 | 0.73 | Medium |
| ISVGQ | 5 | | 503.28239 | 503.28201 | -0.75 | 1 | 6262132 | 0.73 | Medium |
| LSVPGA | 6 | | 543.31369 | 543.31274 | -1.74 | 1 | 2929527.5 | 1.01 | Medium |
| LSVPQ | 5 | | 543.31369 | 543.31274 | -1.74 | 1 | 2929527.5 | 1.01 | Medium |
| ITIAG | 5 | | 474.29223 | 474.2915 | -1.52 | 1 | 3862425.75 | 1.11 | Medium |
| ITLGA | 5 | | 474.29223 | 474.2915 | -1.52 | 1 | 3862425.75 | 1.11 | Medium |
| ITIQ | 4 | | 474.29223 | 474.2915 | -1.52 | 1 | 3862425.75 | 1.11 | Medium |
| ITQKLALQ | 8 | | 914.56694 | 914.55945 | -8.19 | 1 | | 0.7 | Medium |
| LTSGEV | 6 | | 605.31408 | 605.31085 | -5.34 | 1 | 26197130 | 0.82 | Medium |
| ITVGPN | 6 | | 600.33515 | 600.33319 | -3.27 | 1 | | 0.74 | Medium |
| LTVVAR | 6 | | 658.42464 | 658.42053 | -6.23 | 1 | 1067990.625 | 1.4 | High |
| LVDEFQDTN | 9 | | 1080.4844 | 540.74304 | -5.17 | 2 | 1059360.5 | 1.17 | Medium |
| IVGVT | 5 | | 488.30788 | 488.30664 | -2.53 | 1 | 12110439 | 0.89 | Medium |
| LVGVTQ | 6 | | 616.36645 | 616.3645 | -3.16 | 1 | 2233504.5 | 1.06 | Medium |
| IVIK | 4 | | 472.34935 | 472.34805 | -2.74 | 1 | 8247603.875 | 1.37 | High |
| IVIM | 4 | 1xOxidation [M4] | 491.28978 | 491.28845 | -2.71 | 1 | 1294633.875 | 0.82 | Medium |
| LVNPQIPQ | 8 | | 908.51999 | 908.52747 | 8.23 | 1 | 1627272.25 | 0.71 | Medium |
| LVSPN | 5 | | 529.29804 | 529.2959 | -4.04 | 1 | 3637882.25 | 0.77 | Medium |
| IVVE | 4 | | 459.28133 | 459.28006 | -2.76 | 1 | 12587389 | 0.71 | Medium |
| LVYPGG | 6 | | 605.32934 | 605.32831 | -1.7 | 1 | | 0.83 | Medium |
| LVYPN | 5 | | 605.32934 | 605.32831 | -1.7 | 1 | | 0.83 | Medium |
| LWKKRPD | 7 | | 942.55196 | 471.77866 | -2.04 | 2 | 156542.8594 | 1.26 | Medium |
| IYDPE | 5 | | 636.28753 | 636.28577 | -2.78 | 1 | 4423733.5 | 0.97 | Medium |
| IYDSPE | 6 | | 723.31956 | 723.31915 | -0.57 | 1 | | 1.17 | Medium |
| MEPAE | 5 | | 576.23339 | 576.23175 | -2.84 | 1 | 1166789.25 | 0.93 | Medium |
| MESLK | 5 | | 607.31197 | 607.31012 | -3.05 | 1 | 1641475.5 | 1.16 | Medium |
| MGGDI | 5 | | 492.21226 | 492.21063 | -3.31 | 1 | 1687721.75 | 0.74 | Medium |
| MGGTGGGI | 8 | 1xOxidation [M1] | 665.2923 | 665.29797 | 8.53 | 1 | 958086.5625 | 0.83 | Medium |
| MGIK | 4 | | 448.25882 | 448.25769 | -2.51 | 1 | 864771.5625 | 0.79 | Medium |
| MGIT | 4 | | 421.21153 | 421.2103 | -2.93 | 1 | 3729498.75 | 0.74 | Medium |
| MNDL | 4 | | 492.21226 | 492.21063 | -3.31 | 1 | 1687721.75 | 0.74 | Medium |
| MPKPV | 5 | | 571.32723 | 571.32581 | -2.49 | 1 | 5923912.875 | 1.63 | High |
| MQGPNY | 6 | | 709.29739 | 709.29602 | -1.93 | 1 | 8958268 | 1 | Medium |
| MQGPNYA | 7 | | 780.3345 | 780.33215 | -3.01 | 1 | 5753743.5 | 0.99 | Medium |
| MQTLPK | 6 | | 717.39637 | 359.20038 | -4.03 | 2 | 2301399.25 | 1.14 | Medium |
| MSGGKPF | 7 | 1xOxidation [M1] | 739.34434 | 739.3429 | -1.95 | 1 | 917699.25 | 1.19 | Medium |
| MVDVAR | 6 | | 690.36032 | 345.68127 | -7.31 | 2 | 15839013 | 0.88 | Medium |
| MVIR | 4 | | 518.31191 | 259.65906 | -2.08 | 2 | 1160028 | 0.92 | Medium |
| MVLRGL | 6 | 1xOxidation [M1] | 704.41236 | 704.4173 | 7.01 | 1 | 1421272 | 0.79 | Medium |
| MVNAQS | 6 | | 649.29739 | 649.29254 | -7.46 | 1 | 1109569.375 | 0.72 | Medium |
| MVRGQIM | 7 | 2xOxidation [M1; M7] | 866.42227 | 433.71725 | 5.73 | 2 | 542894.375 | 0.82 | Medium |
| MVYQ | 4 | | 540.24865 | 540.24384 | -8.9 | 1 | 18538314 | 0.7 | Medium |
| NADFR | 5 | | 622.29435 | 311.64996 | -2.73 | 2 | 272638.2813 | 0.87 | Medium |
| NADIP | 5 | | 529.26165 | 529.2608 | -1.61 | 1 | 3142693.75 | 1.09 | Medium |
| NADPL | 5 | | 529.26165 | 529.2608 | -1.61 | 1 | 3142693.75 | 1.09 | Medium |
| NAGAF | 5 | | 479.22487 | 479.22388 | -2.08 | 1 | 10988858 | 0.95 | Medium |
| NAIYE | 5 | | 609.28787 | 609.28705 | -1.34 | 1 | | 1.16 | Medium |
| NAPIYTR | 7 | | 834.44683 | 417.72665 | -0.95 | 2 | 1614922.375 | 1.53 | Medium |
| NAQPDLVRQ | 9 | | 1040.54833 | 520.77667 | -2.18 | 2 | 891621.875 | 1.77 | Medium |
| NAVPPLLAKALGEAILK | 17 | | 1718.05747 | 573.3584 | 1.85 | 3 | 954379.3125 | 1.08 | Medium |
| NDMI | 4 | | 492.21226 | 492.21063 | -3.31 | 1 | 1687721.75 | 0.76 | Medium |
| NDQAE | 5 | | 576.226 | 576.23175 | 9.99 | 1 | 1166789.25 | 0.91 | Medium |
| NDRPVRID | 8 | | 984.52212 | 492.7634 | -2.64 | 2 | 1884161.438 | 1.17 | Medium |
| NELVTT | 6 | | 676.3512 | 676.34882 | -3.52 | 1 | 1391979.5 | 0.7 | Medium |
| NGSGGG | 6 | | 448.17865 | 448.181 | 5.24 | 1 | 657374.125 | 0.99 | Medium |
| NGSGN | 5 | | 448.17865 | 448.181 | 5.24 | 1 | 657374.125 | 0.99 | Medium |
| NHKPF | 5 | | 642.33582 | 321.67065 | -2.79 | 2 | 155120.9375 | 1.02 | Medium |
| NHKSPI | 6 | | 695.3835 | 695.38184 | -2.39 | 1 | 1233173.25 | 0.78 | Medium |
| NKGKLEPGLH | 10 | | 1092.61602 | 364.87589 | -2.67 | 3 | 2295411 | 1.19 | Medium |
| NKGPKGF | 7 | | 747.4148 | 374.20993 | -2.96 | 2 | 108139569.3 | 1.25 | Medium |
| NLEEDNVGA | 9 | | 960.42688 | 960.42285 | -4.19 | 1 | 1758059.313 | 1.15 | Medium |

| | | | | | | | | | |
|---|---|---|---|---|---|---|---|---|---|
| NIENPKAV | 8 | | 884.48361 | 884.48096 | -3 | 1 | 673287.9375 | 0.84 | Medium |
| NIENPRVE | 8 | | 970.49523 | 485.74976 | -3.09 | 2 | 5337943 | 1.11 | Medium |
| NIGHY | 5 | | 603.28854 | 603.28754 | -1.66 | 1 | 653699.375 | 0.8 | Medium |
| NLKTPRYN | 8 | | 1005.5476 | 503.27698 | -0.92 | 2 | 3351656 | 1.2 | Medium |
| NIIR | 4 | | 515.33001 | 515.32867 | -2.59 | 1 | 4643888.5 | 1.19 | Medium |
| NLPRPA | 6 | | 667.38858 | 667.38531 | -4.9 | 1 | | 0.85 | Medium |
| NLRPPA | 6 | | 667.38858 | 667.38531 | -4.9 | 1 | | 0.81 | Medium |
| NIVPS | 5 | | 529.29804 | 529.29675 | -2.43 | 1 | 3637882.25 | 0.89 | Medium |
| NIVSP | 5 | | 529.29804 | 529.29675 | -2.43 | 1 | 3637882.25 | 0.89 | Medium |
| NMPHQGIYE | 9 | 1xOxidation [M2] | 1104.47787 | 552.74097 | -2.91 | 2 | 2100775.75 | 0.86 | Medium |
| NPVIVR | 6 | | 697.43554 | 697.43427 | -1.82 | 1 | 1359288.375 | 1.75 | High |
| NQAF | 4 | | 479.22487 | 479.22388 | -2.08 | 1 | 10988858 | 0.95 | Medium |
| NQWE | 4 | | 576.24125 | 576.24023 | -1.77 | 1 | 1150539.875 | 0.81 | Medium |
| NRPDAIDPA | 9 | | 968.47958 | 484.74207 | -2.82 | 2 | 1193411.75 | 1.44 | Medium |
| NSGGN | 5 | | 448.17865 | 448.181 | 5.24 | 1 | 657374.125 | 0.99 | Medium |
| NSGHPGLPM | 9 | 1xOxidation [M9] | 925.41963 | 463.21259 | -1.87 | 2 | 899622.0625 | 1.19 | Medium |
| NSGREEPGGNL | 11 | | 1129.52324 | 565.26306 | -3.89 | 2 | 1621128.625 | 1.7 | Medium |
| NSIAVD | 6 | | 618.30933 | 618.30823 | -1.79 | 1 | 2795657.75 | 0.7 | Medium |
| NSLDLG | 6 | | 618.30933 | 618.30823 | -1.79 | 1 | 2795657.75 | 0.7 | Medium |
| NSIIDG | 6 | | 618.30933 | 618.30823 | -1.79 | 1 | 2795657.75 | 0.7 | Medium |
| NSLIGD | 6 | | 618.30933 | 618.30823 | -1.79 | 1 | 2795657.75 | 0.7 | Medium |
| NSLVDA | 6 | | 618.30933 | 618.30823 | -1.79 | 1 | 2795657.75 | 0.7 | Medium |
| NSPLNVLRALH | 11 | | 1233.70623 | 617.35767 | 1.48 | 2 | | 1.08 | Medium |
| NTGIY | 5 | | 567.2773 | 567.27582 | -2.62 | 1 | 913628.625 | 0.79 | Medium |
| NVDVTAP | 7 | | 715.3621 | 715.36011 | -2.78 | 1 | 3876684.75 | 0.76 | Medium |
| NVIPQ | 5 | | 570.32459 | 570.32288 | -3 | 1 | 1038788 | 1.09 | Medium |
| NVRPI | 5 | | 598.36712 | 598.36554 | -2.64 | 1 | 36809244 | 1.01 | Medium |
| NYPIR | 5 | | 662.36204 | 331.68384 | -2.47 | 2 | 14522127 | 1.24 | Medium |
| PAATF | 5 | | 506.26092 | 506.25912 | -3.56 | 1 | 3227605.5 | 1.27 | High |
| PCMQLAY | 7 | | 825.36336 | 825.36475 | 1.68 | 1 | 1920656.75 | 0.71 | Medium |
| PDAGL | 5 | | 472.24019 | 472.23923 | -2.04 | 1 | 2332819.75 | 0.77 | Medium |
| PDGGI | 5 | | 458.22454 | 458.22339 | -2.51 | 1 | 592150.625 | 0.79 | Medium |
| PDGIVGQE | 8 | | 814.39412 | 814.39313 | -1.22 | 1 | 1292029.375 | 0.72 | Medium |
| PDIEI | 5 | | 586.30827 | 586.30688 | -2.36 | 1 | 6733985 | 0.89 | Medium |
| PDLPK | 5 | | 569.32934 | 569.32813 | -2.13 | 1 | 4732611.5 | 0.85 | Medium |
| PDQI | 4 | | 472.24019 | 472.23923 | -2.04 | 1 | 2332819.75 | 0.77 | Medium |
| PDSDSGL | 7 | | 690.29408 | 690.29547 | 2.02 | 1 | 715211.75 | 0.71 | Medium |
| PEQIE | 5 | | 615.29843 | 615.29736 | -1.74 | 1 | 168858.1875 | 0.77 | Medium |
| PGAQVY | 6 | | 634.3195 | 634.31781 | -2.67 | 1 | 7545662.5 | 1.14 | Medium |
| PGDGI | 5 | | 458.22454 | 458.22339 | -2.51 | 1 | 592150.625 | 0.79 | Medium |
| PGFAR | 5 | | 547.29871 | 547.297 | -3.12 | 1 | 4702021.25 | 1.26 | High |
| PGKKGF | 6 | | 633.37187 | 317.18893 | -2.02 | 2 | 2078618.5 | 1.02 | Medium |
| PGNSFAG | 7 | | 649.29402 | 649.29254 | -2.27 | 1 | 1109569.375 | 0.73 | Medium |
| PHAP | 4 | | 421.21939 | 421.21841 | -2.33 | 1 | 31926316 | 1.07 | Medium |
| PKGF | 4 | | 448.25545 | 448.254 | -3.23 | 1 | 277902.9375 | 0.75 | Medium |
| PKIR | 4 | | 513.35074 | 257.17838 | -2.47 | 2 | 2298355.25 | 0.82 | Medium |
| PKNPAGF | 7 | | 730.38825 | 365.69666 | -3.03 | 2 | 1870514.391 | 1.09 | Medium |
| PLAIGG | 6 | | 527.31877 | 527.31738 | -2.64 | 1 | 1921580.75 | 1 | Medium |
| PIALN | 5 | | 527.31877 | 527.31738 | -2.64 | 1 | 1921580.75 | 1 | Medium |
| PLDPR | 5 | | 597.33549 | 597.3338 | -2.82 | 1 | 66527128 | 0.87 | Medium |
| PLETID | 6 | | 687.35595 | 687.3573 | 1.97 | 1 | 10289165 | 0.78 | Medium |
| PLETVE | 6 | | 687.35595 | 687.3573 | 1.97 | 1 | 10289165 | 0.78 | Medium |
| PLFTLELAPGLG | 12 | | 1227.69835 | 1227.69141 | -5.66 | 1 | 21746320 | 0.82 | Medium |
| PIGAT | 5 | | 458.26092 | 458.25989 | -2.26 | 1 | 16555660.44 | 1.17 | Medium |
| PLGPG | 5 | | 440.25036 | 220.62816 | -3 | 2 | 3487557.25 | 0.99 | Medium |
| PIGVGLSRRF | 10 | | 1101.65274 | 1101.65015 | -2.35 | 1 | 870658.1875 | 0.96 | Medium |
| PLYLSILESL | 10 | | 1147.6609 | 574.33685 | 4.82 | 2 | | 0.94 | Medium |
| PNALASVSP | 9 | | 855.45706 | 428.23196 | -0.48 | 2 | 1223288 | 0.82 | Medium |
| PPTEAL | 6 | | 627.33482 | 627.33307 | -2.79 | 1 | 8535896 | 0.83 | Medium |
| PPTEPYPQ | 8 | | 928.44107 | 928.43433 | -7.27 | 1 | 1147300 | 0.83 | Medium |
| PQEIE | 5 | | 615.29843 | 615.29736 | -1.74 | 1 | 168858.1875 | 0.77 | Medium |
| PQTNTL | 6 | | 673.35153 | 673.35107 | -0.68 | 1 | 2870663.75 | 0.95 | Medium |
| PSEIAAG | 7 | | 644.32498 | 644.32288 | -3.27 | 1 | 12489831 | 0.91 | Medium |
| PSEIQ | 5 | | 573.28787 | 573.28613 | -3.03 | 1 | 4079337.25 | 0.86 | Medium |
| PSMEIM | 6 | 2xOxidation [M3; M6] | 739.30009 | 739.29724 | -3.85 | 1 | 2400936.5 | 0.88 | Medium |
| PTPEAI | 6 | | 627.33482 | 627.33307 | -2.79 | 1 | 8535896 | 0.83 | Medium |
| PVAATL | 6 | | 571.34499 | 571.34357 | -2.49 | 1 | 3631437.75 | 0.81 | Medium |
| PVATAI | 6 | | 571.34499 | 571.34357 | -2.49 | 1 | 3649164.422 | 0.8 | Medium |
| PVAVSV | 6 | | 571.34499 | 571.34351 | -2.6 | 1 | 3631437.75 | 0.73 | Medium |
| PVAVVEE | 7 | | 742.39815 | 742.39905 | 1.21 | 1 | 28593014.5 | 1.18 | Medium |
| PVEPR | 5 | | 597.33549 | 597.33325 | -3.74 | 1 | 66527128 | 0.72 | Medium |
| PVSV | 4 | | 401.23946 | 401.23825 | -3.02 | 1 | 54510248 | 0.85 | Medium |
| QAAF | 4 | | 436.21906 | 436.21741 | -3.79 | 1 | 1911378.125 | 1.07 | Medium |
| QDILE | 5 | | 617.31408 | 617.31274 | -2.17 | 1 | 796395.125 | 0.93 | Medium |
| QDPVLS | 6 | | 658.34063 | 658.33923 | -2.12 | 1 | 2739944 | 1.09 | Medium |
| QEGF | 4 | | 480.20889 | 480.20782 | -2.22 | 1 | 12471353 | 0.74 | Medium |
| QEGGF | 5 | | 537.23035 | 537.22772 | -4.9 | 1 | 1004501.438 | 0.75 | Medium |
| QEMNY | 5 | | 684.26575 | 684.26331 | -3.58 | 1 | 1081722.625 | 1.27 | High |
| QENF | 4 | | 537.23035 | 537.22772 | -4.9 | 1 | 1004501.438 | 0.75 | Medium |
| QEPIE | 5 | | 615.29843 | 615.29736 | -1.74 | 1 | 168858.1875 | 0.77 | Medium |
| QGDIP | 5 | | 529.26165 | 529.2608 | -1.61 | 1 | 3142693.75 | 1.09 | Medium |
| QGDPI | 5 | | 529.26165 | 529.2608 | -1.61 | 1 | 3142693.75 | 1.09 | Medium |
| QGGAF | 5 | | 479.22487 | 479.22382 | -2.21 | 1 | 13290485.25 | 1.15 | Medium |

| | | | | | | | | | |
|---|---|---|---|---|---|---|---|---|---|
| QGIYE | 5 | | 609.28787 | 609.28705 | -1.34 | 1 | | 1.16 | Medium |
| QGMY | 4 | | 498.2017 | 498.20056 | -2.28 | 1 | 25821560 | 1.07 | Medium |
| QGMY | 4 | 1xOxidation [M3] | 514.19661 | 514.1955 | -2.17 | 1 | 1333277.5 | 1.01 | Medium |
| QIDI | 4 | | 488.27149 | 488.27075 | -1.51 | 1 | 1568978.75 | 0.7 | Medium |
| QIEAV | 5 | | 559.3086 | 559.30695 | -2.96 | 1 | 23551980 | 0.75 | Medium |
| QLEGI | 5 | | 559.3086 | 559.30695 | -2.96 | 1 | 23551980 | 0.75 | Medium |
| QLELG | 5 | | 559.3086 | 559.30695 | -2.96 | 1 | 23551980 | 0.75 | Medium |
| QIEVA | 5 | | 559.3086 | 559.30695 | -2.96 | 1 | 23551980 | 0.75 | Medium |
| QIIE | 4 | | 502.28714 | 502.28705 | -0.18 | 1 | 1159842.25 | 0.99 | Medium |
| QINGSALCGK | 10 | | 990.50369 | 495.75189 | -7.25 | 2 | 1287403 | 0.96 | Medium |
| QMRVYPN | 7 | | 907.44545 | 454.22852 | 4.75 | 2 | | 0.82 | Medium |
| QNAF | 4 | | 479.22487 | 479.22382 | -2.21 | 1 | 10988858 | 1.03 | Medium |
| QNWE | 4 | | 576.24125 | 576.24023 | -1.77 | 1 | 1150539.875 | 0.85 | Medium |
| QPELE | 5 | | 615.29843 | 615.29736 | -1.74 | 1 | 168858.1875 | 0.77 | Medium |
| QPIAENLKDYEVRVRQ | 16 | | 1958.0454 | 653.35925 | 9.09 | 3 | 2297855.25 | 1.11 | Medium |
| QSGIY | 5 | | 567.2773 | 567.27582 | -2.62 | 1 | 913628.625 | 0.79 | Medium |
| QTALP | 5 | | 529.29804 | 529.29645 | -3.01 | 1 | 1138546.875 | 0.75 | Medium |
| QTAPI | 5 | | 529.29804 | 529.29645 | -3.01 | 1 | 1138546.875 | 0.75 | Medium |
| QTLSDLE | 7 | | 805.39379 | 805.39142 | -2.94 | 1 | 2220512 | 1.28 | High |
| QTNQPQ | 6 | | 715.33694 | 358.17081 | -3.64 | 2 | | 0.81 | Medium |
| QVATGPM | 7 | 1xOxidation [M7] | 719.33925 | 719.33771 | -2.15 | 1 | 5126370.875 | 1.03 | Medium |
| QVLAPR | 6 | | 683.41989 | 342.21271 | -2.56 | 2 | 479742.125 | 0.92 | Medium |
| QVVSP | 5 | | 529.29804 | 529.29675 | -2.43 | 1 | 3637882.25 | 0.89 | Medium |
| RAMNRP | 6 | 1xOxidation [M3] | 760.38827 | 380.6951 | -7.03 | 2 | 259371.3594 | 1.16 | Medium |
| RASHYLR | 7 | | 902.49551 | 451.75003 | -3.02 | 2 | | 0.98 | Medium |
| RAVLE | 5 | | 587.35114 | 587.35034 | -1.35 | 1 | 6275274.5 | 0.74 | Medium |
| RAVMLY | 6 | 1xOxidation [M4] | 768.40727 | 768.41003 | 3.59 | 1 | 1196482.625 | 0.71 | Medium |
| RAWE | 4 | | 561.27797 | 561.27618 | -3.19 | 1 | 769796.375 | 1.07 | Medium |
| RDGGIP | 6 | | 614.32565 | 614.32477 | -1.44 | 1 | 5049779.875 | 1.28 | High |
| RDGGQNV | 7 | | 745.35874 | 373.18192 | -2.93 | 2 | | 0.86 | Medium |
| RDHRIG | 6 | | 753.41145 | 753.41217 | 0.96 | 1 | 1975936.5 | 0.85 | Medium |
| RDIP | 4 | | 500.28272 | 500.28177 | -1.91 | 1 | 502633.6563 | 0.77 | Medium |
| RDNILLILQQ | 10 | | 1225.7263 | 613.36499 | -2.93 | 2 | 7860551 | 0.81 | Medium |
| RDNIP | 5 | | 614.32565 | 614.32477 | -1.44 | 1 | 5049779.875 | 1.28 | High |
| RDNPL | 5 | | 614.32565 | 614.32477 | -1.44 | 1 | 5049779.875 | 1.28 | High |
| RDPI | 4 | | 500.28272 | 500.28177 | -1.91 | 1 | 502633.6563 | 0.77 | Medium |
| RDTF | 4 | | 538.26199 | 538.26062 | -2.54 | 1 | 1934508.219 | 0.98 | Medium |
| RDVYQR | 6 | | 836.43733 | 418.72131 | -2.36 | 2 | 582240.875 | 1.1 | Medium |
| REEPGGNL | 8 | | 871.42682 | 436.21555 | -3.45 | 2 | 1443522.875 | 1.5 | Medium |
| REGGDVIREA | 10 | | 1101.56471 | 551.28333 | -4.85 | 2 | 1642333.625 | 1.6 | Medium |
| REGNDLYNE | 9 | | 1109.48579 | 555.24524 | -2.33 | 2 | 1294089.875 | 1.91 | High |
| RELLERGFVAQ | 11 | | 1317.72736 | 659.37024 | 4.43 | 2 | | 0.92 | Medium |
| REIVKSAAPPQ | 11 | | 1195.67935 | 399.2348 | 8.79 | 3 | 57751672 | 1.07 | Medium |
| REIVVKPG | 8 | | 897.55163 | 449.27835 | -2.45 | 2 | 1255459.375 | 0.94 | Medium |
| RFIGR | 5 | | 648.394 | 324.69986 | -2.41 | 2 | 5862604.063 | 1.82 | Medium |
| RFVPQ | 5 | | 646.36712 | 646.36609 | -1.6 | 1 | 42753632 | 1.6 | High |
| RFYPL | 5 | | 695.38752 | 695.38184 | -8.18 | 1 | 1233173.25 | 0.76 | Medium |
| RGFR | 4 | | 535.30994 | 268.15802 | -2.2 | 2 | 961049 | 0.83 | Medium |
| RGLKPV | 6 | | 669.44062 | 335.22296 | -2.95 | 2 | 609836.9375 | 0.82 | Medium |
| RGIIE | 5 | | 587.35114 | 587.35034 | -1.35 | 1 | 6275274.5 | 0.75 | Medium |
| RKLVASSTLVNQ | 12 | | 1315.76922 | 658.38367 | -6.97 | 2 | | 0.94 | Medium |
| RIADGY | 6 | | 694.35187 | 694.35095 | -1.31 | 1 | | 1.18 | Medium |
| RIDLVRILVG | 10 | | 1153.74155 | 385.25485 | 7.32 | 3 | 24641.89648 | 1.06 | Medium |
| RLDRSFDEN | 9 | | 1151.54398 | 384.52213 | 6.81 | 3 | 471060.0938 | 1.05 | Medium |
| RLGDTVPN | 8 | | 871.46321 | 436.23413 | -2.55 | 2 | 819619.4375 | 1.39 | Medium |
| RIGIE | 5 | | 587.35114 | 587.35034 | -1.35 | 1 | 6275274.5 | 0.74 | Medium |
| RIIA | 4 | | 472.32419 | 472.32281 | -2.92 | 1 | 2282729.25 | 0.86 | Medium |
| RIVGPG | 6 | | 598.36712 | 598.36554 | -2.64 | 1 | 36809244 | 0.82 | Medium |
| RLVNP | 5 | | 598.36712 | 598.36554 | -2.64 | 1 | 36809244 | 0.82 | Medium |
| RLVPGG | 6 | | 598.36712 | 598.36554 | -2.64 | 1 | 36809244 | 0.82 | Medium |
| RIYAP | 5 | | 619.35622 | 619.35468 | -2.5 | 1 | 1696652.375 | 1.12 | Medium |
| RIYPA | 5 | | 619.35622 | 619.35468 | -2.5 | 1 | 1696652.375 | 1.12 | Medium |
| RNTFRAGFISG | 11 | | 1225.64363 | 409.21841 | -2.4 | 3 | 819866.875 | 1.01 | Medium |
| RPFVYR | 6 | | 837.47298 | 419.2373 | -6.75 | 2 | | 1 | Medium |
| RRPW | 4 | | 614.35214 | 614.34924 | -4.72 | 1 | 497514912 | 0.7 | Medium |
| RRVI | 4 | | 543.37254 | 272.18924 | -2.46 | 2 | 1290081.625 | 0.84 | Medium |
| RRVLPQ | 6 | | 768.48388 | 384.7446 | -2.55 | 2 | 2626928 | 0.81 | Medium |
| RRWP | 4 | | 614.35214 | 614.34924 | -4.72 | 1 | 497514912 | 0.7 | Medium |
| RSDLIGR | 7 | | 816.46863 | 408.7366 | -3.3 | 2 | 4718835 | 2.51 | High |
| RSTNPL | 6 | | 687.37841 | 344.19202 | -2.41 | 2 | 956194.875 | 1.07 | Medium |
| RTGSDPLE | 8 | | 874.42649 | 437.71567 | -2.78 | 2 | 931568.9375 | 1.37 | Medium |
| RVAIE | 5 | | 587.35114 | 294.17896 | -0.86 | 2 | 7112860.625 | 1.04 | Medium |
| RVDIE | 5 | | 631.34097 | 631.33826 | -4.29 | 1 | 1327178.125 | 1.14 | Medium |
| RVDIR | 5 | | 658.39948 | 329.70251 | -2.63 | 2 | 365373.6875 | 0.87 | Medium |
| RVELVR | 6 | | 771.48355 | 386.24451 | -2.35 | 2 | 128012.2734 | 0.95 | Medium |
| RVFE | 4 | | 550.29837 | 550.29712 | -2.28 | 1 | 1558632.625 | 0.81 | Medium |
| RVRI | 4 | | 543.37254 | 272.18924 | -2.46 | 2 | 1290081.625 | 0.84 | Medium |
| RVSPQPGVPPEE | 12 | | 1291.66409 | 646.33496 | -1.12 | 2 | 5682075.125 | 1.67 | Medium |
| RVVPVRPE | 8 | | 951.57343 | 476.28943 | -1.94 | 2 | 1493565.375 | 1.72 | Medium |
| RVVVVQ | 6 | | 699.45119 | 350.22791 | -3.79 | 2 | 1692656.25 | 1.66 | Medium |
| RVYPDAPTAS | 10 | | 1076.5371 | 538.77124 | -1.76 | 2 | 1278156.375 | 1.43 | Medium |
| RWGKP | 5 | | 643.36746 | 322.18646 | -2.81 | 2 | 4698816 | 1.21 | Medium |
| RWHYR | 5 | | 817.42162 | 409.21841 | 9.71 | 2 | | 0.88 | Medium |

| | | | | | | | | | |
|---|---|---|---|---|---|---|---|---|---|
| RYETLR | 6 | | 837.45773 | 419.23309 | 1.41 | 2 | 1515668.5 | 1.23 | Medium |
| RYHPDLK | 7 | | 928.49993 | 310.17053 | -3.1 | 3 | 692802.875 | 1.54 | Medium |
| RYPGRM | 6 | | 779.3981 | 390.20154 | -2.96 | 2 | 1052796.125 | 1.47 | Medium |
| RYPYR | 5 | | 754.39948 | 377.70209 | -3.43 | 2 | 487475 | 1.12 | Medium |
| SAAAIP | 6 | | 529.29804 | 529.29645 | -3.01 | 1 | 1138546.875 | 0.75 | Medium |
| SAAAPL | 6 | | 529.29804 | 529.29645 | -3.01 | 1 | 1138546.875 | 0.75 | Medium |
| SAGGLY | 6 | | 567.2773 | 567.27582 | -2.62 | 1 | 913628.625 | 0.79 | Medium |
| SDQELAY | 7 | | 825.36249 | 825.36475 | 2.73 | 1 | 1920656.75 | 0.71 | Medium |
| SDRNPL | 6 | | 701.35768 | 701.3562 | -2.11 | 1 | 21498759.25 | 1.24 | High |
| SEDGRF | 6 | | 710.31039 | 355.65775 | -3.07 | 2 | 1703334.75 | 1.15 | Medium |
| SEDLV | 5 | | 562.27188 | 562.27045 | -2.56 | 1 | 896799.0625 | 0.77 | Medium |
| SEDVI | 5 | | 562.27188 | 562.27045 | -2.56 | 1 | 896799.0625 | 0.76 | Medium |
| SEEDPTFR | 8 | | 980.43197 | 490.71869 | -1.9 | 2 | 2847355 | 1.99 | High |
| SEGDRF | 6 | | 710.31039 | 355.65775 | -3.07 | 2 | 1703334.75 | 1.15 | Medium |
| SENNVDIA | 8 | | 861.39485 | 861.39362 | -1.44 | 1 | 1518853.5 | 1.17 | Medium |
| SERIIHNPE | 9 | | 1094.5589 | 365.52353 | -2.62 | 3 | 898210.375 | 1.89 | Medium |
| SEYIE | 5 | | 640.28245 | 640.28125 | -1.87 | 1 | 986687.25 | 0.72 | Medium |
| SFPQPTACAR | 10 | | 1077.51459 | 359.84204 | -2.8 | 3 | 2327124.188 | 1 | Medium |
| SFVGA | 5 | | 480.24527 | 480.24368 | -3.31 | 1 | | 0.76 | Medium |
| SFVQ | 4 | | 480.24527 | 480.24368 | -3.31 | 1 | | 0.76 | Medium |
| SGGGGGG | 7 | | 448.17865 | 448.181 | 5.24 | 1 | 657374.125 | 0.99 | Medium |
| SGGGGN | 6 | | 448.17865 | 448.181 | 5.24 | 1 | 657374.125 | 0.99 | Medium |
| SGIY | 4 | | 439.21873 | 439.21759 | -2.59 | 1 | 3598803 | 0.77 | Medium |
| SGNGN | 5 | | 448.17865 | 448.181 | 5.24 | 1 | 657374.125 | 0.99 | Medium |
| SKFPE | 5 | | 607.3086 | 304.15704 | -2.95 | 2 | 2665487 | 0.94 | Medium |
| SKVPY | 5 | | 593.32934 | 297.16779 | -1.76 | 2 | 879280.3125 | 1.02 | Medium |
| SLELTSAIN | 9 | | 947.5044 | 474.25482 | -2.15 | 2 | 6178690 | 0.81 | Medium |
| SIFAG | 5 | | 494.26092 | 494.25946 | -2.96 | 1 | 16496116 | 0.9 | Medium |
| SIFQ | 4 | | 494.26092 | 494.25946 | -2.96 | 1 | 16496116 | 0.9 | Medium |
| SIGYYGKKQ | 9 | | 1043.55202 | 522.27643 | -6.17 | 2 | 2301548.5 | 1.08 | Medium |
| SLLITLQ | 7 | | 787.49238 | 787.49036 | -2.57 | 1 | 471643.4375 | 0.83 | Medium |
| SLIMDGWQ | 8 | | 949.44478 | 949.44922 | 4.68 | 1 | 762453.5 | 0.79 | Medium |
| SLIWCEAQ | 8 | | 949.44478 | 949.44922 | 4.68 | 1 | 762453.5 | 0.79 | Medium |
| SINKPIYNH | 9 | | 1085.57382 | 543.28949 | -1.95 | 2 | 2788580.75 | 1.16 | Medium |
| SINIE | 5 | | 575.30352 | 575.3009 | -4.54 | 1 | 1042037.688 | 1.05 | Medium |
| SIPLEE | 6 | | 687.35595 | 687.3573 | 1.97 | 1 | 10289165 | 0.77 | Medium |
| SLPVDQDE | 8 | | 902.41017 | 902.40759 | -2.85 | 1 | 947462.3125 | 0.77 | Medium |
| SIQPVAPS | 8 | | 798.43559 | 798.43427 | -1.67 | 1 | 8522067.875 | 1.45 | High |
| SIRDY | 5 | | 653.32532 | 327.16577 | -1.61 | 2 | 5229796 | 1.16 | Medium |
| SITI | 4 | | 433.26568 | 433.26413 | -3.57 | 1 | 978312.6875 | 0.7 | Medium |
| SIVGAG | 6 | | 503.28239 | 503.28201 | -0.75 | 1 | 6262132 | 0.73 | Medium |
| SLVGGA | 6 | | 503.28239 | 503.28201 | -0.75 | 1 | 6262132 | 0.73 | Medium |
| SIVGQ | 5 | | 503.28239 | 503.28201 | -0.75 | 1 | 6262132 | 0.73 | Medium |
| SIVPAG | 6 | | 543.31369 | 543.31274 | -1.74 | 1 | 2929527.5 | 1.01 | Medium |
| SIVPQ | 5 | | 543.31369 | 543.31274 | -1.74 | 1 | 2929527.5 | 1.01 | Medium |
| SNGGGG | 6 | | 448.17865 | 448.181 | 5.24 | 1 | 657374.125 | 0.99 | Medium |
| SNGGN | 5 | | 448.17865 | 448.181 | 5.24 | 1 | 657374.125 | 0.99 | Medium |
| SNIIGD | 6 | | 618.30933 | 618.30823 | -1.79 | 1 | 2795657.75 | 0.7 | Medium |
| SNLPTS | 6 | | 618.30933 | 618.30823 | -1.79 | 1 | 2795657.75 | 0.7 | Medium |
| SNLVAD | 6 | | 618.30933 | 618.30823 | -1.79 | 1 | 2795657.75 | 0.7 | Medium |
| SNLVEG | 6 | | 618.30933 | 618.30823 | -1.79 | 1 | 2795657.75 | 0.7 | Medium |
| SNLVGE | 6 | | 618.30933 | 618.30823 | -1.79 | 1 | 2795657.75 | 0.7 | Medium |
| SPEIAG | 6 | | 573.28787 | 573.28613 | -3.03 | 1 | 4079337.25 | 0.86 | Medium |
| SPEIAQ | 6 | | 644.32498 | 644.32288 | -3.27 | 1 | 12489831 | 0.91 | Medium |
| SPEIQ | 5 | | 573.28787 | 573.28613 | -3.03 | 1 | 4079337.25 | 0.86 | Medium |
| SPELQA | 6 | | 644.32498 | 644.32288 | -3.27 | 1 | 12489831 | 0.91 | Medium |
| SPGATL | 6 | | 545.29295 | 545.29095 | -3.67 | 1 | 2062009.375 | 1.27 | High |
| SPGEI | 5 | | 502.25075 | 502.24945 | -2.6 | 1 | 10207454 | 1.26 | High |
| SPVVIK | 6 | | 642.41849 | 321.7121 | -2.44 | 2 | 1215099.25 | 1.32 | Medium |
| SQDAVHY | 7 | | 819.36316 | 410.1842 | -2.47 | 2 | 850892.25 | 0.8 | Medium |
| SQDDFEQALQ | 10 | | 1180.51167 | 590.75623 | -5.5 | 2 | 1454072.5 | 1.31 | Medium |
| SQGIY | 5 | | 567.2773 | 567.27582 | -2.62 | 1 | 913628.625 | 0.79 | Medium |
| SQPENF | 6 | | 721.31515 | 721.31213 | -4.17 | 1 | 3219601.5 | 0.75 | Medium |
| SQYGIR | 6 | | 723.37841 | 362.19226 | -1.62 | 2 | 2117392 | 1.33 | Medium |
| SRHVVV | 6 | | 696.41513 | 348.71033 | -2.52 | 2 | 991774.8125 | 1.16 | Medium |
| SSPICPA | 7 | | 674.31779 | 674.31604 | -2.59 | 1 | | 1.15 | Medium |
| SSRIM | 5 | | 593.30756 | 297.15677 | -2.19 | 2 | 443067.5313 | 0.9 | Medium |
| SSWDGAY | 7 | | 785.31006 | 785.31641 | 8.08 | 1 | 1372540.375 | 0.7 | Medium |
| SSYPNRPVPD | 10 | | 1131.54291 | 566.27448 | -1.1 | 2 | 528933 | 0.95 | Medium |
| STDGGKSW | 8 | | 837.37372 | 419.18948 | -2.43 | 2 | 1025462.813 | 0.95 | Medium |
| STLPHPA | 7 | | 722.38317 | 722.38208 | -1.5 | 1 | | 1.37 | High |
| STIVPQ | 6 | | 644.36137 | 644.36053 | -1.29 | 1 | 1216345 | 0.96 | Medium |
| STRSSAVYALRYIPQ | 15 | | 1711.91259 | 571.30963 | 1.02 | 3 | 1250706 | 1.04 | Medium |
| STTDF | 5 | | 570.24058 | 570.23962 | -1.68 | 1 | 2705750.5 | 0.83 | Medium |
| SVDLGRT | 7 | | 747.39954 | 374.20264 | -2.07 | 2 | 1301514.25 | 1.29 | Medium |
| SVDVM | 5 | | 550.25412 | 550.24927 | -8.83 | 1 | 2658656.5 | 0.75 | Medium |
| SWVGKPK | 7 | | 801.46175 | 401.23331 | -3.01 | 2 | 768517.75 | 1.04 | Medium |
| SYPGIK | 6 | | 664.36645 | 664.36401 | -3.67 | 1 | 12256841.5 | 1.45 | High |
| SYPNRPVPD | 9 | | 1044.51088 | 522.75763 | -2.78 | 2 | 1460487.875 | 1.05 | Medium |
| SYVVT | 5 | | 568.2977 | 568.29626 | -2.53 | 1 | 958665.75 | 0.77 | Medium |
| TAIAQLPEL | 9 | | 955.54587 | 955.54181 | -4.25 | 1 | 865427.125 | 0.75 | Medium |
| TALIALAQL | 9 | | 913.57169 | 913.57159 | -0.11 | 1 | | 0.99 | Medium |
| TDAAPL | 6 | | 587.30352 | 587.30237 | -1.96 | 1 | 3192551.75 | 0.96 | Medium |

| | | | | | | | | | | |
|---|---|---|---|---|---|---|---|---|---|---|
| TDDIV | 5 | | 562.27188 | 562.27045 | -2.56 | 1 | 896799.0625 | 0.77 | Medium |
| TDDVL | 5 | | 562.27188 | 562.27045 | -2.56 | 1 | 896799.0625 | 0.76 | Medium |
| TDFVAR | 6 | | 708.36752 | 354.68658 | -2.29 | 2 | 926716.375 | 1.72 | Medium |
| TDPPGANLSAAN | 12 | | 1127.53274 | 564.26843 | -2.8 | 2 | 2008531.875 | 0.84 | Medium |
| TDTPQLR | 7 | | 830.43666 | 415.72058 | -3.34 | 2 | 1748187.5 | 0.85 | Medium |
| TDVIYK | 6 | | 738.40323 | 738.40149 | -2.36 | 1 | 7196030 | 2 | High |
| TEHPHGL | 7 | | 790.38423 | 790.38251 | -2.18 | 1 | 7594834.438 | 1.12 | Medium |
| TEIVVK | 6 | | 688.42397 | 344.71451 | -3.24 | 2 | 1025552.375 | 1.07 | Medium |
| TERPREL | 7 | | 900.48976 | 300.83395 | -2.72 | 3 | 2987062 | 1.2 | Medium |
| TETDFRT | 7 | | 869.39994 | 435.20215 | -3.36 | 2 | 699538.75 | 0.99 | Medium |
| TFEPE | 5 | | 622.27188 | 622.27283 | 1.52 | 1 | 1366217 | 0.74 | Medium |
| TFQGPPH | 7 | | 783.37841 | 392.19183 | -2.58 | 2 | 650789 | 0.89 | Medium |
| TGGIY | 5 | | 510.25584 | 510.25339 | -4.81 | 1 | 1668234.125 | 0.87 | Medium |
| TGGSPL | 6 | | 531.2773 | 531.276 | -2.45 | 1 | 13971215 | 0.84 | Medium |
| TGKAPKFG | 8 | | 805.45666 | 805.45526 | -1.74 | 1 | 4314097.656 | 0.99 | Medium |
| TGSIQPVAPS | 10 | | 956.50474 | 956.50305 | -1.76 | 1 | 4236703.25 | 1.2 | High |
| TKPF | 4 | | 492.28166 | 246.64383 | -2.6 | 2 | 10614062 | 0.86 | Medium |
| TIDGLRFWLS | 10 | | 1207.64698 | 604.32922 | 3.47 | 2 | | 0.89 | Medium |
| TIGVIR | 6 | | 658.42464 | 329.71451 | -4.4 | 2 | | 1.13 | Medium |
| TLIGA | 5 | | 474.29223 | 474.29086 | -2.87 | 1 | 3862425.75 | 0.78 | Medium |
| TLPHPA | 6 | | 635.35114 | 635.34961 | -2.4 | 1 | 71124058 | 1.52 | High |
| TISGEV | 6 | | 605.31408 | 605.31085 | -5.34 | 1 | 26197130 | 0.82 | Medium |
| TITLKLPTVK | 10 | | 1113.72417 | 557.36334 | -4.28 | 2 | 1076856.875 | 1.04 | Medium |
| TLTNPQ | 6 | | 673.35153 | 673.35107 | -0.68 | 1 | 2870663.75 | 0.92 | Medium |
| TIVVRA | 6 | | 658.42464 | 658.42053 | -6.23 | 1 | 1067990.625 | 1.33 | High |
| TMLC | 4 | | 467.19925 | 467.19824 | -2.16 | 1 | 3588067.75 | 0.73 | Medium |
| TPEKIR | 6 | | 743.44101 | 372.22214 | -5.4 | 2 | 190161.5156 | 0.8 | Medium |
| TPEYETIK | 8 | | 980.4935 | 490.74719 | -6.52 | 2 | | 0.92 | Medium |
| TPPEAI | 6 | | 627.33482 | 627.33307 | -2.79 | 1 | 8535896 | 0.83 | Medium |
| TPSDFE | 6 | | 695.28826 | 695.28662 | -2.36 | 1 | 2032032.875 | 1.53 | High |
| TQALP | 5 | | 529.29804 | 529.29645 | -3.01 | 1 | 1138546.875 | 0.75 | Medium |
| TQAPI | 5 | | 529.29804 | 529.29645 | -3.01 | 1 | 1138546.875 | 0.75 | Medium |
| TQIDIANL | 8 | | 887.48327 | 444.24417 | -2.49 | 2 | | 0.95 | Medium |
| TRIY | 4 | | 552.31402 | 552.31281 | -2.21 | 1 | 2641366.75 | 0.72 | Medium |
| TRPIKIN | 7 | | 841.52541 | 421.26465 | -4.03 | 2 | 548460.25 | 1.29 | Medium |
| TSISAALGM | 9 | 1xOxidation [M9] | 866.4288 | 433.71725 | -1.8 | 2 | 542894.375 | 0.82 | Medium |
| TSTDF | 5 | | 570.24058 | 570.23962 | -1.68 | 1 | 2705750.5 | 0.83 | Medium |
| TTGVTAIGM | 9 | 1xOxidation [M9] | 866.4288 | 433.71725 | -1.8 | 2 | 542894.375 | 0.82 | Medium |
| TTQPDRL | 7 | | 830.43666 | 415.72107 | -2.16 | 2 | 1748187.5 | 1.38 | Medium |
| TTVPVGA | 7 | | 644.36137 | 644.36053 | -1.29 | 1 | 1216345 | 0.97 | Medium |
| TVDFK | 5 | | 609.32425 | 609.32404 | -0.36 | 1 | 3455652.125 | 1.35 | High |
| TVFGA | 5 | | 494.26092 | 494.25946 | -2.96 | 1 | 16496116 | 0.9 | Medium |
| TVFQ | 4 | | 494.26092 | 494.25946 | -2.96 | 1 | 16496116 | 0.9 | Medium |
| TVLLTIAG | 8 | | 787.49238 | 787.49036 | -2.57 | 1 | 471643.4375 | 0.83 | Medium |
| TVNIE | 5 | | 575.30352 | 575.3009 | -4.54 | 1 | 1042037.688 | 1.05 | Medium |
| TVPLEE | 6 | | 687.35595 | 687.3573 | 1.97 | 1 | 10289165 | 0.77 | Medium |
| TVPYE | 5 | | 608.29262 | 608.29163 | -1.63 | 1 | 5312555.5 | 0.86 | Medium |
| TVTI | 4 | | 433.26568 | 433.26413 | -3.57 | 1 | 978312.6875 | 0.7 | Medium |
| TVVGAG | 6 | | 503.28239 | 503.28201 | -0.75 | 1 | 6262132 | 0.73 | Medium |
| VAAGLGSLLH | 10 | | 937.54654 | 469.27493 | -4.22 | 2 | | 1.02 | Medium |
| VAAY | 4 | | 423.22381 | 423.2229 | -2.15 | 1 | 10046891 | 0.79 | Medium |
| VAEVSVEALFVP | 12 | | 1259.68818 | 630.34467 | -4.86 | 2 | | 0.99 | Medium |
| VAFGR | 5 | | 549.31436 | 275.16025 | -2.07 | 2 | 1003845.875 | 1.21 | Medium |
| VAFS | 4 | | 423.22381 | 423.2229 | -2.15 | 1 | 10046891 | 0.79 | Medium |
| VAKLQ | 5 | | 558.36097 | 558.35968 | -2.32 | 1 | 1115697 | 1.42 | High |
| VAID | 4 | | 417.23438 | 417.23325 | -2.71 | 1 | 29495884 | 0.88 | Medium |
| VAIPESR | 7 | | 771.43593 | 771.43298 | -3.82 | 1 | 9400553.875 | 1.32 | High |
| VAIPESRGQ | 9 | | 956.51597 | 478.76022 | -2.93 | 2 | 1204517.125 | 1.4 | Medium |
| VAIT | 4 | | 403.25511 | 403.25415 | -2.38 | 1 | 964773.5 | 0.8 | Medium |
| VAIVM | 5 | 1xOxidation [M5] | 548.31125 | 548.31085 | -0.72 | 1 | 1267634.875 | 1.11 | Medium |
| VAPPARPVPT | 10 | | 1004.58874 | 502.79706 | -1.89 | 2 | 3222333.5 | 1.09 | Medium |
| VAQHMY | 6 | | 748.34467 | 748.34393 | -0.99 | 1 | 1662864 | 1.55 | High |
| VASF | 4 | | 423.22381 | 423.2229 | -2.15 | 1 | 10046891 | 0.79 | Medium |
| VAYA | 4 | | 423.22381 | 423.2229 | -2.15 | 1 | 10046891 | 0.79 | Medium |
| VDGDNAPITA | 10 | | 972.46327 | 972.45923 | -4.15 | 1 | 803227.75 | 1.08 | Medium |
| VDIDPK | 6 | | 686.37193 | 686.36938 | -3.71 | 1 | 1905262.125 | 1.35 | High |
| VDNRGGGF | 8 | | 821.39004 | 411.19812 | -1.31 | 2 | 19905246 | 1.46 | Medium |
| VEEI | 4 | | 489.25551 | 489.25406 | -2.96 | 1 | 2683889.25 | 1.04 | Medium |
| VEGLE | 5 | | 546.27697 | 546.27423 | -5.01 | 1 | 3583164.25 | 0.76 | Medium |
| VEKEV | 5 | | 603.33482 | 603.33331 | -2.49 | 1 | 899166.3125 | 0.83 | Medium |
| VEKIIGR | 7 | | 814.51451 | 407.75977 | -2.77 | 2 | 1749559.5 | 2.04 | High |
| VELAE | 5 | | 560.29262 | 560.2912 | -2.53 | 1 | 425404.5 | 1 | Medium |
| VEPLNPS | 7 | | 755.3934 | 755.39307 | -0.44 | 1 | 696330 | 0.94 | Medium |
| VEPV | 4 | | 443.25003 | 443.24905 | -2.19 | 1 | 4527516.5 | 0.71 | Medium |
| VERDKLNKY | 9 | | 1164.63715 | 582.82123 | -1.69 | 2 | 1037869.375 | 1.73 | Medium |
| VETI | 4 | | 461.26059 | 461.25949 | -2.38 | 1 | 78935728 | 0.91 | Medium |
| VEVPKPG | 7 | | 725.41922 | 363.2121 | -3.17 | 2 | 1968395.5 | 1.37 | Medium |
| VEVPS | 5 | | 530.28205 | 530.28088 | -2.21 | 1 | 1436009.375 | 0.94 | Medium |
| VEVSP | 5 | | 530.28205 | 530.28088 | -2.21 | 1 | 1436009.375 | 0.94 | Medium |
| VEVTT | 5 | | 548.29262 | 548.29199 | -1.14 | 1 | | 0.84 | Medium |
| VGDIE | 5 | | 532.26132 | 532.26056 | -1.43 | 1 | 3222326.75 | 0.79 | Medium |
| VGFR | 4 | | 478.27724 | 478.27609 | -2.41 | 1 | 2838411.75 | 0.7 | Medium |
| VGFTR | 5 | | 579.32492 | 290.1655 | -2.08 | 2 | 3300810.938 | 1.22 | Medium |

| | | | | | | | | | |
|---|---|---|---|---|---|---|---|---|---|
| VGGDIE | 6 | | 589.28278 | 589.28125 | -2.6 | 1 | 1123788.625 | 1.37 | High |
| VGKPI | 5 | | 513.33951 | 513.33844 | -2.08 | 1 | 784074.8125 | 0.83 | Medium |
| VGIGV | 5 | | 444.28166 | 444.28049 | -2.64 | 1 | 109830880 | 0.78 | Medium |
| VGITPQ | 6 | | 614.3508 | 614.34924 | -2.54 | 1 | 497514912 | 0.72 | Medium |
| VGIVG | 5 | | 444.28166 | 444.28049 | -2.64 | 1 | 109830880 | 0.78 | Medium |
| VGNGSDELLR | 10 | | 1059.54291 | 530.27374 | -2.55 | 2 | 15391762 | 1.11 | Medium |
| VGPALG | 6 | | 513.30312 | 513.30194 | -2.3 | 1 | 1998379.75 | 1.03 | Medium |
| VGPLE | 5 | | 514.28714 | 514.28571 | -2.79 | 1 | 16506790 | 1.16 | Medium |
| VGQLEP | 6 | | 642.34572 | 642.3418 | -6.1 | 1 | 1537789.5 | 0.76 | Medium |
| VGRVV | 5 | | 529.34566 | 529.34454 | -2.1 | 1 | 889196.0625 | 0.72 | Medium |
| VGVM | 4 | | 405.21662 | 405.21552 | -2.72 | 1 | 6955801 | 1.08 | Medium |
| VGVM | 4 | 1xOxidation [M4] | 421.21153 | 421.2106 | -2.21 | 1 | 133303.0781 | 0.82 | Medium |
| VGVNYIDPR | 9 | | 1032.54727 | 516.78131 | 7.82 | 2 | 1261546.25 | 1.05 | Medium |
| VGVRV | 5 | | 529.34566 | 529.34454 | -2.1 | 1 | 889196.0625 | 0.72 | Medium |
| VKDF | 4 | | 508.27657 | 508.2746 | -3.89 | 1 | 78960.69531 | 1.06 | Medium |
| VKVY | 4 | | 508.31296 | 508.31165 | -2.59 | 1 | 2549031.25 | 0.93 | Medium |
| VIDRVPQ | 7 | | 826.47813 | 413.74106 | -3.98 | 2 | 1161601.125 | 1.35 | Medium |
| VIGVT | 5 | | 488.30788 | 488.30664 | -2.53 | 1 | 12110439 | 0.89 | Medium |
| VIIM | 4 | 1xOxidation [M4] | 491.28978 | 491.28845 | -2.71 | 1 | 1294633.875 | 0.82 | Medium |
| VIRGPGGVGR | 10 | | 967.57957 | 323.19702 | -3.16 | 3 | 1961466.375 | 2.2 | Medium |
| VIVE | 4 | | 459.28133 | 459.28006 | -2.76 | 1 | 12587389 | 0.71 | Medium |
| VIVSGP | 6 | | 571.34499 | 571.34363 | -2.38 | 1 | 3631437.75 | 0.74 | Medium |
| VIYPN | 5 | | 605.32934 | 605.32831 | -1.7 | 1 | | 0.83 | Medium |
| VMDLGR | 6 | | 690.36032 | 345.68127 | -7.31 | 2 | 15839013 | 0.88 | Medium |
| VMLRGL | 6 | 1xOxidation [M2] | 704.41236 | 704.4173 | 7.01 | 1 | 1421272 | 0.79 | Medium |
| VNLPGA | 6 | | 570.32459 | 570.32288 | -3 | 1 | 1038788 | 1.09 | Medium |
| VNIPQ | 5 | | 570.32459 | 570.32288 | -3 | 1 | 1038788 | 1.09 | Medium |
| VNPGFGGQ | 8 | | 775.37333 | 775.37231 | -1.31 | 1 | 905123.5 | 1.84 | High |
| VNRIP | 5 | | 598.36712 | 598.36554 | -2.64 | 1 | 36809244 | 1.02 | Medium |
| VNRPI | 5 | | 598.36712 | 598.36554 | -2.64 | 1 | 36809244 | 1.02 | Medium |
| VPAALT | 6 | | 571.34499 | 571.34357 | -2.49 | 1 | 3631437.75 | 0.82 | Medium |
| VPASVV | 6 | | 571.34499 | 571.34351 | -2.6 | 1 | 3631437.75 | 0.73 | Medium |
| VPATAL | 6 | | 571.34499 | 571.34357 | -2.49 | 1 | 3631437.75 | 0.8 | Medium |
| VPATIA | 6 | | 571.34499 | 571.34357 | -2.49 | 1 | 3631437.75 | 0.8 | Medium |
| VPEPR | 5 | | 597.33549 | 597.33325 | -3.74 | 1 | 66527128 | 0.72 | Medium |
| VPNPSMVVQ | 9 | 1xOxidation [M6] | 986.49754 | 986.5033 | 5.83 | 1 | 2009639.375 | 0.7 | Medium |
| VPVS | 4 | | 401.23946 | 401.23825 | -3.02 | 1 | 54510248 | 0.83 | Medium |
| VPVVLGMRDSI | 11 | | 1185.66601 | 1185.66809 | 1.76 | 1 | 1325512.25 | 0.73 | Medium |
| VQDVIND | 7 | | 802.39412 | 802.39142 | -3.37 | 1 | 888926.4375 | 0.97 | Medium |
| VQGHY | 5 | | 603.28854 | 603.28754 | -1.66 | 1 | 653699.375 | 0.8 | Medium |
| VQPPKL | 6 | | 681.42939 | 341.21762 | -2.09 | 2 | 4567528 | 0.8 | Medium |
| VQPRPA | 6 | | 667.38858 | 667.38531 | -4.9 | 1 | | 0.83 | Medium |
| VQVPS | 5 | | 529.29804 | 529.29675 | -2.43 | 1 | 3637882.25 | 0.89 | Medium |
| VRAIE | 5 | | 587.35114 | 294.17896 | -0.86 | 2 | 837586.125 | 1.04 | Medium |
| VRVDLK | 6 | | 729.46175 | 365.23386 | -1.8 | 2 | 810275.5625 | 1.27 | Medium |
| VSDLR | 5 | | 589.3304 | 295.16809 | -2.54 | 2 | 911076.0625 | 0.94 | Medium |
| VSDPY | 5 | | 580.26132 | 580.25891 | -4.15 | 1 | 4895474 | 0.72 | Medium |
| VSGLIAAIPV | 10 | | 939.58734 | 939.58691 | -0.46 | 1 | 641906618 | 0.71 | Medium |
| VSGVF | 5 | | 508.27657 | 508.2746 | -3.89 | 1 | 78960.69531 | 1.09 | Medium |
| VSIIR | 5 | | 587.38752 | 294.1969 | -1.7 | 2 | 9191945 | 1.07 | Medium |
| VSIRPDE | 7 | | 815.42576 | 408.21527 | -3.06 | 2 | 24514921.25 | 1.6 | Medium |
| VSPQPGVPPEE | 11 | | 1135.56298 | 568.28326 | -3.28 | 2 | 1659337.188 | 1.5 | Medium |
| VSTRSPR | 7 | | 802.45298 | 401.72665 | -8.65 | 2 | | 0.86 | Medium |
| VTAI | 4 | | 403.25511 | 403.25394 | -2.91 | 1 | 10495452 | 0.97 | Medium |
| VTEKVENPQ | 9 | | 1043.53677 | 522.27631 | 8.21 | 2 | 2301548.5 | 0.8 | Medium |
| VTFQ | 4 | | 494.26092 | 494.25946 | -2.96 | 1 | 16496116 | 0.9 | Medium |
| VTNIE | 5 | | 575.30352 | 575.3009 | -4.54 | 1 | 1042037.688 | 1.05 | Medium |
| VTPIEE | 6 | | 687.35595 | 687.3573 | 1.97 | 1 | 10289165 | 0.77 | Medium |
| VTTI | 4 | | 433.26568 | 433.26413 | -3.57 | 1 | 978312.6875 | 0.7 | Medium |
| VTVGQ | 5 | | 503.28239 | 503.28201 | -0.75 | 1 | 6262132 | 0.73 | Medium |
| VTVLR | 5 | | 587.38752 | 294.1969 | -1.7 | 2 | 9191945 | 1.12 | Medium |
| VTVPAG | 6 | | 543.31369 | 543.31274 | -1.74 | 1 | 2929527.5 | 1.01 | Medium |
| VTVPQ | 5 | | 543.31369 | 543.31274 | -1.74 | 1 | 2929527.5 | 1.01 | Medium |
| VVAFLER | 7 | | 833.48796 | 417.24405 | -8.57 | 2 | | 0.96 | Medium |
| VVAI | 4 | | 401.27585 | 401.27448 | -3.42 | 1 | 12248918 | 0.82 | Medium |
| VVAY | 4 | | 451.25511 | 451.25381 | -2.87 | 1 | 4218747 | 0.77 | Medium |
| VVDM | 4 | | 463.2221 | 463.22079 | -2.81 | 1 | 3978154.5 | 1.06 | Medium |
| VVFH | 4 | | 501.28199 | 501.28085 | -2.28 | 1 | 63534388 | 1.12 | Medium |
| VVGGAGAAAGLGELGAV | 17 | | 1368.74815 | 456.91974 | -2.55 | 3 | 10341643 | 1.68 | Medium |
| VVIA | 4 | | 401.27585 | 401.27448 | -3.42 | 1 | 12248918 | 0.82 | Medium |
| VVLNPG | 6 | | 598.35589 | 598.35443 | -2.43 | 1 | 1780364.25 | 0.82 | Medium |
| VVPKIR | 6 | | 711.48757 | 356.24661 | -2.28 | 2 | 4731436.906 | 1.22 | Medium |
| VVPPAGV | 7 | | 638.38719 | 638.38623 | -1.5 | 1 | 669429.6875 | 0.71 | Medium |
| VVPPAVG | 7 | | 638.38719 | 638.38623 | -1.5 | 1 | 669429.6875 | 0.71 | Medium |
| VVPPAVGG | 8 | | 695.40865 | 695.40729 | -1.96 | 1 | 717245.0625 | 1.3 | High |
| VVPPAVGGAN | 10 | | 880.48869 | 880.48785 | -0.95 | 1 | 2714488.25 | 1.39 | High |
| VVTIR | 5 | | 587.38752 | 587.38635 | -1.99 | 1 | 6559271 | 1.22 | High |
| VVWVLR | 6 | | 771.48757 | 257.83185 | -8.53 | 3 | | 1.29 | Medium |
| VYAAF | 5 | | 570.29223 | 570.29401 | 3.12 | 1 | 8964888 | 0.89 | Medium |
| VYKNCN | 6 | | 740.33959 | 370.67639 | 8 | 2 | | 0.8 | Medium |
| WCGLGW | 6 | | 721.31264 | 721.31274 | 0.14 | 1 | 3219601.5 | 0.78 | Medium |
| WDIP | 4 | | 530.26092 | 530.26471 | 7.14 | 1 | | 0.74 | Medium |
| WDPI | 4 | | 530.26092 | 530.26471 | 7.14 | 1 | | 0.74 | Medium |

| | | | | | | | | | |
|---|---|---|---|---|---|---|---|---|---|
| WGAANALSAG | 10 | | 917.44756 | 917.44519 | -2.58 | 1 | 1030677 | 0.75 | Medium |
| WGMY | 4 | | 556.22243 | 556.22791 | 9.84 | 1 | | 0.71 | Medium |
| WKHSYTPI | 8 | | 1031.53089 | 516.26514 | -7.65 | 2 | | 0.99 | Medium |
| WNGPM | 5 | 1xOxidation [M5] | 620.24971 | 620.24689 | -4.55 | 1 | 888494.9375 | 0.86 | Medium |
| WSTPH | 5 | | 627.28854 | 314.14731 | -1.91 | 2 | 1254031.75 | 0.81 | Medium |
| YAIEPK | 6 | | 720.39267 | 720.39099 | -2.33 | 1 | 8677017 | 1.32 | High |
| YAVAG | 5 | | 480.24527 | 480.24368 | -3.31 | 1 | | 0.8 | Medium |
| YAVGA | 5 | | 480.24527 | 480.24368 | -3.31 | 1 | | 0.8 | Medium |
| YAVGG | 5 | | 466.22962 | 466.22797 | -3.56 | 1 | 2565986.75 | 0.82 | Medium |
| YAVN | 4 | | 466.22962 | 466.22797 | -3.56 | 1 | 2565986.75 | 0.82 | Medium |
| YAVQ | 4 | | 480.24527 | 480.24368 | -3.31 | 1 | | 0.8 | Medium |
| YCTNPVCPKPS | 11 | | 1208.54384 | 604.77716 | 2.65 | 2 | 1071131.375 | 0.88 | Medium |
| YDAIN | 5 | | 595.27222 | 595.27069 | -2.56 | 1 | 21742782 | 0.71 | Medium |
| YDIR | 4 | | 566.29329 | 566.29193 | -2.4 | 1 | 122610172 | 1.06 | Medium |
| YDYVGN | 6 | | 730.30425 | 730.30237 | -2.57 | 1 | 15105817.19 | 1.16 | Medium |
| YEQIR | 5 | | 708.36752 | 354.68655 | -2.38 | 2 | 24014950 | 0.89 | Medium |
| YFADR | 5 | | 671.31475 | 336.16019 | -2.46 | 2 | 88710328 | 1.22 | Medium |
| YFDRA | 5 | | 671.31475 | 336.16003 | -2.92 | 2 | 118115230 | 1.12 | Medium |
| YFRPI | 5 | | 695.38752 | 695.38184 | -8.18 | 1 | 1233173.25 | 0.8 | Medium |
| YFWADIAGGDAR | 12 | | 1341.62223 | 671.31232 | -3.63 | 2 | 29404902 | 0.83 | Medium |
| YGGDI | 5 | | 524.2351 | 524.23303 | -3.95 | 1 | 1443928.375 | 0.87 | Medium |
| YGGGY | 5 | | 516.20889 | 516.20764 | -2.42 | 1 | 8610282 | 0.78 | Medium |
| YGGSL | 5 | | 496.24019 | 496.23877 | -2.86 | 1 | 4365559.5 | 0.72 | Medium |
| YGIAF | 5 | | 570.29223 | 570.29425 | 3.55 | 1 | 8964888 | 0.7 | Medium |
| YGILYA | 6 | | 699.3712 | 699.37311 | 2.72 | 1 | 1530515 | 0.99 | Medium |
| YGIT | 4 | | 453.23438 | 453.23325 | -2.49 | 1 | 3249804 | 0.72 | Medium |
| YHNI | 4 | | 546.26707 | 273.63647 | -2.56 | 2 | 925069 | 0.92 | Medium |
| YLAGR | 5 | | 579.32492 | 290.16528 | -2.82 | 2 | 5779612 | 0.89 | Medium |
| YIDPE | 5 | | 636.28753 | 636.28577 | -2.78 | 1 | 4423733.5 | 1.02 | Medium |
| YIDSPE | 6 | | 723.31956 | 723.31915 | -0.57 | 1 | 8354254.5 | 1.22 | High |
| YIGAR | 5 | | 579.32492 | 290.16528 | -2.82 | 2 | 5779612 | 0.89 | Medium |
| YIGGR | 5 | | 565.30927 | 283.15793 | -1.22 | 2 | 318946.2813 | 0.85 | Medium |
| YINR | 4 | | 565.30927 | 283.15793 | -1.22 | 2 | 318946.2813 | 0.85 | Medium |
| YIQR | 4 | | 579.32492 | 290.16528 | -2.82 | 2 | 5779612 | 0.89 | Medium |
| YLRGLIYSENLK | 12 | | 1468.81584 | 490.27859 | 3.67 | 3 | 726929.375 | 1 | Medium |
| YITPG | 5 | | 550.28714 | 550.28473 | -4.38 | 1 | 2533078.5 | 0.81 | Medium |
| YNDI | 4 | | 524.2351 | 524.23303 | -3.95 | 1 | 1443928.375 | 0.87 | Medium |
| YNGY | 4 | | 516.20889 | 516.20764 | -2.42 | 1 | 8610282 | 0.78 | Medium |
| YNSI | 4 | | 496.24019 | 496.23877 | -2.86 | 1 | 4365559.5 | 0.72 | Medium |
| YPTYPN | 6 | | 754.34063 | 754.33887 | -2.34 | 1 | 14874405 | 1.38 | High |
| YRAGI | 5 | | 579.32492 | 579.32349 | -2.48 | 1 | 4063479.75 | 0.81 | Medium |
| YRGAI | 5 | | 579.32492 | 579.32349 | -2.48 | 1 | 4063479.75 | 0.81 | Medium |
| YRGGI | 5 | | 565.30927 | 283.15747 | -2.84 | 2 | 429538.0313 | 0.97 | Medium |
| YRNI | 4 | | 565.30927 | 283.15747 | -2.84 | 2 | 429538.0313 | 0.97 | Medium |
| YRPR | 4 | | 591.33616 | 296.1709 | -2.77 | 2 | 1271918.781 | 0.88 | Medium |
| YRQI | 4 | | 579.32492 | 579.32349 | -2.48 | 1 | 4063479.75 | 0.81 | Medium |
| YSDLLAHFQ | 9 | | 1093.53129 | 547.26556 | -6.8 | 2 | 16737080.13 | 1.8 | Medium |
| YTPDYTPK | 8 | | 984.46729 | 984.46466 | -2.67 | 1 | 18609426.25 | 2.62 | High |
| YVAAF | 5 | | 570.29223 | 570.29401 | 3.12 | 1 | 8964888 | 0.89 | Medium |
| YVDAF | 5 | | 614.28205 | 614.28345 | 2.27 | 1 | 7283693.5 | 0.89 | Medium |
| YVIK | 4 | | 522.32861 | 522.32788 | -1.4 | 1 | 2562120.5 | 1.03 | Medium |
